# The expression patterns of immune response genes in the Peripheral Blood Mononuclear cells of pregnant women presenting with subclinical or clinical HEV infection are different and trimester-dependent: A whole transcriptome analysis

**DOI:** 10.1371/journal.pone.0228068

**Published:** 2020-02-03

**Authors:** Ashwini Y. Ramdasi, Vidya A. Arankalle

**Affiliations:** Hepatitis Division, ICMR- National Institute of Virology, Pune, Maharashtra, India; INSERM, FRANCE

## Abstract

Hepatitis E is an enteric disease highly prevalent in the developing countries. The basis for high mortality among pregnant hepatitis E patients remains unclear. Importantly, a large proportion of infected pregnant women present with subclinical infection as well. In order to understand the possible mechanisms influencing clinical presentation of hepatitis E in pregnant women, we explored a system biology approach. For this, PBMCs from various categories were subjected to RNAseq analysis. These included non-pregnant (NPR, acute and convalescent phases) and pregnant (PR, 2^nd^ and 3^rd^ trimesters, acute phase and subclinical HEV infections) patients and corresponding healthy controls. The current study deals with immune response genes.

In contrast to exclusive up-regulation of nonspecific, early immune response transcripts in the NPR patients, the PR patients exhibited broader and heightened expression of genes associated with innate as well as adaptive T and B cell responses. The study identified for the first time (1) inverse relationship of immunoglobulin (Ig) genes overexpression and (2) association of differential expression of S100 series genes with disease presentation. The data suggests possible involvement of TLR4 and NOD1 in pregnant patients and alpha defensins in all patient categories suggesting a role in protection. Induction of IFNγ gene was not detected during the acute phase irrespective of pregnancy. Association of response to vitamin D, transcripts related to NK/NKT and regulatory T cells during subclinical infection are noteworthy.

The data obtained here could be correlated with several studies reported earlier in hepatitis E patients suggesting utility of PBMCs as an alternate specimen. The extensive, informative data provided here for the first time should form basis for future studies that will help in understanding pathogenesis of fulminant hepatitis E.

## Introduction

Hepatitis E virus (HEV) causes waterborne epidemics and is the major contributor of sporadic acute viral hepatitis in adults from developing countries [1–5]. India is hyperendemic to Hepatitis E and sporadic as well as epidemic HEV infections are exclusively caused by genotype 1 HEV [6–8]. The disease is usually self-limiting with ≤ 0.5% mortality. However, an exceptionally high mortality (~20%) is recorded in pregnant women, especially in the later trimesters [9]. In India, fulminant hepatitis E in nonpregnant women and men has been recorded [10]. A recent report documented severity of hepatitis E in pregnant women from developed countries [11]. So far, chronic hepatitis E is restricted to immuno-compromised patients from the industrialized nations [12–14].

Early symptoms and clinical features among pregnant patients are similar to nonpregnant patients, however, there is a high risk of rapid progression to poor maternal, obstetric and foetal outcome. These include deranged coagulogram, encephalopathy, death, intra-uterine death and premature deliveries [15–19]. Understanding mechanism(s) of disease severity and mortality in pregnant women is of utmost importance and relevance for the endemic countries. However, pathogenesis of hepatitis E in general and during pregnancy in particular is not well understood. This is primarily because of the lack of small laboratory animal model and inability of nonhuman primate models to replicate fulminant disease during pregnancy [20,21]. Recent reports of pregnant rabbit and BALB/c models may prove useful in understanding the basis for observed complications of HEV infection [22,23].

Association of viral load with disease severity is controversial [24,25] and host response seems to be primarily responsible for differential outcomes of HEV infection. TLRs have been evaluated in hepatitis E patients with pregnancy. Contrary to the temporal activation of TLR4/TLR7/TLR8 at protein and mRNA levels in the NPR patients, the ANC-patients and controls exhibited reduced TLRs indicative of impaired TLR response [26]. Importantly, a significant reduction was recorded in the fulminant category [27]. The expression of TLR3 was associated with recovery while the patients with lower expression progressed to acute liver failure. A significant downregulation of TLR3 and TLR9 and downstream MYD88 signaling molecules IRF3 and IRF7 was shown in pregnant women with FHF-E than with acute hepatitis E [28].

As against the classical activation of CD14^+^ monocytes in the non-ANC patients, impaired response was recorded in the ANC-patients while the CD4^+^ T cell populations were similar in both patient groups [29]. Though cytokine levels have been measured either in serum or HEV protein stimulated PBMCs from pregnant women, no conclusions can be made about the causal role of these molecules [30–33]. Upregulation of PBMC miR-450b correlated with poor outcome of HEV during pregnancy [34].

Our observations of hepatitis E in pregnancy are twofold. First, confirmation of high mortality during later pregnancy and extending the etiology of fulminant hepatitis to non-pregnant women and men [10]; Second, the observation of large proportion of pregnant women infected with HEV develop subclinical infection, the ratio of clinical: subclinical ratio in the later trimesters being 1:13 [35].Thus, HEV infection in pregnant women also leads to very mild symptoms and we believe that the understanding of basis for such milder infections is as important as the fulminant disease form with high mortality.

In view of the time dependent immunologic changes during pregnancy [36–38] and the differential effect of HEV infection, we thought that a system biology approach may help us in getting insight in the alterations caused by the virus, pregnancy and pregnancy with virus infection. RNA-seq is a powerful tool that provides unbiased, simultaneous profiles of gene expression and is particularly suitable to get an overview of differential, complex interactions of a very large number of genes. Such a global analysis is likely to identify mechanisms that need to be addressed further in terms of functional analyses.

Though liver is an ideal organ for studies of hepatotropic viruses, this organ cannot be accessed in population groups needed for such comparisons. We therefore opted for PBMCs as the test specimen with easy accessibility and for the first time present whole transcriptome analysis in non-pregnant patients and pregnant women with clinical or subclinical presentation.

### Patients and methods

#### Ethics statement

The study was **approved by the “Institutional Human Ethics Committee”,** of the National Institute of Virology (NIV), India. The NIV is invited by various state governments / local health authorities to investigate epidemics of viral diseases, including hepatitis. During epidemics of hepatitis E, a written informed consent is obtained from all the study subjects by the local health authorities / National Institute of Virology. The healthy pregnant women were bled on the request of the health authorities for the identification of IgM-anti-HEV positives so that they can be monitored for the symptoms and severity of the disease.

#### Study subjects and clinical data

Table 1 provides details of the study population. The diagnosis of hepatitis E was based on the presence of anti-HEV-IgM antibodies in ELISA [7] and only IgM-anti-HEV positives were included in the study. Patients examined within two weeks of the onset of clinical symptoms were grouped as acute-phase while convalescent phase patients were studied during 4–5 weeks post-disease onset. The study groups included (A) healthy non-pregnant controls (NPR-control), (B) healthy,trimester-1 pregnant controls (PR-1-control), (C) healthy, trimester-2 pregnant controls (PR-2-control), (D) healthy, trimester-3 pregnant controls (PR-3-control), (E) non-pregnant patients (NPR) during early acute phase (NPR-acute), (F) NPR patients during convalescence (NPR-conv), (G) pregnant patients, trimester-2, early acute phase (PR-2-acute), (H) pregnant patients, trimester-3, early acute phase (PR-3-acute), (I) pregnant patients, trimester-1,sub-clinical (PR-1-SC), (J) pregnant patients, trimester-2, sub-clinical (PR-2-SC) and (K) pregnant patients, trimester-3, sub-clinical (PR-3-SC). During the study period, we did not identify any trimester-1 pregnant woman with clinical hepatitis E. Groups B and I were included only for “Principal component and correlation” analyses and not for further detailed analysis.

**Table 1:** Characteristics of the patients investigated.

Group	NPR-control(A)	PR-2-control(C)	PR-3-control(D)	NPR-acute(E)	NPR-conv(F)	PR-2-acute(G)	PR-3-acute(H)	PR-2-SC(J)	PR-3-SC(K)
**Total nos.**	16	16	16	28	8	16	16	8	16
**Age (mean ± Std error)**	21 ± 0.4	23 ±1.0	25 ± 2.2	24 ± 1.9	26 ± 3.2	22 ± 0.7	21 ± 1.1	21 ± 0.63	21 ± 0.46
**Serum ALT U/L(mean ± Std error)**	26.38 ± 1.4	21 ± 1.2	19.40 ±1.5	317.32 ± 55.4	196.63 ± 66.8	192.63 ± 5.9	108.44 ± 14.2	81.50 ± 38.98	53.13 ± 22.44
**Bilirubin mg/dl (mean ± Std error)**	0.33 ± 0.09	0.21 ± 0.02	0.19 ± 0.28	2.75 ± 0.56	1.50 ± 0.76	3.0 ± 1.09	3.66 ± 1.20	0.25 ± 0.16	0.75 ± 0.51
**Anti-HEV Log IgM titres (mean ± Std error)**	NA	NA	NA	3.72 ± 0.1	3.67 ± 0.2	3.50 ± 0.3	2.96 ± 0.4	3.00 ± 0.1	2.56 ± 0.2
**Anti-HEV Log IgG titres (mean ± Std error)**	NA	NA	NA	3.8 ± 0.1	4.1 ± 0.1	4.1 ± 0.0	3.4 ± 0.3	3.70 ± 0.17	3.31 ± 0.19
**Serum Protein gm/dl(mean ± Std error)**	6.5±0.2	5.5±0.2	4.9 ± 0.5	5.9 ± 0.2	6.2 ± 0.3	4.5 ± 0.3	4.4 ± 0.3	5.9 ± 0.3	4.8 ± 0.3
**Serum Albumin gm/dl(mean ± Std error)**	3.3 ± 0.1	2.2 ± 0.1	2.0 ± 0.0	2.8 ± 0.8	2.9 ± 0.2	1.9 ± 0.1	1.7 ± 0.1	2.54 ± 0.14	1.83 ± 0.10
**Prolactin ng/ml (mean ± Std error)**	NA	82.9 ± 15.3	141.1 ± 27.4	NA	NA	35.6 ± 10.0	162.7 ± 14.4	141.0 ± 15.2	144.8 ± 14.6
**Beta-HCG mIU/ml(mean ± Std error)**	NA	14778 ± 1152	13215 ± 5639	NA	NA	16223 ± 883	12799 ± 1997	12731 ± 1292	12171 ± 1071
**Progesterone ng/ml** **(mean ± Std error)**	NA	34.7 ± 2.0	54.8 ± 9.3	NA	NA	32.9 ± 5.5	39.1 ± 0.9	38.2 ± 1.2	39.0 ± 0.7

The study population was identified during 3 epidemics of hepatitis E in the rural areas of the state of Maharashtra, India (2008–2010). Representative acute phase serum samples from these epidemics were subjected to HEV-specific PCR, sequencing and phylogenetic analysis [39]. All the samples belonged to genotype-1. Two types of apparently healthy anti-HEV antibody negative control groups included (1) Non-pregnant subjects (n = 16) and (2) Pregnant women in the second and third trimesters. All the study subjects were screened for IgG and IgM-anti-HEV antibodies, IgM-anti-HAV antibodies, HBsAg, IgM-anti-HBc and anti-HCV antibodies (ELISA, Abbott, USA). A detailed clinical examination was done for all the acute viral hepatitis (AVH) cases. All AVH-E patients had typical symptoms of acute viral hepatitis, such as sudden onset of fever, nausea, vomiting, weakness and jaundice. A subclinical case was defined as an IgM anti-HEV positive with or without elevated ALT levels, no clinical symptoms at the time of first sampling and no development of symptoms up to 2 months follow-up.

#### Anti-HEV antibodies and biochemical parameters

The titres of anti-HEV antibodies were determined by two-fold dilutions of the sera and testing in ELISA [16]. Plasma biochemical parameters and pregnancy hormones were quantitated employing Dimension RxL Max (Siemens Healthcare, 127 USA) and Architect (Abbott, USA) platforms respectively.

#### Statistical analysis

Anti-HEV antibodies titres, plasma biochemical parameters and pregnancy related hormone levels were presented as mean ± SE. For all analyses, a p value of < 0.05 derived from a two tailed Mann-Whitney test was considered significant. All statistical analyses were performed with ‘SPSS11.0 for Windows’ software (SPSS Inc.).

#### Transcriptome profiling

**RNA isolation**

Fresh blood samples were collected from all the study subjects in 10ml EDTA vacutainers (BD Biosciences, USA). Plasma and PBMCs were separated within 4 hrs of collection by Ficoll-hypaque gradient method. The PBMCs from all the study groups were immediately stored after isolation in RNA LATER at -80°C till further use. Total RNA was isolated from PBMCs using RiboPure kit (Ambion, Life Technologies USA) and was subjected to quantitation and quality analysis using Nanodrop 1000A spectrophotometer and Bioanalyser (Agilent Technologies, U.S.A.) respectively. Samples exhibiting high quality total RNA with RIN value >9 were selected for further processing. Four RNA samples from a group were pooled (total RNA 2.5μg per sample).

Single-end sequencing of whole transcriptome barcoded libraries from 200–500 ng of rRNA-depleted total RNA isolated from PBMCs was performed on SOLID V4.0 (Sequencing by Oligo Ligation and Detection) analyser, Life Technologies, USA. Briefly, mRNA enrichment from total RNA was performed by rRNA depletion method using RiboMinus Eukaryote kit v2. To each of the rRNA-depleted total RNA sample pools, 1μl of 1:10 dilution of ERCC RNA Spike-In Control Mixes (Ambion USA) reagent was added to assess the platform dynamic range and lower limits of detection.

#### cDNA synthesis and RNA-Seq

Whole transcriptome c-DNA bar-coded libraries were prepared using 200–500 ng rRNA-depleted total RNA and SOLiD Total RNA-Seq Kit, (Life Technologies USA). The barcoded libraries from each study group were combined in color-balanced multiples of four at equimolar concentrations in every multiplex sequencing pool to preserve color balance for the SOLiD System sequencing run. An emulsion PCR was set up of Multiplex sequencing pooled library template at 0.7 pM concentration as per the guidelines using SOLiD EZ Bead E80 System kit (Life Technologies USA) as per the manufacturer’s instructions. 3’ end of P2-enriched beads were modified for each sample and volume of beads was adjusted approximately 750,000beads/μL to 1.25 million beads/μL prior to loading on SOLiD platform.

#### Data analysis

A company (OcimumBiosolutions Hyderabad, India) specialized in the analysis of data generated using NGS platforms was engaged for complete analysis according to the types of comparisons specified by us.

The analysis included:

- Preliminary assessment of raw reads
- Read quality enhancement by filtering ribosomal RNA and mapping to human reference genome.
- Transcript abundance estimation and differential expression analysis
- The whole transcriptome data analysis process involved checking the read quality, mapping the reads to reference sequence and differential gene expression analysis.
- Quality analysis of RNA reads was done through FastQC tool. Quality analysis of RNA reads across read positions and overall data quality across the samples was analyzed. High quality reads (Q>20) were 50–69% across the samples tested, which corresponds 99% inferred base call accuracy. Reads were initially mapped to ribosomal RNA sequences (5, 5.8, 12, 16, 18 and 28s) using Bowtie-0.12.8 with default settings. Reads that mapped to ribosomal sequences were excluded from further analysis (S1 Table). Remaining reads were mapped to human reference genome hg19, ENSEMBL release GRCh37.68 using Tophat-2.0.5. Quality control metrics such as read summary, coverage and transcript associated reads were computed using RNASeQC. S1 and S2 Tables show mapping summary to the hg19 genome and exonic rates respectively.13-86 million (14.6–49.3% of total) reads mapped to human genome (hg19), of which 77–88% were uniquely mapped. All the samples exhibited high exonic rate (61–88% of uniquely mapped reads were mapped to exonic regions). ~ 15,000–22,000 genes were detected with >5 unique reads across samples. On an average, 32 million reads were available for expression analysis. Principal component analysis (PCA) and correlation plots were obtained on log10 FPKM values and a coefficient value close to 1.0 was obtained indicating highly linear relation between the samples.

Reads were also mapped to ERCC sequences using Bowtie-0.12.8 ERCC RNA Spike-In control concentration values were transformed as per volume and dilution, which is 1 μL (1:10) for 300-400ng rRNA depleted RNA. ERCC Spike-in Log2 transformed concentration values and FPKM values were used to obtain dose response plots. All samples showed correlation value close to 2.0. Cufflinks v2.0.2 program was used to get transcript abundance estimates in terms of FPKM values. Expression levels were calculated based on kilobase of exon per million mapped fragments (FPKM). To assess replicate similarity and to identify outlier samples, PCA and correlation plots were obtained on log10 FPKM values using R package. Differential expression analysis was done for all pair-wise comparisons using Cuffdiff (Cufflinks v2.0.2) and Cummerbund package in GenePattern. Variances in the expression levels were calculated using negative binomial distribution. P-values were adjusted with Benjamini-Hochberg’s FDR correction. A gene/transcript was identified to be significantly differentially expressed if FDR corrected p-value was < = 0.1. For comparative transcriptome analysis, only significantly differentially expressed gene/transcripts with fold change > = (+/-) 2.0 were considered for the downstream analysis. Mean fold change value was calculated for each group and used for group comparisons. The gene ontology and pathway analyses were carried out using Database for Annotation, Visualization and Integrated Discovery (DAVID) and KEGG database. The differentially expressed genes (DEG) in all the study groups were enriched based on function using Gene Ontology (GO) enrichment in DAVID with a EASE score cut-off <0.05. KEGG Pathways with at least two genes and P value < = 0.05 were considered as “associated” with respective groups examined. P-value is Modified Fisher's exact test p-value or EASE Score. The sequences generated during this study are uploaded in NCBI-Short Read Archive (SRA) under the Bioproject SRP100353.

#### Real Time-PCR validation

Real Time-PCR validation was performed using individual TaqMan primer probes and SYBR green-based assays for selected genes. For TaqMan assay, 30 differentially expressed genes (DEGs) and 18S rRNA as the endogenous control were selected. These included: chemokines /receptors and cytokines (CCL2, CCL3, CX3CR1,CXCR4, CCL3L3,CCR2, PF4), pattern recognition receptor (TLR8), proinflammatory cytokine (IL1B, IL8), Th-1 cytokine (IFNγ), cell adhesion molecule involved in T-cell activation (ICAM1), defense response to pathogens (DEFA4, ELANE, CAMP,PLAUR,TICAM1,DNASE2, IER3), transcription factors (JUN, PLAGL2, ZNF70), mitochondrial apoptotic inducer (IFI27), immunoglobulin gene (IGJ), co-stimulatory molecules (CD37, CD48 and PRDX5,WDTC1). TaqMan primer-probes were synthesized from Life Technologies, Thermofisher, USA. For SYBR green-based assay (FastStart Universal SYBR Green master mix (ROX)(Roche), antimicrobial peptides defensins (DEFA1 and DEFA4), S100 molecules (S100A6, S100A8, S100A9 and S100A12), immunoglobulin (IGJ) genes and 18S rRNA as the endogenous control were selected. Representative RNA pools were reverse transcribed using High capacity c-DNA kit (Life Technologies, USA) and further used for PCR amplification on 7300 Real Time PCR machine for both the assays. Relative gene expression values were obtained employing comparative Ct method using Applied Biosystems’ 7300 System SDS software. cDNAs from healthy NPR and PR controls were considered as calibrators for analyzing the relative gene expression in all the patient categories. Primers used for SYBR green-based assay are enlisted in (S14 Table)

## Results

### Patient demographic characteristics (Table 1)

The mean duration of the onset of clinical symptoms and blood collection, Post Onset Day of sample collection (POD) was 5.7 ± 0.8 days (NPR-acute), 6.6±1.8 and 5±0.8 (PR-2-acute and PR-3-acute) and 34.5 ± 2.1 days (NPR-conv). The patients were negative for IgM-anti-HAV, HBsAg / IgM-anti-HBc and anti-HCV antibodies while the controls were negative for all these markers as well as IgM and IgG anti-HEV antibodies. All the patients enrolled under the present study recovered uneventfully.

A detailed clinical examination was done for all the AVH cases. Non-pregnant and pregnant-controls exhibited normal liver and kidney functions.ALT levels were raised in non-pregnant and pregnant patients and were higher than in the sub-clinical PR-2-SC and PR-3-SC categories. Bilirubin levels were raised and comparable among pregnant and non-pregnant patients and normal in both the subclinical groups. The levels of creatinine, urea and globulins were within normal range in all the groups examined.

In PR-2 patients, prolactin levels were higher in subclinical and lower in clinical HEV infection than in the controls whereas no significant change in the levels of progesterone and HCG was observed. Titres of IgM and IgG anti-HEV antibodies were not different among different patient groups.

#### Transcriptome analyses

**Fig 1:**
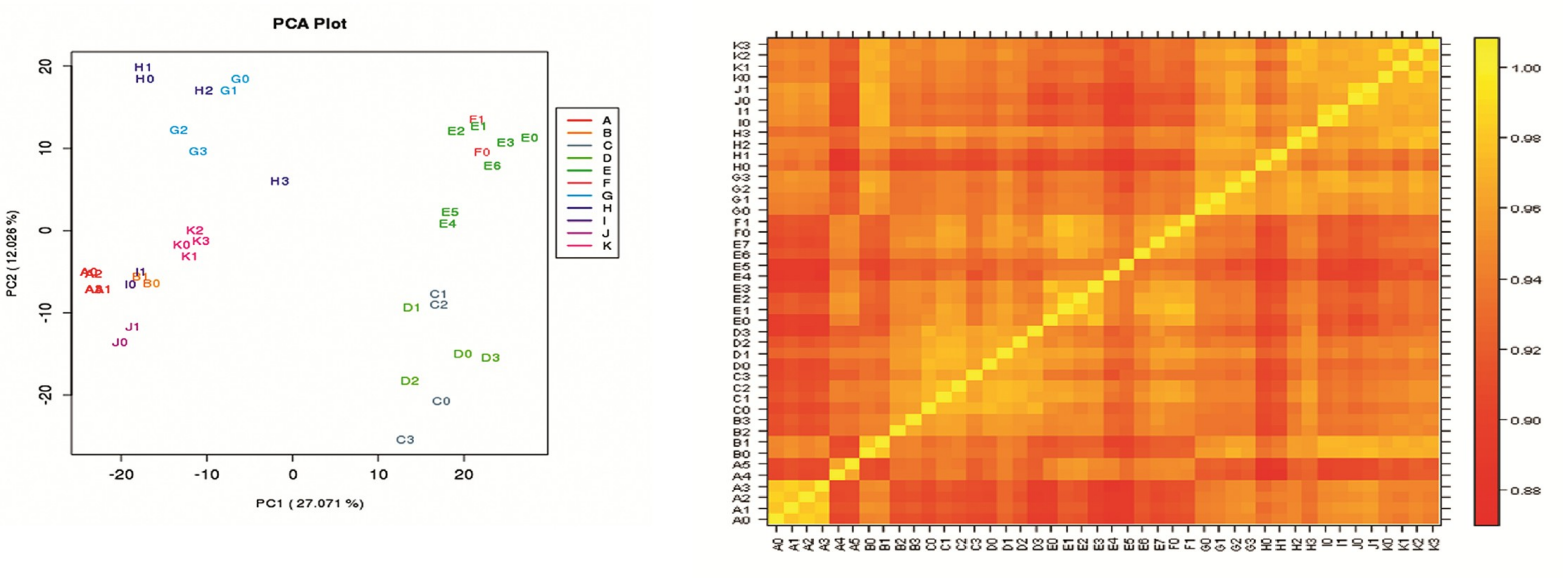
**A** Principal Component Analysis (PCA) to assess the variation among all samples under study. PCA was done on log10FPKM values to assess sample similarity in each group and to identify outlier samples. (Tools: Base package of R). The alphabets in the plot indicate study groups. The proportion of the variance explained by the principal components is indicated in parentheses. The study groups are: A = NPR-controls, B = PR-1-controls, C = PR-2-controls, D = PR-3-controls, E = NPR-acute, F = NPR-conv, G = PR-2-acute, H = PR-3-acute, I = PR-1-SC, J = PR-2-SC and K = PR-3-SC. All the samples within groups are intact and there are no outliers. (Data for the first trimester i.e., groups B and I is not included in the present study). **Fig 1B** Correlation Plot of all samples under study. Correlation analysis was performed on log10FPKM values. The plot shows the pair-wise Pearson's correlation coefficients between the expression values of the samples. Pairs of samples coming from the same group showed high correlation values (Tools: Lattice package of R). The alphabets in the plot indicate study groups. The scale bar indicates Pearson's correlation coefficients.The study groups are: The study groups are:(A):NPR-control, (B):PR-1-control, (C):PR-2-control, (D):PR-3-control, (E):NPR-acute, (F):NPR-conv, (G):PR-2-acute, (H):PR-3-acute, (I):PR-1-SC, (J):PR-2-SC and (K):PR-3-SC. All the samples within groups are intact and there are no outlier samples. (Data generated from groups B and I is not included in this study).

To obtain an initial overview of gene expression patterns, we performed principal component analysis that clearly separated data between HEV infections in non-pregnant and pregnant patients in all the three trimesters and normal non-pregnant and pregnant controls (Fig 1A). In addition, samples within a study group showed high degree of similarity as documented by Pearson’s Correlation analysis(Fig 1B).As evident from the heatmap (S1 Fig), the acute disease was distinctly different, PR-2-acute and PR-3-acute separating from NPR-acute patients. The 2^nd^ cluster was divided into trimester-dependent subclinical infections and pregnant controls. Clearly, the hierarchical clustering was based on HEV infection type and pregnancy duration. For this study, our analysis is restricted to immune response only. In view of the higher mortality during later trimesters, only 2^nd^ and 3^rd^ trimester were studied further.

#### Differences in host gene transcript abundance between the acute and convalescent phases of HEV infection in the NPR patients

**Fig 2:**
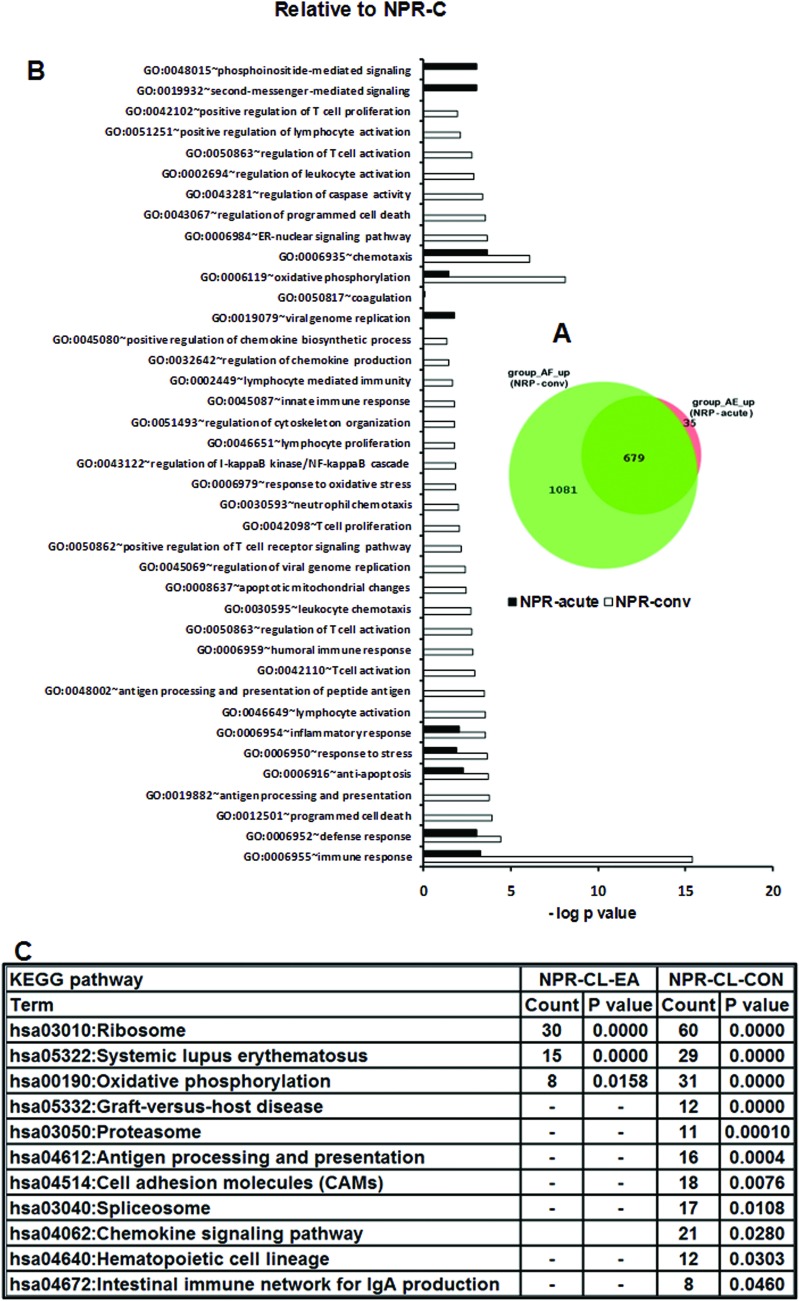
Changes in gene expression and biologic pathways affected following HEV infection in acute and convalescent non-pregnant patients with reference to the non-pregnant controls. Differential expression analysis in PBMCs from NPR patients during acute (NPR-acute) and convalescent phase (NPR-conv) as compared to healthy non-pregnant controls (NPR-control). (A) Venn diagram displaying numbers of differentially expressed up-regulated genes in PBMCs from NPR-acute and NPR-conv patients as compared to healthy non-pregnant controls (NPR-Control). The green and orange circle represents DEGs in NPR-conv and NPR-acute patients and shadow of corresponding colors represent overlapping genes between the two groups. (B) DAVID was used to identify over-represented Gene Ontology (GO) terms. Significant enriched GO terms of differentially expressed up-regulated genes in NPR-acute and NPR-conv patients. The vertical axis is the GO category and the horizontal axis is–log P value <0.05. (C) The enriched KEGG pathways of differentially expressed up-regulated genes in NPR-acute and NPR-conv patients (P value <0.05). (P value is modified Fisher’s Exact test P- value or EASE score).

Pairwise comparison of the acute and convalescent NPR patients was done with respect to the healthy non-pregnant controls. This comparison identified 679 DEGs common to both the groups while 35 and 1081 gene transcripts were unique to acute and convalescent phases respectively suggestive of extensive biological activity during convalescence (Fig 2A).

#### Gene ontology terms and pathways associated with upregulated genes in the NPR patients

To identify which biologic processes were over-represented in the DEGs, we performed gene ontology (GO) analysis at a false discovery rate of 5%. The most significant first 17 GO terms associated with the acute phase were related to biosynthesis and metabolism functions, while immune system / immune response was within the first 5 GO terms in the convalescing patients. During both phases, the common GO terms were indicative of primary non-specific immune response: response to virus, immune response, innate immune response, and inflammatory response (Fig 2B). Mounting of robust innate as well as adaptive T cell and B cell immune response was unique to convalescence while over expression of blood coagulation was unique to the acute phase. Some KEGG pathways were significantly overrepresented during both phases of the disease, with upregulated genes after correction for multiple testing (FDR 5%), including: ribosome, systemic lupus erythematosus and oxidative phosphorylation with higher gene count in the convalescent phase (Fig 2C).The identification of systemic lupus erythematosus pathway is probably on account of the overlap with the complement pathways and the large number of histone genes that were upregulated. Unique immune response pathways upregulated during convalescence included: antigen processing and presentation, cell adhesion molecules (CAMs), proteasome, spliceosome graft-versus-host disease, allograft rejection hematopoietic cell lineage and intestinal immune network for IgA production that correlates with enteric infection with HEV.

#### Comparison of pregnant and non-pregnant hepatitis E patients

**Fig 3:**
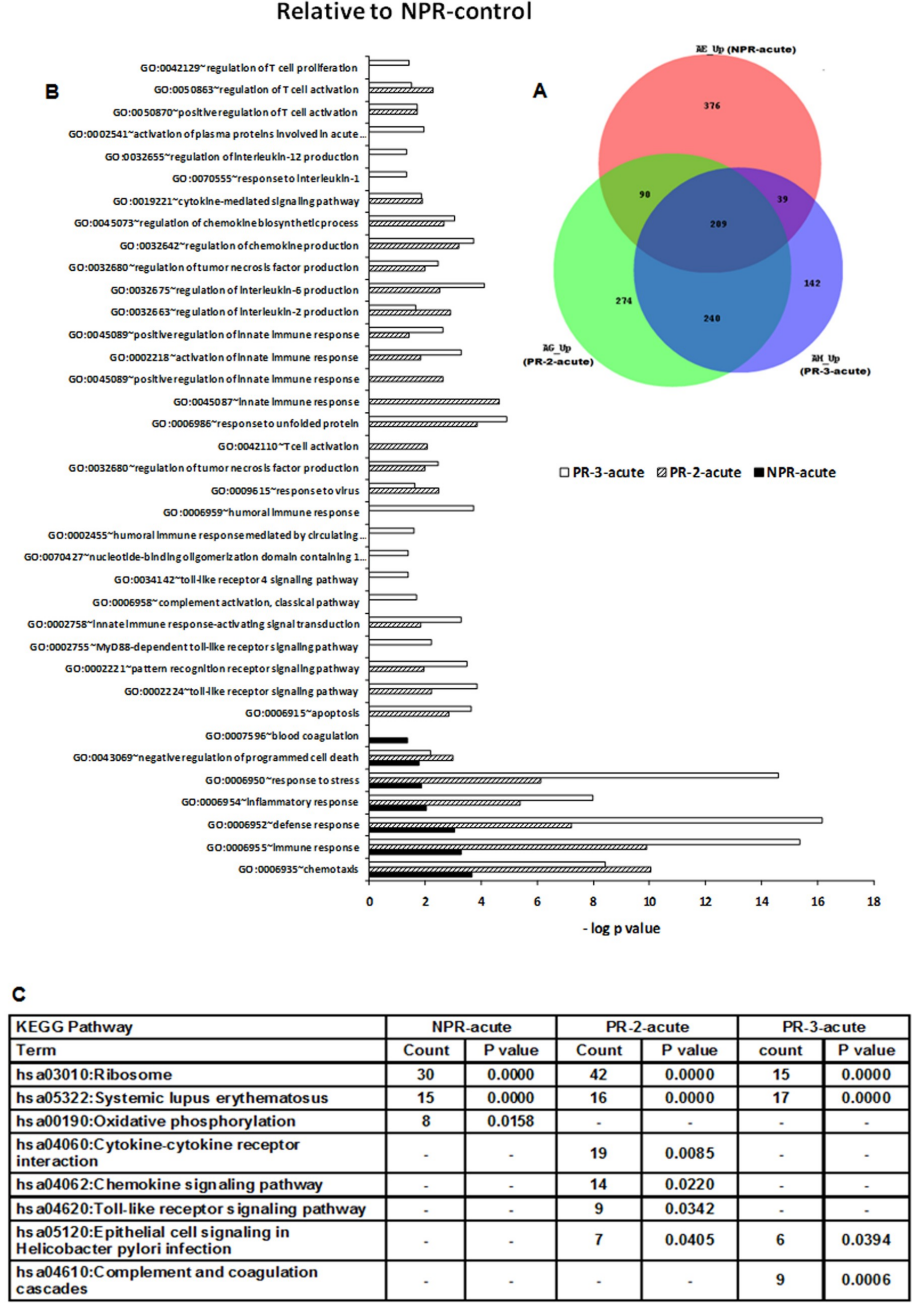
Changes in gene expression and biologic pathways affected following HEV infection: Comparison of acute-phase, non-pregnant and pregnant patients with reference to the non-pregnant controls. Differential expression analysis in PBMCs from pregnant (PR-2-acute and PR-3-acute) and non-pregnant (NPR-acute) clinical patients as compared to healthy non-pregnant controls (NPR-control). (A) Venn diagram showing the number of genes uniquely expressed by NPR-acute (orange), PR-2-acute (green) and PR-3-acute (violet) patients as compared to healthy non-pregnant controls (NPR-control) and shadows of corresponding colors denote genes commonly expressed in the respective patient groups. (B) DAVID was used to identify over-represented Gene Ontology (GO) terms. Significant enriched GO terms of differentially expressed up-regulated genes in PR-2-acute, PR-3-acute and NPR-acute patients. The vertical axis is the GO category and the horizontal axis is–log P value <0.05.(C) The enriched KEGG pathways of differentially expressed up-regulated genes in PR-2-acute, PR-3-acute and NPR-acute patients (P value <0.05). (P value is modified Fisher’s Exact test P- value or EASE score).

**Fig 4:**
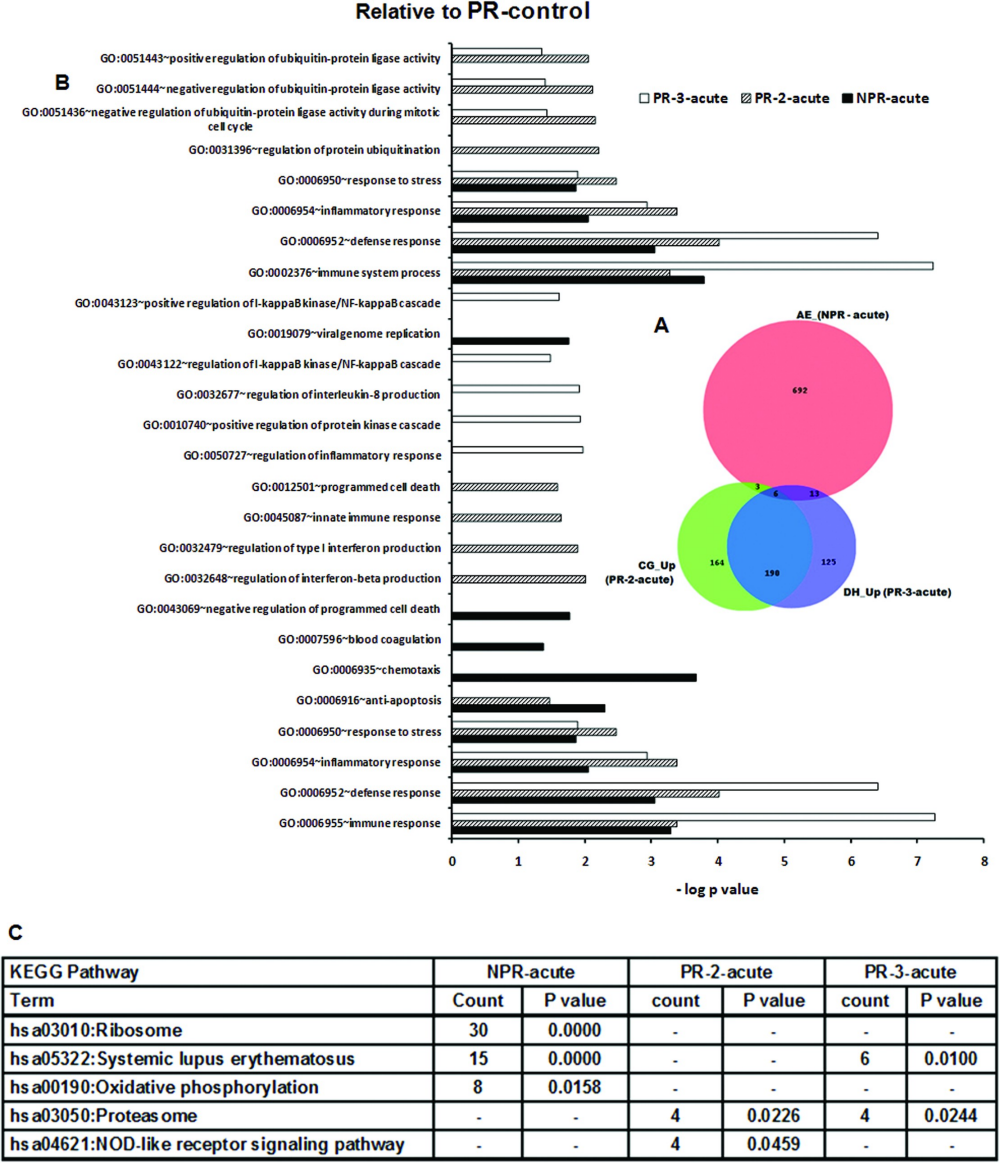
Changes in gene expression and biologic pathways affected following HEV infection: Comparison of acute-phase, non-pregnant and pregnant patients with reference to the pregnant controls from corresponding trimesters. Differential expression analysis in PBMCs from pregnant (PR-2-acute and PR-3-acute) acute patients as compared to respective healthy pregnant controls and non-pregnant acute patients (NPR-acute) as compared to healthy non-pregnant controls (NPR-control). (A) Venn diagram showing the number of genes uniquely expressed by NPR-acute (orange), PR-2-acute (green) and PR-3-acute (violet) patients as compared to respective healthy pregnant controls (PR-control) and shadows of corresponding colors denote genes commonly expressed in the respective patient groups. (B) DAVID was used to identify over-represented Gene Ontology (GO) terms. Significant enriched GO terms of differentially expressed up-regulated genes in PR-2-acute, PR-3-acute and NPR-acute patients. The vertical axis is the GO category and the horizontal axis is–log P value <0.05. (C) The enriched KEGG pathways of differentially expressed up-regulated genes in PR-2-acute, PR-3-acute and NPR-acute patients (P value <0.05). (P value is modified Fisher’s Exact test P- value or EASE score).

Pregnancy is known to modulate immune response to accommodate fetus, pregnancy duration being an important factor. To differentiate the effect of pregnancy and the virus or a possible interaction of both, pregnant patients were compared with NPR controls (Fig 3A) and healthy pregnant women in corresponding trimesters (Fig 4A). Of the 1260 DEGs recorded in all the acute-phase patients, 209 were overlapping suggestive of common biologic mechanisms operative during HEV infection while 240 were unique to the disease during pregnancy. Interestingly, the pattern of unique genes was NPR>PR-2>PR-3. When pregnancy associated modulations were taken into consideration, the number of overlapping genes reduced to 6 while 190 were common to the PR category.

#### Differences in host gene transcript abundance between acute HEV infection in the NPR and PR patients when compared to the NPR controls

There were large differences between the acute samples taken from NPR and PR patients relative to NPR controls (Fig 3B). GO terms associated with the initial non-specific immune response such as chemotaxis, response to wounding and defense response were common to all the patient categories. Involvement of innate response in the PR patients was evident by enrichment of GO terms such as toll-like receptor signaling pathway, pattern recognition receptor signaling pathway, innate immune response-activating signal transduction, activation and positive regulation of innate immune response. Uniquely, patients in the third trimester enriched MyD88-dependent toll-like receptor signaling pathway, toll-like receptor 4 signaling pathway and nucleotide-binding oligomerization domain containing 1 signaling pathway.

GO terms specific to proinflammatory cytokines (IL2, IL6, TNF), cytokine-mediated signaling pathway and regulation of chemokine production / positive regulation of chemokine biosynthetic process were common to both the PR patient groups while IL1(inflammatory) and IL12 (Th1) were unique to PR-3-acute. Both PR patient categories enriched GO terms associated with T cell responses. The PR-3-acute patients uniquely overexpressed GO terms such as complement activation classical pathway, humoral immune response, humoral immune response mediated by circulating immunoglobulin, activation of plasma proteins involved in acute inflammatory response. Overall, during acute phase, expression of immune response genes was higher and broader in the PR patients than the NPR patients exhibiting exclusive expression of non-specific immune response genes.

Of the pathways enriched (Fig 3C), ribosome and systemic lupus erythematosus were common to all the patient categories. Unique group-specific pathways included: oxidative phosphorylation (NPR-acute), Cytokine-cytokine receptor interaction, Chemokine signaling pathway, Toll-like receptor signaling pathway (PR-2-acute) and epithelial cell signaling in helicobacter pylori infection, complement and coagulation cascades (PR-3-acute). Helicobacter pylori infection related GO term included NFKBIA, JUN, ATP6V1G1, CXCR1, ATP6V1F and IL8 genes.

#### Differences in host gene transcript abundance between acute HEV infection in the NPR and PR patients when compared to the corresponding PR controls

The patterns of DEGs in PR-2-acute (PR-3-acute) groups were: total, 509 (411); up-regulated, 363 (334) and down-regulated, 146(77) genes (Fig 4A and S2 Fig). All the patients with clinical disease exhibited common GO terms (Fig 4B) related to non-specific and innate immune response such as response to stress, defense response, inflammatory response, death and immune system process. GO terms unique to NPR patients were blood coagulation and viral genome replication whereas the PR patients exhibited type I interferon production, regulation of interferon-beta production (PR-2-acute) and regulation of interlukin-8 production, regulation of I-kappaB kinase/NF-kappaB cascade (PR-3-acute). Several GO terms related to ubiquination process were enriched only in the PR patients while none of the patient groups showed enrichment of adaptive immune response related GO terms.

Unique KEGG pathways enriched with up-regulated genes were oxidative phosphorylation pathway (NPR patients), proteasome (PR patients) and NOD like receptor signaling pathway (PR-2-acute) (Fig 4C).

#### Comparisons of pregnant hepatitis E patients presenting with acute or subclinical infections

**Fig 5:**
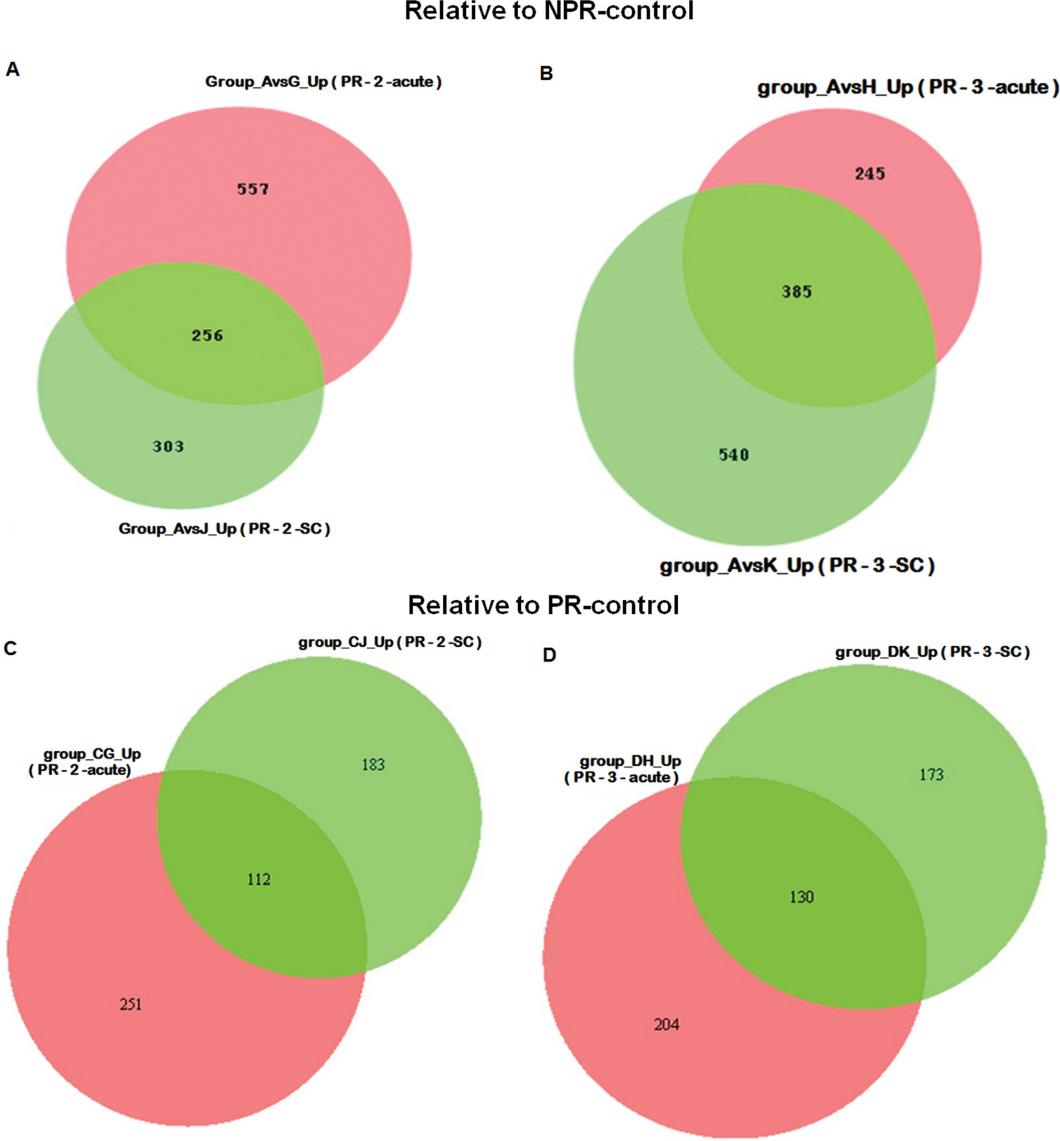
Numbers of differentially expressed genes among HEV infected pregnant women presenting with subclinical and acute infection during 2nd /3rd trimester. Venn diagram A and B, C and D displaying numbers of differentially expressed up-regulated genes as compared to healthy non-pregnant controls (NPR-control) and respective healthy trimester 2 and 3 controls (PR-2-control and PR-3-control) respectively. The green and orange circle represents DEGs in PR-2-SC/ PR-3-SC and PR-2-acute/PR-3-acute patients and shadow of corresponding colors represent overlapping genes between the groups respectively.

Our next aim was to understand differences in the biologic processes between acute and subclinical HEV infections in the later trimesters (Fig 5). By comparing subclinical and acute HEV infections during the 2^nd^ trimester relative to the NPR controls / PR controls, we identified 256/112 (common to both groups), 303/183 (unique to subclinical infection) and 557/251 (unique to acute disease) genes. Respective gene expression during the third trimester was 385/130, 245/204 and 540/173.

#### Differences in host gene transcript abundance between acute and subclinical HEV infections in the pregnant women when compared to the NPR controls

**2^nd^ trimester**

**Fig 6:**
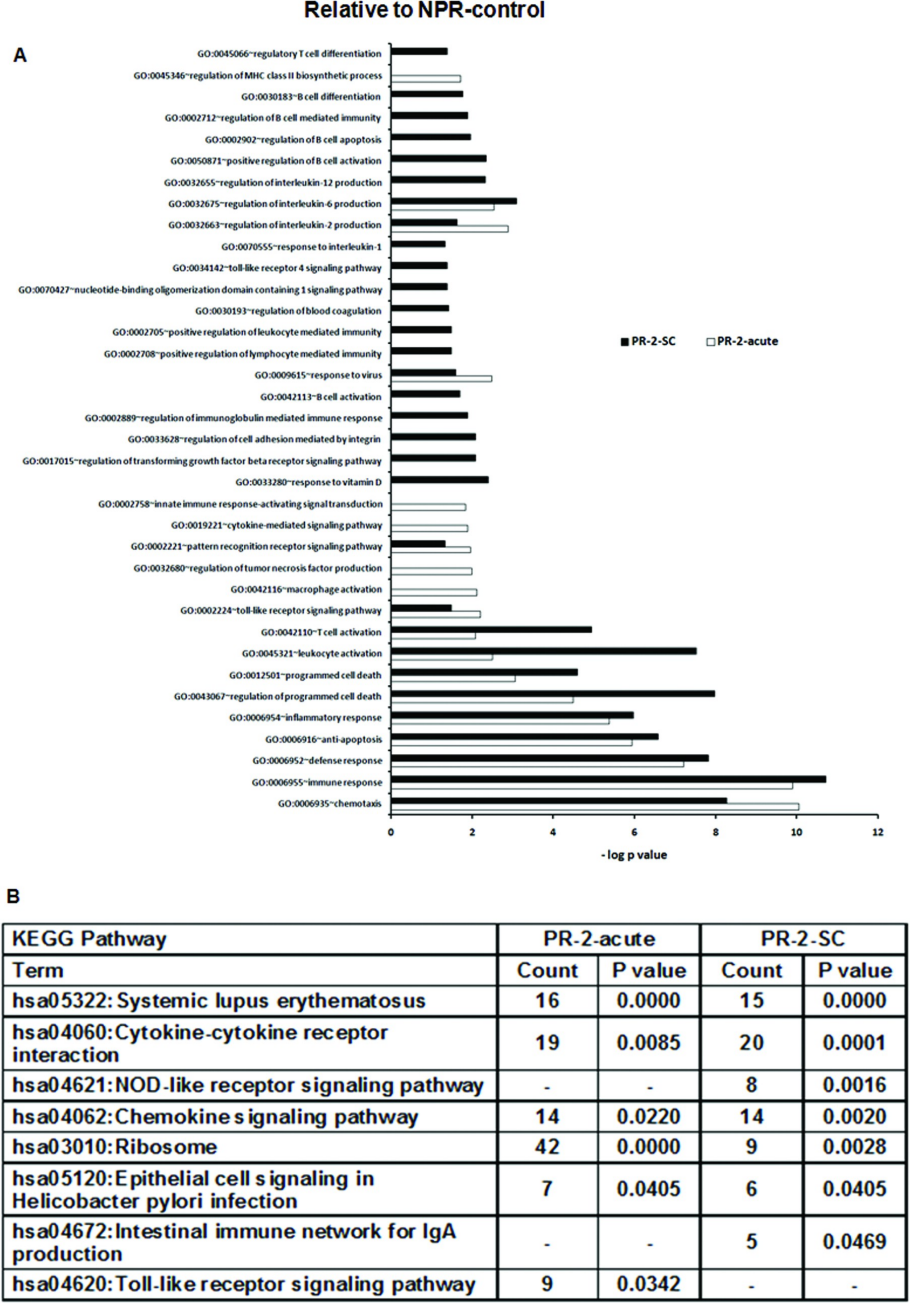
Changes in gene expression and biologic pathways affected following subclinical or acute HEV infection in the pregnant women in 2nd trimester with reference to the non-pregnant controls. Differential expression analysis in PBMCs from pregnant subclinical (PR-2-SC) and acute (PR-2-acute) patients as compared to healthy non-pregnant controls (NPR-control). (A) DAVID was used to identify over-represented Gene Ontology (GO) terms. Significant enriched GO terms of differentially expressed up-regulated genes in PR-2-SC and PR-2-acute patients. The vertical axis is the GO category and the horizontal axis is–log P value <0.05. (B) The enriched KEGG pathways of differentially expressed up-regulated genes in PR-2-SC and PR-2-acute patients (P value <0.05). (P value is modified Fisher’s Exact test P- value or EASE score).

During the 2^nd^ trimester, GO terms associated with early-non-specific, inflammatory and innate immune response, such as toll-like receptor signaling pathway, pattern recognition receptor signaling pathway, IL2, IL6 and T cell response were common in both subclinical and acute forms of the disease (Fig 6A).

The acute disease marked unique enrichment of regulation of tumor necrosis factor production, regulation of MHC class II biosynthetic process, innate immune response-activating signal transduction and macrophage activation. A large number of GO terms were unique to the subclinical infection. These included: response to vitamin D, IL12, response to interlukin-1,regulation of transforming growth factor beta receptor signaling pathway, NOD-1 signaling pathway, regulation of blood coagulation, positive regulation of adaptive immune response based on somatic recombination of immune receptors built from immunoglobulin superfamily domains, regulatory T cell differentiation, regulation of immunoglobulin mediated immune response and several terms related to B cell activation and functions.

When we considered the KEGG pathways (Fig 6B), Ribosome, systemic lupus erythematosus, cytokine-cytokine receptor interaction and chemokine signaling pathway were common to both forms of HEV infection. Association of NOD-like receptor signaling pathway and Intestinal immune network for IgA production was unique in subclinical infection and that of toll-like receptor signaling pathway was restricted to acute infection.

**3^rd^ trimester**

**Fig 7:**
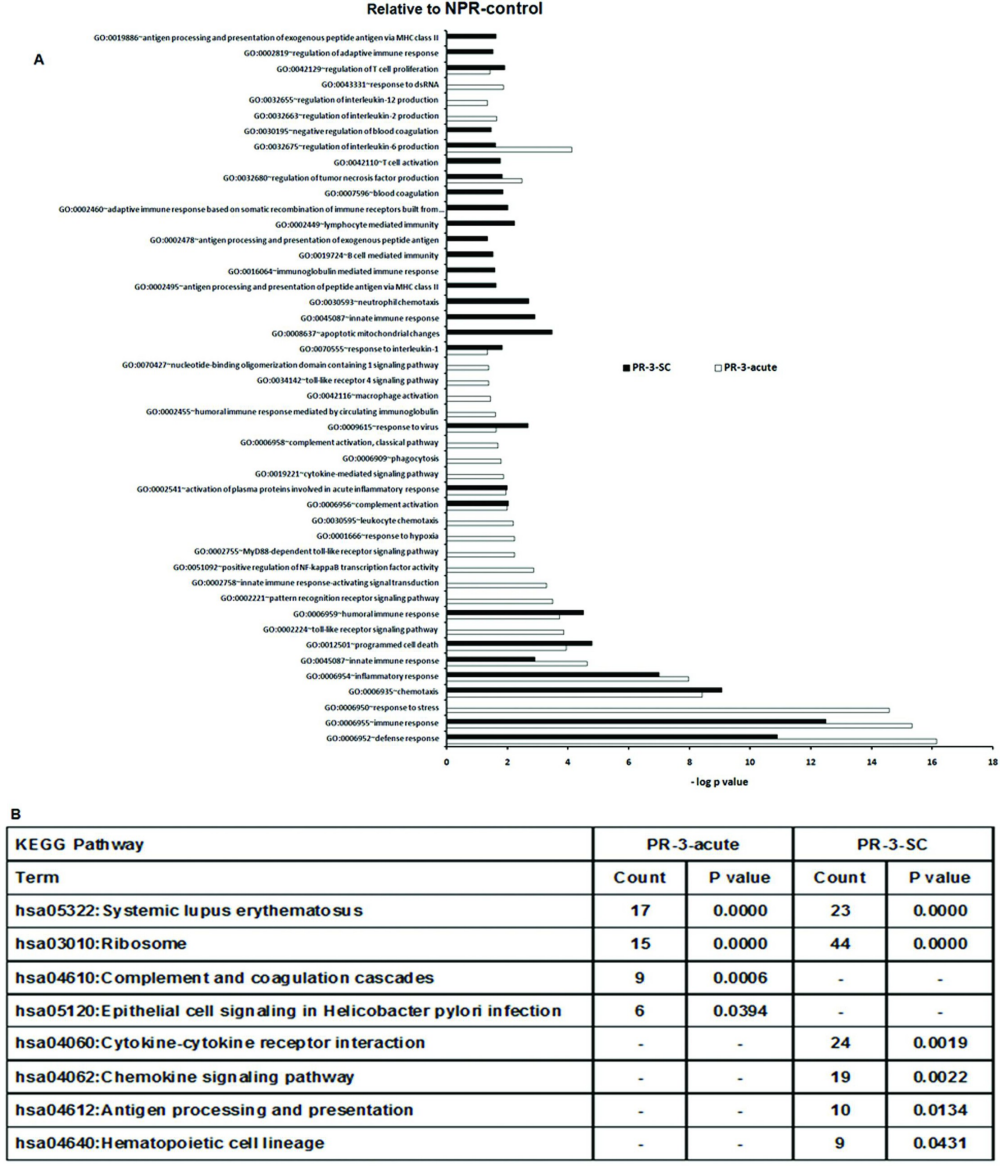
Changes in gene expression and biologic pathways affected following subclinical or acute HEV infection in the pregnant women in 3rd trimester with reference to non-pregnant controls. Differential expression analysis in PBMCs from pregnant subclinical (PR-3-SC) and acute (PR-3-acute) patients as compared to healthy non-pregnant controls (NPR-control). (A) DAVID was used to identify over-represented Gene Ontology (GO) terms. Significant enriched GO terms of differentially expressed up-regulated genes in PR-3-SC and PR-3-acute patients. The vertical axis is the GO category and the horizontal axis is–log P value <0.05.(B)The enriched KEGG pathways of differentially expressed up-regulated genes in PR-3-SC and PR-3-acute patients (P value <0.05).(P value is modified Fisher’s Exact test P- value or EASE score).

During the 3^rd^ trimester, broad inflammatory pathways were operative in both infection types. The subclinical infection exhibited robust adaptive immune response than acute disease as evident by several over-represented GO terms (Fig 7A).

The early, non-specific immune response was common to both infection types. The GO terms common to both acute and subclinical HEV infections included: response to virus related, complement activation, activation of plasma proteins involved in acute inflammatory response, IL1, regulation of IL6 production and regulation of TNF production. GO terms such as blood coagulation, coagulation, negative regulation of coagulation were enriched uniquely in the subclinical category.

As far as the adaptive response is considered, GO terms associated with T cell activation, proliferation, were common to both subclinical and acute presentations. However, active T cell response was seen uniquely in the subclinical category (antigen processing and presentation, adaptive immune response based on somatic recombination of immune receptors built from immunoglobulin superfamily domains, regulation of adaptive immune response, antigen processing and presentation of peptide or polysaccharide antigen via MHC class II, antigen processing and presentation of peptide antigen via MHC class II, antigen processing and presentation of exogenous peptide antigen via MHC class II). Humoral immune response related GO terms such as humoral immune response was common to both, while B cell mediated immunity (C1QB, HLA-DRA, C1QC, FCERIG, CIQA, CLU) was uniquely in subclinical infection.

We could distinguish acute and subclinical infections in the 3^rd^ trimester by the unique presence of immunity-related KEGG pathways such as cytokine-cytokine receptor interaction, antigen processing and presentation and hematopoietic cell lineage during subclinical infection and complement and coagulation cascades, epithelial cell signaling in Helicobacter pylori infection (NFKBIA, JUN, ATP6V1G1,CXCR1,ATP6V1F, IL8 genes) in the acute disease (Fig 7B).

#### Differences in host gene transcript abundance between acute and subclinical HEV infections in the pregnant women when compared to the respective pregnant controls

**2^nd^ trimester**

**Fig 8:**
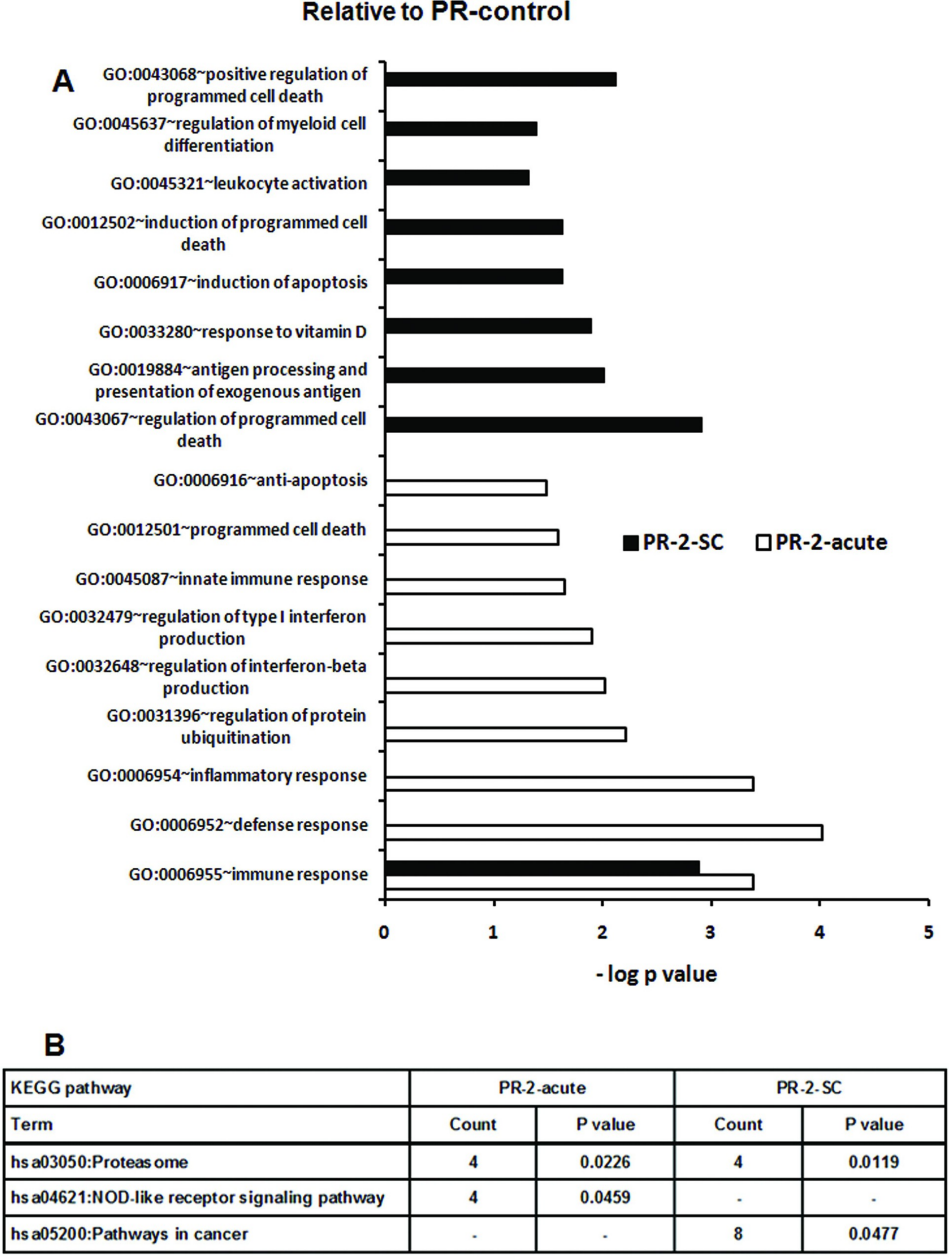
Changes in gene expression and biologic pathways affected following subclinical or acute HEV infection in the pregnant women in 2nd trimester with reference to pregnant controls from corresponding trimesters. Differential expression analysis in PBMCs from pregnant subclinical (PR-2-SC) and acute (PR-2-acute) patients as compared to respective healthy pregnant controls. (A) DAVID was used to identify over-represented Gene Ontology (GO) terms. Significant enriched GO terms of differentially expressed up-regulated genes in PR-2-SC and PR-2-acute patients. The vertical axis is the GO category and the horizontal axis is–log P value <0.05. (B)The enriched KEGG pathways of differentially expressed up-regulated genes in PR-2-SC and PR-2-acute patients (P value <0.05).(P value is modified Fisher’s Exact test P- value or EASE score).

The acute and subclinical HEV infections were characterized by no over-representations of GO terms associated with early non-specific response in the subclinical category and adaptive immune response in the clinical group respectively. GO terms related to interferons such as regulation of interferon-beta production, regulation of type I interferon production were unique in the clinical category while response to vitamin D, regulation of myeloid cell differentiation, leukocyte activation were seen in the subclinical category (Fig 8A).

**3^rd^ trimester**

**Fig 9:**
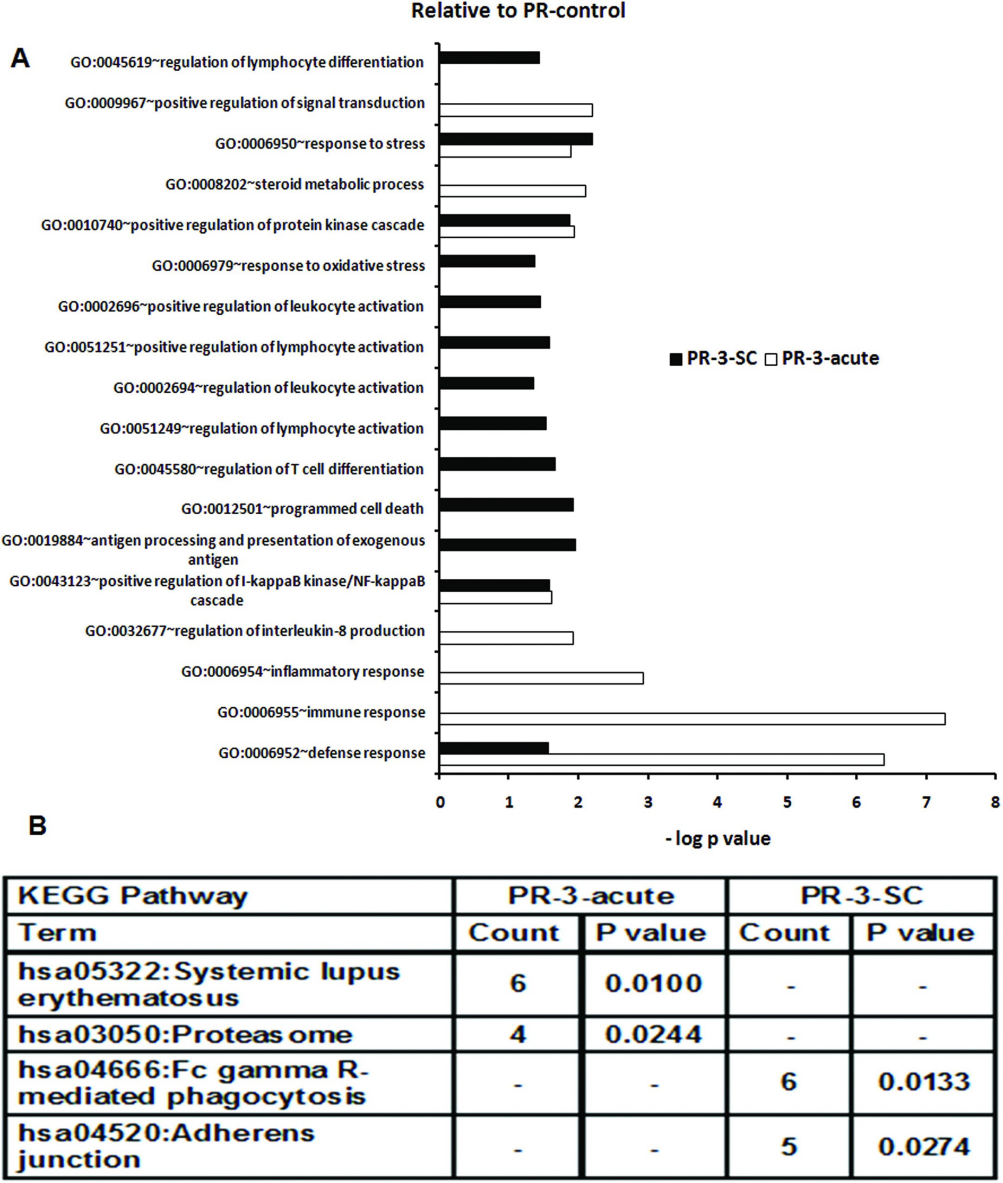
Changes in gene expression and biologic pathways affected following subclinical or acute HEV infection in the pregnant women with reference to pregnant controls from corresponding trimesters. Differential expression analysis in PBMCs from pregnant subclinical (PR-3-SC) and acute (PR-3-acute) patients as compared to respective healthy pregnant controls. (A) DAVID was used to identify over-represented Gene Ontology (GO) terms. Significant enriched GO terms of differentially expressed up-regulated genes in PR-3-SC and PR-3-acute patients. The vertical axis is the GO category and the horizontal axis is–log P value <0.05. (B)The enriched KEGG pathways of differentially expressed up-regulated genes in PR-3-SC and PR-3-acute patients (P value <0.05).(P value is modified Fisher’s Exact test P- value or EASE score).

In addition to GO terms associated with response to stress and defense seen in the subclinical infection, acute disease over-represented unique GO terms such as positive regulation of signal transduction, steroid metabolic process, inflammatory response, positive regulation of protein kinase cascade and regulation of IL8 production. In subclinical infection, unique GO terms such as positive regulation of lymphocyte activation, regulation of lymphocyte activation, positive regulation of leukocyte activation, regulation of lymphocyte differentiation, response to oxidative stress and regulation of leukocyte activation were overexpressed (Fig 9A).

Similar to 2^nd^ trimester, the acute category did not over-represent adaptive immune response GO terms whereas antigen processing and presentation of exogenous antigen (common to 2^nd^ and 3^rd^ trimesters) and regulation of T cell differentiation were restricted to the subclinical infections.

As far as the KEGG pathways are concerned (Fig 8B), proteasome was enriched in both the patient groups in the 2^nd^ trimester while acute and subclinical infections respectively were associated with NOD-like receptor signaling pathway and pathways in cancer (VHL, MMP9, PIAS4, PTGS2, DVL2, SMAD4, SPI1, CRKL genes). In the third trimester, systemic lupus erythematosus and proteasome were enriched in the acute disease while Fc gamma R-mediated phagocytosis (VAV1, MARCKSL1, WASF2, DNM2, HCK, MAP2K1 genes) and adherens junction (ACTG1, WASF2, SMAD4, CSNK2B, EP300 genes) represented the subclinical category (Fig 9B).

#### GO terms and pathways associated with down-regulated genes

**Fig 10:**
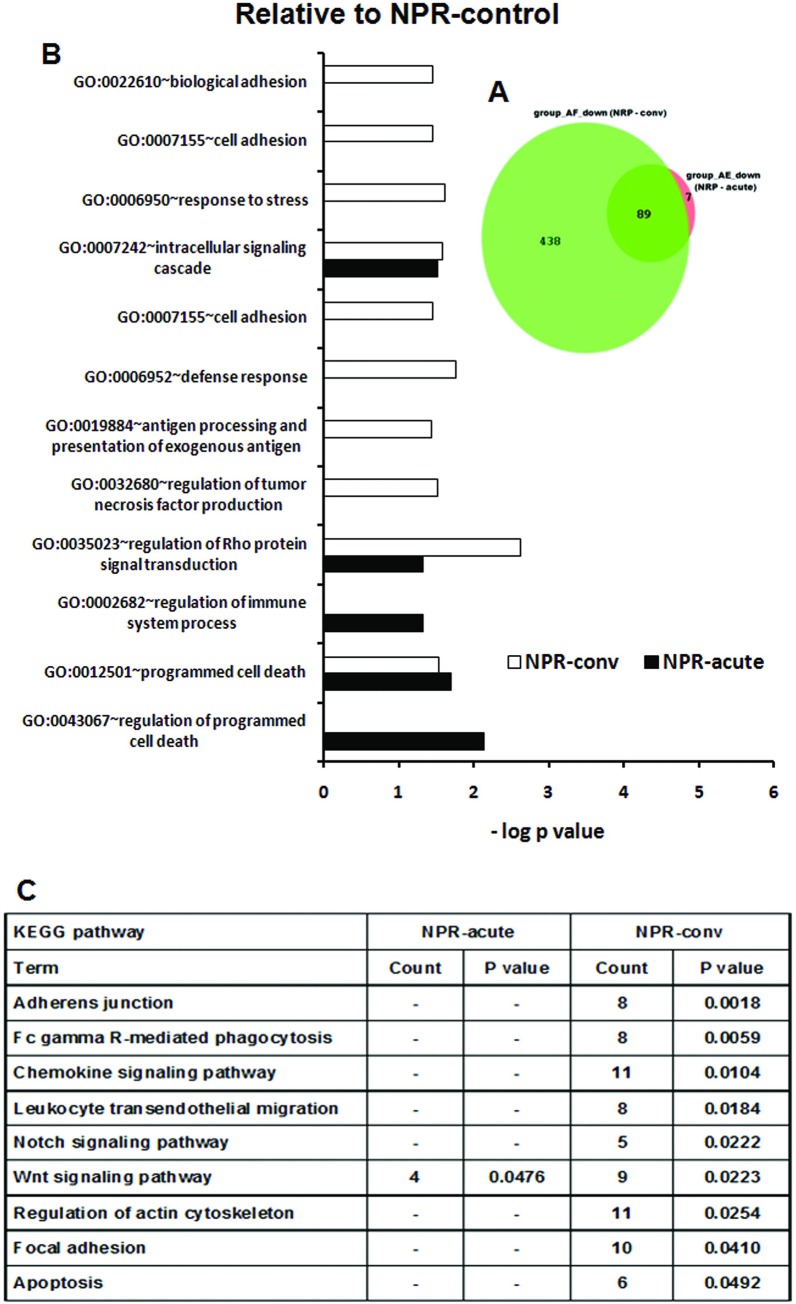
Changes in gene expression and biologic pathways affected following HEV infection in acute or convalescent patients with reference to non-pregnant controls. Differential expression analysis in PBMCs from NPR patients during acute (NPR-acute) and convalescent phase (NPR-conv) as compared to healthy non-pregnant controls (NPR-control). (A) Venn diagram displaying numbers of differentially expressed down-regulated genes in PBMCs from NPR-acute and NPR-conv HEV infected patients as compared to healthy non-pregnant controls (NPR-control). The green and orange circle represents DEGs in NPR-conv and NPR-acute patients and shadow of corresponding colors represent overlapping genes between the two groups. DAVID was used to identify over-represented Gene Ontology (GO) terms. (B) Significant enriched GO terms of differentially expressed down-regulated genes in NPR-acute and NPR-conv patients. The vertical axis is the GO category and the horizontal axis is–log P value <0.05. (C) The enriched KEGG pathways of differentially expressed down-regulated genes in NPR-acute and NPR-conv patients (P value <0.05). (P value is modified Fisher’s Exact test P- value or EASE score).

As evident from Fig 10B, GO terms enriched with down-regulated genes were related to apoptosis (all NPR patients) and to non-specific immune response, antigen processing and presentation of exogenous antigen (convalescent phase). S2 Fig displays no overlap among NPR and PR patients.

**Fig 11:**
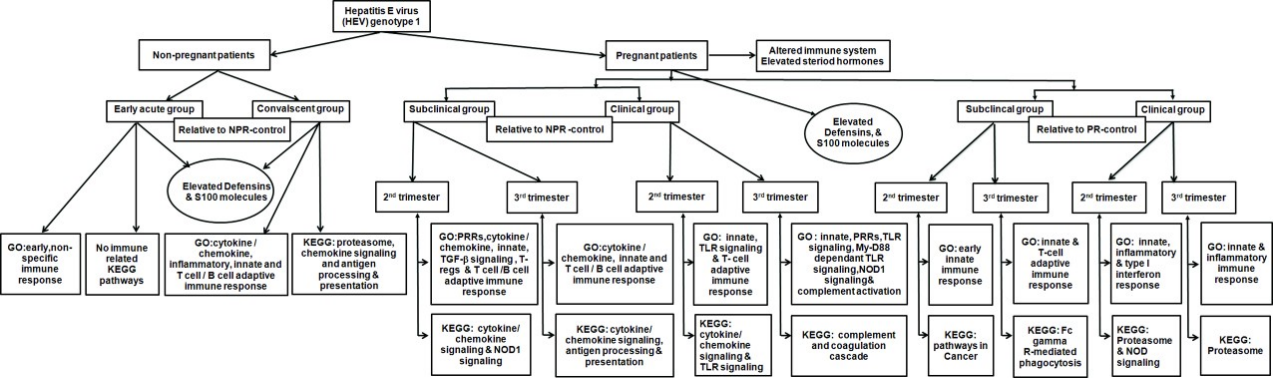
Summary of transcriptome modulation by HEV and pregnancy (duration). Abbreviations: GO, Gene Ontology terms; PRRS, pattern recognition receptors;TGF-β, transforming growth factor-beta; NOD, nucleotide-binding oligomerization domain; TLR, toll- like receptor; My-D88, myeloid differentiation primary response 88; T-regs, regulatory T-cells.

Fig 11 depicts summary of major GO terms and KEGG pathways differentially modulated by HEV infection with or without pregnancy.

#### Validation of RNAseq data

**Fig 12:**
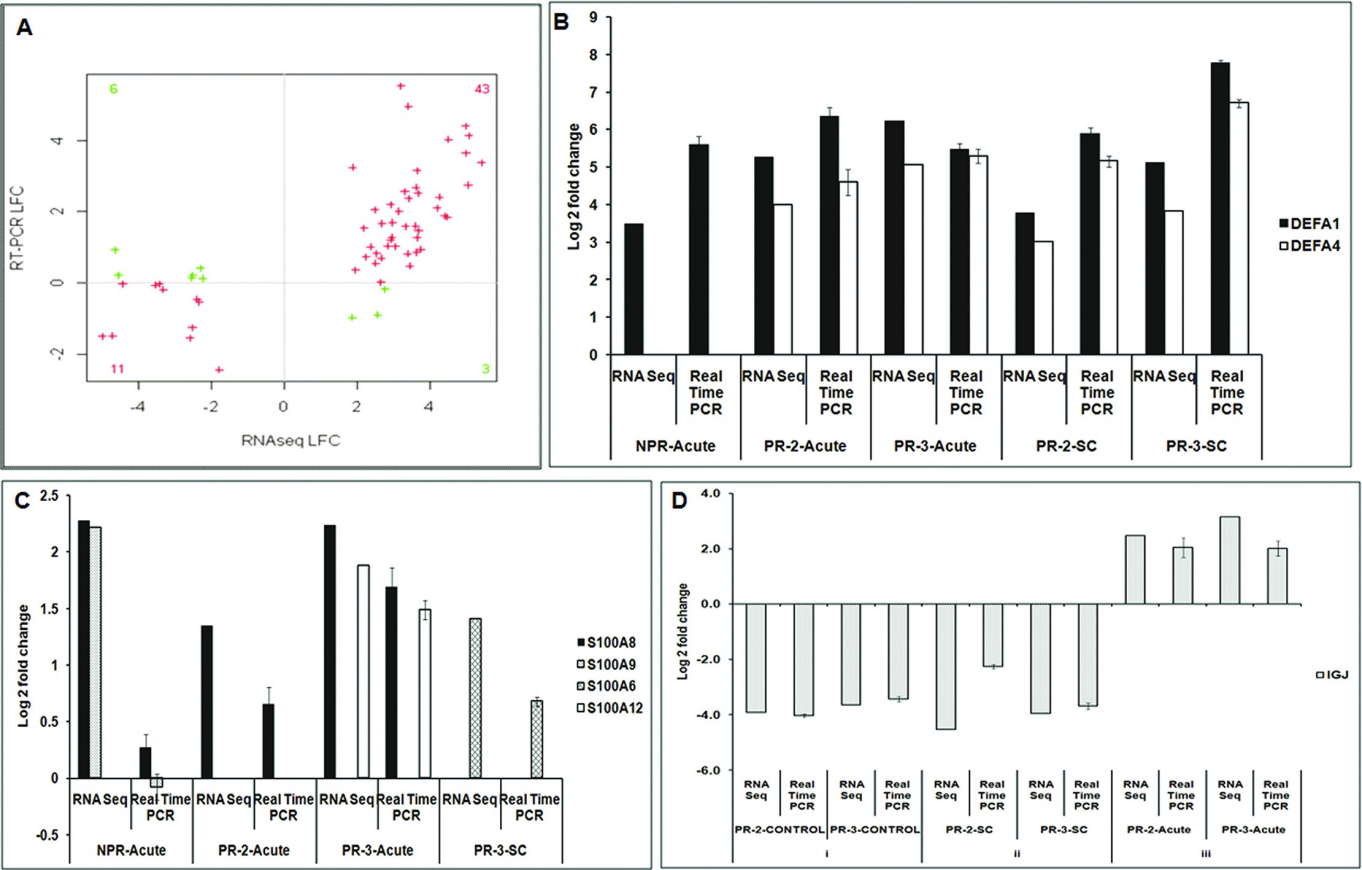
**A** Validation of RNA-Seq data. Correlation plot of log2 transformed values of expression of genes in RNA-Seq and RT-PCR. Spearman rank Correlation coefficient value is 0.7788, P value 2.20E-16. The plus signs in the plot indicates genes with similar expression pattern (red) and genes with different expression pattern (green) in RNA-Seq and RT-PCR. Samples from healthy pregnant all trimesters, acute and convalescent phase non-pregnant patients and acute later trimester pregnant patients were taken for validation of RNA-Seq data. **Fig 12B** Relative gene expression by SYBR green-based Real Time-PCR in different study groups. The XY plots represent normalized log fold expression of defensin genes (DEFA1 and DEFA4) estimated by Real-Time PCR and RNA Seq analysis when compared to healthy non-pregnant controls. **Fig 12C** Relative gene expression by SYBR green-based Real Time-PCR in different study groups. The XY plots represent normalized log fold expression of S100A6, S100A8, S100A9 and S100A12 genes estimated by Real-Time PCR and RNA Seq analysis, when compared to healthy non-pregnant controls in NPR-Acute, PR-2-Acute PR-3-Acute and PR-3-SC. **Fig 12D** Relative gene expression by SYBR green-based Real Time-PCR in different study groups. The XY plots represent normalized log fold expression of immunoglobulin IGJ gene estimated by Real-Time PCR and RNA Seq analysis. PR-2-SC, PR-3-SC, PR-2-CONTROL and PR-3-CONTROL groups were compared with healthy non-pregnant controls while PR-2-Acute, PR-3-Acute groups are compared with respective healthy trimester controls using Taqman assay.

Validation of RNA-seq data using TaqMan-based quantitative RT-PCR showed excellent correlation (r^2^ = 0.78,p- value 2.20E-16) suggestive of utility of RNA-seq in identifying targets that can be examined during subsequent studies employing appropriate experimental protocols (**Fig 12A**). Similar expression patterns of IgJ, DEFA4 and IFNγ genes by both the assays confirm our observations with RNAseq data. Expression of CD37, PLAGL2 and TICAM genes did not correlate while that of PF4 and WDTC1 genes correlated in 1/3^rd^ of the samples tested. A complete correlation was seen for the remaining genes.

#### Validation of modulation of defensin, S100 and Ig genes

In view of the association of defensins and S100 genes as well as Ig genes in disease presentation during pregnancy, we quantitated these genes using SYBR green-based quantitative real time PCR (Fig 12B–12D). By both the assays, DEFA4 was not detected in the NPR-acute category while DEFA1 and DEFA4 genes were upregulated in the pregnant patients during acute and subclinical infections (Fig 12B). Except for the NPR patients, similar correlation between the two assays was seen with S100 genes (Fig 12C). We could use a single gene (IGJ) for Ig gene expression validation. Similar expression profile was seen in pregnant controls (both trimesters) and corresponding subclinical infections (Fig 12D). Additionally, raised expression of IGJ gene recorded by RNA-seq in the PR-2-CL and PR-3-CL patients when compared with the trimester-matched healthy controls was confirmed by TaqMan-RT-PCR. These results reveal reproducibility of RNA-seq results by both TaqMan and SYBR green-based real time PCR assays and ascertain validity of the conclusions drawn that need to be further confirmed by cell-based specific assays.

#### Individual genes of interest

Next, we would like to address differential expression of some of the immune response modulating genes in different patient groups. The unbiased profiling approach revealed that out of a very large number of immune related genes (S3–S14 Tables), only a few were modulated, though differently, in different groups of hepatitis E patients.

#### CD1D and GZMM

We report higher expression of CD1D mRNA in both PR and NPR self-recovering patients in the early acute phase. The raised mRNA transcripts of CD1D and GZMM (and CD69) in the healthy pregnant women suggest increased circulating NK/NKT cells as a part of pregnancy-associated immunomodulation. HEV infection of these women led to the downregulation of these genes, the levels being comparable to NPR patients. It appears that though self-recovering, the PR and NPR patients behave differently with respect to these cells. When we compared clinical and subclinical HEV infections, an upregulation was seen during subclinical infection in the 3^rd^ trimester while no change was recorded in the 2^nd^ trimester.

#### Type-1 interferons

Dendritic cells (DCs) play a crucial role in linking innate and adaptive immune response and can detect cytokines produced by the infected cells, type-I interferons being most important. It is therefore surprising that despite identifying several upregulated IFN-inducible genes, we did not detect raised expression of IFNα or IFNβ in all the patient groups studied. At protein level, we detected reduced circulating IFNα levels in these patients while IFNβ was not tested [40].

#### Interferon-gamma

HEV infection in the pregnant or non-pregnant patients did not lead to induction of IFNγ gene while 2.99 fold rise was seen during convalescence. The higher mRNA transcripts in the pregnant patients were associated with the pregnancy status.

#### Antimicrobial peptides

Defensins represent an important group of antimicrobial peptides in humans. This study showed the involvement of alpha defensins (DEFA) in hepatitis E. In both clinical and subclinical categories, DEFA1 gene was upregulated (NPR 3.5fold; PR-2-acute/SC, 5.3fold/3.8fold and PR-3-acute/SC, 6.3fold/5.1fold) suggesting protective role in both PR and NPR patients. In addition, raised gene expression of DEFA1B, DEFA3 and DEFA4 was recorded in the PR patients with clinical or subclinical HEV infection. When compared to the PR controls, DEFA4 was raised in both the clinical PR categories, DEFA5 was 3.7 fold down regulated in PR-2-acute while no change was noted in PR-3-acute group.

An important observation is differential expression of transcripts of S100 series molecules. Members of this family are small, acidic calcium binding proteins and there is increasing evidence showing that these proteins also act as “danger associated molecular patterns”. These are endogenous ligands of TLR4 [41] and RAGE [41]. An increased transcription of S100A8, S100A9, S100A11 genes was observed in the NPR patients. Higher expression of S100A8 during acute hepatitis in both trimesters and additionally S100A12 in the third trimester, with normal expression of S100A9 suggests similar role in the PR patients as well. A further comparison among acute and subclinical infections with respect to the PR controls showed upregulation of different molecules of S100 series in the acute disease while subclinical infections exhibited either downregulated or normal levels.

#### Immunoglobulin genes

Immunoglobulin genes deserve a special focus. In humans, there is a potential to generate 8262 heavy chain genes and 320 light-chain genes as a result of variable region gene rearrangements. Therefore, the potential number of heavy and light-chain combinations is very high (~million combinations). In this context, the number of genes upregulated in the NPR patients was 28 while none and 1 gene exhibited higher levels in the PR patients. In the control PR women, these genes were downregulated (14 in PR-2-control and 16 in PR-3-control). When compared to the PR controls, 26 and 48 Ig mRNA transcript levels were elevated in PR-2-acute and PR-3-acute categories respectively documenting HEV induced expression of Ig genes in the NPR and PR patients.

A different scenario was noticed in the subclinical infection. When compared to control PR women in 2^nd^/3^rd^ trimesters, 6 IgG genes in the PR-2-SC and 5 Ig genes in the PR-3-SC categories were upregulated. Thus, the number of Ig genes expressed was proportional to the disease severity.

#### TNF

TNF-*α* and TNF-*β* have antiviral activity and synergize with interferons in the induction of resistance to both RNA and DNA virus infections [42]. The production of TNFs is induced by viruses; virus-infected cells are selectively killed by TNFs and this activity is accelerated by IFN-γ. The present study revealed that induction of TNF gene was associated with subclinical HEV infection in both the trimesters (4.8 fold and 4.2 fold respectively). During the second trimester, 1.7fold increase was seen in healthy controls and acute disease whereas no change was recorded during the 3^rd^ trimester.

## Discussion

This study for the first time provides comparative transcriptome analysis of PBMCs obtained from hepatitis E patients and addresses impact of pregnancy. Next generation sequencing technology has proved to be a powerful tool providing comparative mRNA transcript levels of a very large number of genes from a clinical sample in a quantity that can be easily collected from a patient. However, as post-transcriptional modifications are important in determining functional outcome, such studies are generally used as useful pointers to identify target genes / pathways that must be validated by appropriate experiments. The data presented here highlights significant differences among clinical and subclinical infections in pregnant women that need to be extended to fulminant disease and confirmed by well-defined immunologic assays.

We first defined characteristic transcriptome profiles among the non-pregnant patients during early-acute and convalescent disease phases. The acute phase was characterized by early, non-specific immune response whereas during convalescence, cytokine/chemokine, inflammatory, innate and T /B cell adaptive immune response was seen (Fig 2). The results revealed that at transcriptional level, a moderate immune response was generated in the NPR patients. Based on the microarray analysis of sequential liver tissues, Yu et al [43] concluded generation of attenuated immune response in chimpanzees infected with HEV when compared to HCV-infected chimpanzees. The similarity of observations in the liver (chimp study) and PBMCs (human, present study) is noteworthy and adds special significance to the PBMC-based data.

Second, we sought to identify features of the whole-blood transcriptome that were unique to the disease in pregnancy. For this, later trimesters (2^nd^ and 3^rd^) known to be associated with high mortality were considered separately and compared with non-pregnant and healthy pregnant controls in the corresponding trimesters. The results clearly showed that the responses during 2^nd^ and 3^rd^ trimesters were distinct and these categories should not be pooled together for fine immunologic analyses. In contrast to exclusive expression of nonspecific immune response genes in the NPR patients, the PR patients exhibited broader and heightened expression of genes associated with innate as well as adaptive T and B cell responses. In fact, the acute-phase PR patients induced transcription of several additional adaptive-immunity-associated pathways than even the convalescing NPR patients.

Pathogen recognition is an important step in the activation of inflammatory pathways. Of the three types of pattern recognition receptors, our data shows possible involvement of TLRs and NLRs in HEV infection. Of note, we earlier reported temporal activation of TLR4/7/8 (protein and gene levels) and TLR3 (gene) in the NPR patients that was reduced in the PR patients. This reduction actually reflected pregnancy status and not HEV infection [26]. The overexpression of toll-like receptor signaling pathway, TLR-4 signaling pathway and pattern recognition receptor signaling pathway suggests role of TLR4 in the PR patients. Additionally, patients in the third trimester enriched MyD88-dependent toll-like receptor signaling pathway. In the light of these findings, we would like to point out that expression of TLR2 / TLR3 /TLR4 genes was increased in acute hepatitis E patients while a significant reduction was evident in the fulminant category [27]. Another important study by Sehgal et al [28] documented that the expression of TLR3 and TLR9 and downstream MYD88 signaling molecules IRF3 and IRF7 were significantly down regulated in pregnant women with FHF-E than with acute hepatitis E.

Taken together, TLR3/4/9 seem to play crucial role in modulating disease severity during pregnancy and deserve further in-depth studies in relation to prognosis as well as therapy.

Importantly, for the first time, possible involvement of NOD1 signaling pathway in acute disease during the third trimester is shown. Association of NOD1 with subclinical HEV infection during 2^nd^ trimester was pregnancy driven. Though NOD1 and NOD2 members of the NLR family are activated by specific bacterial peptides [44], similar to dengue [45] and RSV [46], we report HEV-induced NLR activation. In the light of increased expression of NOD1 in hepatitis C patients [47] and the involvement of NOD1 through interaction with dsRNA in hepatocytes infected in-vitro or in-vivo with HCV [48], current observations with PBMCs need to be extended to hepatocytes.

Our next aim was to examine if we can identify transcripts specific to subclinical HEV infection in pregnant women that otherwise constitute high-risk category for severe liver disease. We could identify several GO terms specific to subclinical infection when compared to NPR controls. However, several of these were related to pregnancy as evidenced by comparisons with pregnant controls. The relationship of immunoglobulin genes with disease presentation is noteworthy. The subclinical infection correlated with upregulation of lesser number of Ig genes (6 in the PR-2-SC and 5 in the PR-3-SC) than the corresponding acute disease (26 and 48 genes respectively). Further, humoral responses associated GO terms were enriched during subclinical infection during 2^nd^ trimester while no difference was seen among PR-3-SC and PR-3-acute patients. In fulminant hepatitis E, the number of antigen-specific, IgG-producing B cells increased following polyclonal stimulation of PBMCs than in the patients with uncomplicated clinical disease [49].We, on the other hand, reported (i) higher anti-HEV titres in the fulminant hepatitis patients than the self-recovering non-pregnant patients [24] and (ii) lower IgG-anti-HEV titres in the pregnant women with subclinical HEV infection than those with clinical disease [40]. Taken together, antibodies seem to play an important role in disease severity. Role of antibodies in disease severity has been reported for several viral infections such as influenza [50], SARS [51], dengue [52] and measles [53].

Of note, RNA-seq data with respect to defensins, S100 and Ig genes was further confirmed by the gene-specific real time PCR (Fig 12B–12D) strengthening the association of modulation of these genes with hepatitis E and clinical presentation. A single pregnant woman in the third trimester with fulminant hepatitis E showed downregulation of defensin and S100 genes. At recovery defensin expression increased while that of S100 remained low. This observation of lowered innate immune response needs to be confirmed with a large series.

We now would like to address two small antimicrobial peptides of innate immune system that seem to have a role in protection/pathogenesis of hepatitis E. The observed upregulation of some of the S100 series molecules transcripts during acute disease with either downregulated or normal levels during subclinical infections suggests possible association with the outcome of HEV infection. High levels of S100A4 protein in human carcinoma cells are strongly associated with their metastatic capability [54–56] S100A4 was demonstrated to be a strong indicator of a poor prognosis of human breast cancers [57–58].

Overexpression of alpha defensins in hepatitis E patients (irrespective of pregnancy and clinical presentation) suggests definite role in protection. These small, cationic peptides have broad anti-microbial activity and the ability of human α-defensins to neutralize viruses has been described. Neutralization of the enveloped viruses depends on the disruption of viral lipid envelope, steric interference with receptor binding, interaction with cellular factors, or post-entry blocks [59]. In a mouse model deficient in activated α-defensins in the small intestine, Paneth cell α-defensins could protect mice from oral infection by a pathogenic mouse adenovirus 1 virus [60]. Antiviral activity through potentiation of neutralizing antibody response adds to the possible mechanisms of alpha defensins-dependent immunity. The role of both alpha defensins and S100 series molecules need to be confirmed by functional assays and extended to fulminant hepatitis E. Response to vitamin D was uniquely associated with subclinical infection during 2^nd^ trimester. Activated vitamin D is known to act as an immune system modulator preventing excessive expression of inflammatory cytokines, increasing “oxidative burst” potential of macrophages and stimulation of the expression of potent anti-microbial peptides including defensins present in neutrophils, monocytes, NK cells and certain epithelial cells.

In an interesting study by Huang et al [61], microarray analysis of PBMCs from asymptomatic and symptomatic Influenza infections following experimental infection of the virus to human volunteers showed that activation of all known classes of PRRs led to symptomatic infection while the absence or downregulation was characteristic of asymptomatic infection. Though the differentiation of immune response during symptomatic and asymptomatic HEV infection in the present study is complicated by the pregnancy status and duration of pregnancy, a similar trend is seen when compared to non-pregnant controls. Acute disease was characterized by the activation of TLRs and additionally NOD1 signaling pathway in the 3^rd^ trimester. On the contrary, NOD-like receptor signaling was associated with subclinical infection in the 2^nd^ trimester.

The transcriptome analysis did not identify increased expression of mRNA levels of the cell-specific markers of immune cell types such as dendritic cells, natural killer cells and macrophages in any of the groups studied. However, the involvement of NK and NKT (NK cells carrying T cell receptor) was suggested by the increased expression of GZMM and CD1d genes respectively in the NPR patients. CD1d alone was detected in self-recovering PR patients during the acute phase and was part of pregnancy-associated immunomodulation. Previously our lab recorded higher proportion of activated CD16+ Cd56+/CD3+ cells in the NPR patients [62]. In view of this, it is interesting to note that in a transgenic HBV transfer mouse model, induction of acute hepatitis was mediated by CD1D-restricted NKT cells [63]. Myocarditis in BALB/c was shown to be mediated by CD1D-restricted immune response. For RSV, LCMV, CMV and HSV1, a beneficial effect was seen. The role of NK cells in determining fulminant outcome of HEV infection needs to be evaluated. Similarly, our data pointing possible involvement of T regulatory cells in subclinical infection during 2^nd^ trimester should be confirmed and extended to severe disease.

The absence of induction of IFNγ gene during acute phase irrespective of pregnancy and 2.99fold increase during convalescence among NPR patients is noteworthy and in accordance with our earlier observations of (i) a significant reduction in the circulating IFNγ levels in the NPR patients that were further reduced in the PR patients [40]. (ii) no significant release of IFNγin the culture supernatants of rORF2-stimulated PBMCs(acute phase) coupled with (iii) a significant increase in IFNγ release when the samples were collected during convalescence [24]. In the FHF patients, IFNγ levels increased significantly, a further rise marked recovery. Patients with unaltered IFNγ levels succumbed to the infection [24]. These results suggest protective role of this Th1 cytokine. Majumdar et al [27] confirmed these findings. The concordance in the expression at gene and protein levels emphasizes definite role of IFNγ in recovery even from fulminant disease. Further studies are needed to explore the role / utility of this gene/molecule in modulation /treatment of severe disease.

## Conclusions

Our data documented heightened innate immune response in terms of highly upregulated expression of transcripts associated with non-specific primary response and robust inflammatory response in the pregnant women with self-recovering hepatitis E. The study revealed that immune responses during the 2^nd^ and 3^rd^ trimesters were distinctly different and recorded definite differences between clinical and subclinical infections during these trimesters. Disease presentation was associated with differential expression of S100 series, inversely proportion to overexpression of immunoglobulin (Ig) genes. Possible involvement of TLR4 and NOD1 in the pregnant patients and alpha defensins in all patient categories suggested a role in protection. Based on the confirmation of the gene expression profiles obtained in this study by several functional studies reported earlier in acute as well as fulminant hepatitis patients with or without pregnancy, it may be summarized that clinical presentation in hepatitis E represent distinct transcriptional profiles with respect to immune response. The extensive, informative data provided for the first time should form basis for future studies that will help in understanding pathogenesis of fulminant hepatitis E during pregnancy.

## Supporting information

S1 Fig Hierarchical clustering of differentially expressed genes in PBMCs of healthy pregnant controls and HEV infected patients. Hierarchical clustering of differentially expressed genes with altered expression in healthy pregnant trimester 2 (C), 3 (D) control groups, sub-clinical (J and K), acute HEV infected pregnant trimester 2 (G) and 3 (H) and non-pregnant acute (E) patients as compared to healthy non-pregnant control group (A).A heat map illustrates two-dimensional hierarchical clustering of selected 384 immune-related genes identified as differentially regulated in early acute non-pregnant patients, pregnant women during trimester 2 and 3 with subclinical and clinical infection and healthy pregnant women in trimester 2 and 3 versus healthy non-pregnant controls. On the heat map the rows represent m RNA quantitation data (log2 transformed fold change) and the columns represent the RNA samples isolated from PBMCs. Increased and decreased expression of specific genes is illustrated by red and green, respectively, while black indicates no change.
(XLSX)

S2 Fig Number of significant down-regulated genes among acute non-pregnant and pregnant HEV infected patients. Venn diagram showing the number of down-regulated genes uniquely expressed by NPR-acute (orange), PR-2-acute (green) and PR-3-acute (violet) patients and shadows of corresponding colors denote genes commonly expressed in the respective patient groups. Differential expression analysis was done by comparing the PR-2-acute and PR-3-acute patients with respective healthy trimester controls (PR-2-control and PR-3-control) and NPR-acute as compared to healthy non-pregnant controls (NPR-control).
(TIF)

S1 Table Mapping summary of the sample reads to reference hg19 genome. (DOCX)

S2 Table Mapping Summary: Exonic rate, coverage and number of transcripts. (DOCX)

S3 Table Significantly altered genes in acute (NPR-acute) and convalescent (NPR-conv) phase patients with HEV infection with pair-wise comparison with non-pregnant healthy controls (NPR-control). (DOCX)

S4 Table Significantly altered genes in acute (NPR-acute) and convalescent (NPR-conv) phase patients with HEV infection with pair-wise comparison with non-pregnant healthy controls (NPR-control). (DOCX)

S5 Table Significantly altered genes in acute NPR-acute, PR-2-acute and PR-3-acute patients with HEV infection with pair-wise comparison with non-pregnant healthy controls (NPR-control). (DOCX)

S6 Table Significantly altered genes in acute (PR-2-acute) and subclinical (PR-2-SC) HEV infections in pregnant women in the 2^nd^ trimester with pair-wise comparisons done with non-pregnant healthy controls (NPR-control). (DOCX)

S7 Table Significantly altered genes in acute (PR-2-acute) and subclinical (PR-2-SC) HEV infections in pregnant women in the 2^nd^ trimester with pair-wise comparisons done with non-pregnant healthy controls (NPR-control). (DOCX)

S8 Table Significantly altered genes in acute (PR-3-acute) and subclinical (PR-3-SC) HEV infections in pregnant women in the 3rd trimester with pair-wise comparisons done with non-pregnant healthy controls (NPR-control). (DOCX)

S9 Table Significantly altered genes in acute (PR-3-acute) and subclinical (PR-3-SC) HEV infections in pregnant women in the 3rd trimester with pair-wise comparisons done with non-pregnant healthy controls (NPR-control). (DOCX)

S10 Table Significantly altered genes in acute NPR-acute, PR-2-acute and PR-3-acute patients with HEV infection with pair-wise comparison done with respective healthy pregnant controls (PR-2-control and PR-3-control). (DOCX)

S11 Table Significantly altered genes in acute (PR-2-acute) and subclinical (PR-2-SC) HEV infections in the pregnant 2^nd^ trimester women with pair-wise comparison done with respective healthy pregnant controls. (DOCX)

S12 Table Significantly altered genes in acute (PR-2-acute) and subclinical (PR-2-SC) HEV infections in the pregnant 2^nd^ trimester women with pair-wise comparison done with respective healthy pregnant controls. (DOCX)

S13 Table Significantly altered genes in acute (PR-3-acute) and subclinical (PR-3-SC) HEV infections in the pregnant 3^rd^ trimester women with pair-wise comparison done with respective healthy pregnant controls. (DOCX)

S14 Table List of primer sequences used for SYBR green-based Real Time PCR assays. (DOCX)
