# Supplementary figures and images for: The expression patterns of immune response genes in the Peripheral Blood Mononuclear cells of pregnant women presenting with subclinical or clinical HEV infection are different and trimester-dependent: A whole transcriptome analysis
Source: PLoS One. 2020 Feb 3;15(2):e0228068. doi: 10.1371/journal.pone.0228068 (PMC6996850; doi:10.1371/journal.pone.0228068)

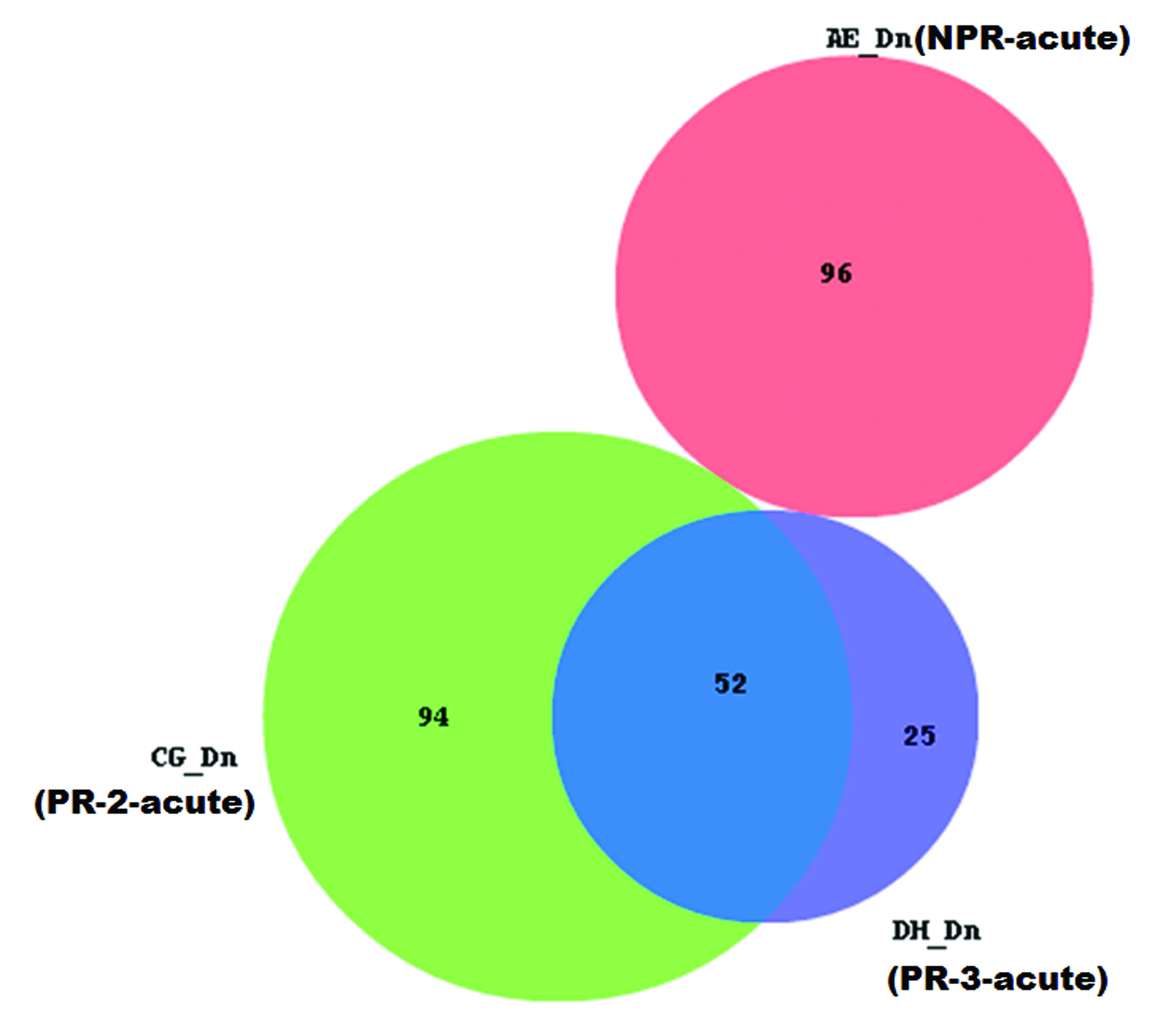

Supplement: S2 Fig — Venn diagram showing the number of down-regulated genes uniquely expressed by NPR-acute (orange), PR-2-acute (green) and PR-3-acute (violet) patients and shadows of corresponding colors denote genes commonly expressed in the respective patient groups. Differential expression analysis was done by comparing the PR-2-acute and PR-3-acute patients with respective healthy trimester controls (PR-2-control and PR-3-control) and NPR-acute as compared to healthy non-pregnant controls (NPR-control). (TIF) [file pone.0228068.s002.tif]
