# Supplementary material for: The expression patterns of immune response genes in the Peripheral Blood Mononuclear cells of pregnant women presenting with subclinical or clinical HEV infection are different and trimester-dependent: A whole transcriptome analysis
Source: PLoS One. 2020 Feb 3;15(2):e0228068. doi: 10.1371/journal.pone.0228068 (PMC6996850; doi:10.1371/journal.pone.0228068)
Supplement: S1 Table — (DOCX) [file pone.0228068.s003.docx]

**Table S1 -. Summary of Mapping to the HG19 genome**

| **Study group name** | **Sample Label** | **# Total Reads** | **After rRNA filter** | **Mapped (GRCh37.68)** | **% Mapped** | **Uniquely Mapped** | **Unmapped** |
| --- | --- | --- | --- | --- | --- | --- | --- |
| **NPR-control** | **Control NPR_BC 9** | **119,322,023** | **117,317,620** | **50,209,314** | **42.8** | **41,625,791** | **75,691,829** |
|  | **Control NPR_BC 10** | **121,325,291** | **119,404,960** | **51,604,907** | **43.2** | **43,475,202** | **75,929,758** |
|  | **Control NPR_BC 11** | **96,376,003** | **94,582,723** | **38,640,221** | **40.9** | **31,981,430** | **62,601,293** |
|  | **Control NPR_BC 12** | **121,602,255** | **119,694,612** | **52,221,601** | **43.6** | **43,429,170** | **76,265,442** |
| **PR-2-control** | **lib2_ANCcontrol2_pool1** | **133,095,595** | **105,276,394** | **33,878,331** | **32.2** | **27,661,424** | **77,614,970** |
|  | **lib2_ANCcontrol2_pool2** | **151,526,968** | **120,855,752** | **42,582,929** | **35.2** | **33,884,706** | **86,971,046** |
|  | **lib2_ANCcontrol2_pool3** | **171,788,821** | **135,538,122** | **46,838,187** | **34.6** | **38,570,254** | **96,967,868** |
|  | **lib2_ANCcontrol2_pool4** | **86,648,972** | **62,918,562** | **17,771,685** | **28.2** | **14,578,387** | **48,340,175** |
| **PR-2-acute and PR-3-acute** | **CLINICAL_ANC_ICK_BC_10** | **122,534,547** | **120,751,570** | **51,061,471** | **42.3** | **41,075,088** | **79,676,482** |
|  | **CLINICAL_ANC_ICK_BC_11** | **45,969,436** | **45,264,879** | **19,211,231** | **42.4** | **14,883,429** | **30,381,450** |
|  | **CLINICAL_ANC_ICK_BC_12** | **171,157,397** | **168,937,995** | **72,992,140** | **43.2** | **58,233,366** | **110,704,629** |
|  | **CLINICAL_ANC_ICK_BC_9** | **206,903,440** | **204,157,053** | **86,244,052** | **42.2** | **69,925,024** | **134,232,029** |
| **PR-2-acute and PR-3-acute** | **CLINICAL_ANC_2_BC_5** | **114,453,692** | **112,618,000** | **45,449,810** | **40.4** | **39,430,282** | **73,187,718** |
|  | **CLINICAL_ANC_2_BC_6** | **113,716,817** | **111,192,209** | **54,863,569** | **49.3** | **43,826,648** | **67,365,561** |
|  | **CLINICAL_ANC_3_BC_7** | **140,986,617** | **139,083,029** | **56,425,245** | **40.6** | **46,548,254** | **92,534,775** |
|  | **CLINICAL_ANC_3_BC_8** | **117,842,792** | **115,559,715** | **44,311,701** | **38.3** | **35,876,598** | **79,683,117** |
| **PR-2-SC and PR-3-SC** | **SUB_ANC_2_BC_5** | **155,469,078** | **153,275,806** | **35,324,819** | **23.0** | **31,246,410** | **122,029,396** |
|  | **SUB_ANC_3_BC_7** | **144,225,359** | **141,434,533** | **38,178,686** | **27.0** | **31,313,529** | **110,121,004** |
|  | **SUB_ANC_3_BC_8** | **130,646,354** | **128,511,026** | **36,453,854** | **28.4** | **30,453,164** | **98,057,862** |
| **NPR-acute and NPR-conv** | **lib1_HEV17_12_2012_Early_acute_pool3** | **86,795,525** | **75,038,583** | **21,996,716** | **29.3** | **17,227,394** | **57,811,189** |
|  | **lib1_HEV17_12_2012_Late_pool_1** | **183,024,280** | **161,740,779** | **47,922,971** | **29.6** | **38,509,240** | **123,231,539** |
|  | **lib1_HEV17_12_2012_Early_acute_pool4** | **163,773,610** | **144,410,776** | **46,697,199** | **32.3** | **37,093,782** | **107,316,994** |
|  | **lib1_HEV17_12_2012_Late_pool_2** | **100,413,432** | **89,015,963** | **32,532,248** | **36.5** | **26,692,372** | **62,323,591** |
| **NPR-acute** | **ACUTE_NPR_BC_6_ICK_4** | **148,135,058** | **127,420,993** | **28,307,332** | **22.2** | **22,935,624** | **104,485,369** |
|  | **ACUTE_NPR_BC_7_E1** | **145,062,795** | **128,637,086** | **27,753,232** | **21.6** | **21,412,644** | **107,224,442** |
|  | **ACUTE_NPR_BC_8_E2** | **178,877,054** | **157,152,915** | **33,855,317** | **21.5** | **26,535,196** | **130,617,719** |
| **NPR-aacute** | **ACUTE_NPR_EARLY_BC_1** | **155,473,295** | **137,152,487** | **28,273,760** | **20.6** | **22,460,150** | **114,692,337** |
|  | **ACUTE_NPR_EARLY_BC_2** | **143,725,901** | **128,699,411** | **28,965,475** | **22.5** | **23,384,257** | **105,315,154** |
| **PR-2-SC and PR-3-SC** | **SUBCLINICAL_ANC_2_BC_5** | **162,671,141** | **160,369,472** | **45,124,368** | **28.1** | **39,658,197** | **120,711,275** |
|  | **SUBCLINICAL_ANC_3_BC_7** | **154,266,276** | **151,302,180** | **50,864,196** | **33.6** | **41,552,649** | **109,749,531** |
|  | **SUBCLINICAL_ANC_3_BC_8** | **139,404,941** | **137,104,009** | **47,716,811** | **34.8** | **39,668,973** | **97,435,036** |
|  | **CONTROL_ANC3_POOL1** | **126,673,167** | **95,253,682** | **25,935,343** | **27.2** | **20,710,656** | **74,543,026** |
| **PR-3-control** | **CONTROL_ANC_3_POOL_2** | **109,129,117** | **84,569,315** | **33,092,165** | **39.1** | **26,953,733** | **57,615,582** |
|  | **CONTROL_ANC_3_POOL_3** | **104,009,373** | **81,933,652** | **31,936,062** | **39.0** | **25,935,129** | **55,998,523** |
|  | **CONTROL_ANC_3_POOL_4** | **145,865,069** | **118,838,529** | **34,791,330** | **29.3** | **27,425,469** | **91,413,060** |
|  |  |  |  |  |  |  |  |
