# Supplementary material for: The expression patterns of immune response genes in the Peripheral Blood Mononuclear cells of pregnant women presenting with subclinical or clinical HEV infection are different and trimester-dependent: A whole transcriptome analysis
Source: PLoS One. 2020 Feb 3;15(2):e0228068. doi: 10.1371/journal.pone.0228068 (PMC6996850; doi:10.1371/journal.pone.0228068)
Supplement: S2 Table — (DOCX) [file pone.0228068.s004.docx]

**Table S2 Summary of mapping including exonic rate**

| **Study group name**  **(Sequencing run name)** | **Sample Label** | **Intragenic Rate (%)** | **Exonic Rate (%)** | **Intronic Rate (%)** | **Expression Profiling Efficiency** | **Intergenic Rate (%)** | **# of Transcripts Detected** | **# of Genes Detected** |
| --- | --- | --- | --- | --- | --- | --- | --- | --- |
| NPR-control  (Control_NPR_BC) | Control NPR_BC 9 | 97.6 | 74.3 | 23.3 | 74.3 | 2.4 | 114,886 | 21,787 |
|  | Control NPR_BC 10 | 97.6 | 76.0 | 21.6 | 76.0 | 2.4 | 115,807 | 21,951 |
|  | Control NPR_BC 11 | 97.3 | 72.7 | 24.7 | 72.7 | 2.7 | 112,578 | 21,148 |
|  | Control NPR_BC 12 | 97.8 | 77.4 | 20.4 | 77.4 | 2.2 | 115,471 | 21,744 |
| PR-2-control  (ANCcontrol2) | lib2_ANCcontrol2_pool1 | 96.0 | 61.7 | 34.4 | 61.7 | 3.9 | 109,570 | 20,841 |
|  | lib2_ANCcontrol2_pool2 | 96.4 | 76.4 | 20.0 | 76.4 | 3.5 | 112,769 | 21,293 |
|  | lib2_ANCcontrol2_pool3 | 96.7 | 76.0 | 20.7 | 76.0 | 3.3 | 114,375 | 21,829 |
|  | lib2_ANCcontrol2_pool4 | 96.0 | 63.9 | 32.1 | 63.9 | 4.0 | 99,229 | 18,169 |
| PR-2-acute and PR-3-acute  (BCCLINICAL) | CLINICAL_ANC_ICK_BC_10 | 98.3 | 86.6 | 11.7 | 86.6 | 1.7 | 110,306 | 20,081 |
|  | CLINICAL_ANC_ICK_BC_11 | 98.5 | 87.4 | 11.1 | 87.4 | 1.5 | 89,712 | 15,738 |
|  | CLINICAL_ANC_ICK_BC_12 | 98.7 | 88.6 | 10.1 | 88.6 | 1.3 | 107,612 | 19,736 |
|  | CLINICAL_ANC_ICK_BC_9 | 98.2 | 85.4 | 12.8 | 85.4 | 1.8 | 116,225 | 21,975 |
| PR-2-acute and PR-3-acute  (BCCLINICAL_ANC_2AND3) | CLINICAL_ANC_2_BC_5 | 97.9 | 84.1 | 13.8 | 84.1 | 2.1 | 110,443 | 20,275 |
|  | CLINICAL_ANC_2_BC_6 | 97.8 | 79.8 | 18.0 | 79.8 | 2.2 | 112,693 | 21,067 |
|  | CLINICAL_ANC_3_BC_7 | 98.1 | 85.9 | 12.2 | 85.9 | 1.9 | 115,133 | 21,300 |
|  | CLINICAL_ANC_3_BC_8 | 97.4 | 78.7 | 18.6 | 78.7 | 2.6 | 113,204 | 21,079 |
| PR-1-control  (BC_CONTROL_ANC1_24_7_13) | CONTROL_ANC1_BC5 | 96.8 | 74.4 | 22.4 | 74.4 | 3.2 | 113,454 | 21,381 |
|  | CONTROL_ANC_1_BC6 | 97.4 | 78.9 | 18.5 | 78.9 | 2.5 | 113,749 | 21,364 |
|  | CONTROL_ANC_1_BC_7 | 96.4 | 71.8 | 24.6 | 71.8 | 3.6 | 97,002 | 17,546 |
|  | CONTROL_ANC1_BC_8 | 96.4 | 71.6 | 24.7 | 71.6 | 3.6 | 101,676 | 18,606 |
| PR-1-SC, PR-2-SC and PR-3-SC  (BCSUBCLINICAL) | SUB_ANC_1_BC_6 | 97.1 | 77.0 | 20.1 | 77.0 | 2.9 | 112,771 | 21,248 |
|  | SUB_ANC_2_BC_5 | 96.3 | 69.4 | 26.9 | 69.4 | 3.7 | 114,405 | 21,549 |
|  | SUB_ANC_3_BC_7 | 96.6 | 73.6 | 23.0 | 73.6 | 3.4 | 113,782 | 21,224 |
|  | SUB_ANC_3_BC_8 | 96.9 | 76.1 | 20.9 | 76.1 | 3.1 | 112,924 | 20,954 |
| NPR-acute and NPR-conv  (HEVacuteNPR_pool1) | lib1_HEV17_12_2012_Early_acute_pool3 | 96.9 | 81.2 | 15.7 | 81.2 | 3.1 | 100,572 | 18,122 |
|  | lib1_HEV17_12_2012_Late_pool_1 | 97.3 | 82.8 | 14.5 | 82.8 | 2.7 | 113,988 | 21,528 |
|  | lib1_HEV17_12_2012_Early_acute_pool4 | 97.2 | 81.1 | 16.1 | 81.1 | 2.8 | 113,699 | 21,343 |
|  | lib1_HEV17_12_2012_Late_pool_2 | 97.4 | 81.7 | 15.7 | 81.7 | 2.6 | 106,322 | 19,507 |
| NPR-acute  (BC_NPR) | ACUTE_NPR_BC_5_ICK3 | 95.7 | 81.2 | 14.5 | 81.2 | 4.3 | 102,131 | 18,363 |
|  | ACUTE_NPR_BC_6_ICK_4 | 96.9 | 83.2 | 13.8 | 83.2 | 3.1 | 104,483 | 18,997 |
|  | ACUTE_NPR_BC_7_E1 | 96.4 | 79.4 | 17.0 | 79.4 | 3.6 | 107,058 | 19,633 |
|  | ACUTE_NPR_BC_8_E2 | 97.0 | 82.3 | 14.7 | 82.3 | 3.0 | 105,165 | 19,294 |
| NPR-control and NPR-acute  (MALE_CONTROL_NPR) | ACUTE_NPR_EARLY_BC_1 | 96.5 | 80.1 | 16.4 | 80.1 | 3.5 | 104,037 | 18,999 |
|  | ACUTE_NPR_EARLY_BC_2 | 96.8 | 80.2 | 16.6 | 80.2 | 3.2 | 107,601 | 19,712 |
|  | CONTROL_MALE_NPR_BC_3 | 95.4 | 70.0 | 25.3 | 70.0 | 4.6 | 101,361 | 18,204 |
|  | CONTROL_MALE_NPR_BC_4 | 95.3 | 75.6 | 19.7 | 75.6 | 4.7 | 99,477 | 17,745 |
| PR-1-SC, PR-2-SC and PR-3-SC  (SUBCLINICAL_ANC_9_8_13) | SUBCLINICAL_ANC_1_BC_6 | 97.3 | 78.1 | 19.2 | 78.1 | 2.7 | 114,991 | 21,873 |
|  | SUBCLINICAL_ANC_2_BC_5 | 96.7 | 72.3 | 24.4 | 72.3 | 3.3 | 116,690 | 22,281 |
|  | SUBCLINICAL_ANC_3_BC_7 | 97.0 | 76.1 | 20.9 | 76.1 | 3.0 | 116,533 | 21,986 |
|  | SUBCLINICAL_ANC_3_BC_8 | 97.3 | 78.4 | 18.9 | 78.4 | 2.7 | 115,429 | 21,649 |
| PR-3-control  (control_anc_3) | CONTROL_ANC3_POOL1 | 96.6 | 71.8 | 24.8 | 71.8 | 3.4 | 102,815 | 18,868 |
|  | CONTROL_ANC_3_POOL_2 | 96.4 | 73.3 | 23.1 | 73.3 | 3.5 | 109,516 | 20,486 |
|  | CONTROL_ANC_3_POOL_3 | 96.4 | 70.7 | 25.7 | 70.7 | 3.5 | 109,317 | 20,538 |
|  | CONTROL_ANC_3_POOL_4 | 96.8 | 71.8 | 24.9 | 71.8 | 3.2 | 105,561 | 19,875 |
