# Supplementary material for: The expression patterns of immune response genes in the Peripheral Blood Mononuclear cells of pregnant women presenting with subclinical or clinical HEV infection are different and trimester-dependent: A whole transcriptome analysis
Source: PLoS One. 2020 Feb 3;15(2):e0228068. doi: 10.1371/journal.pone.0228068 (PMC6996850; doi:10.1371/journal.pone.0228068)
Supplement: S3 Table — (DOCX) [file pone.0228068.s005.docx]

**Significantly altered genes in acute (NPR-acute) and convalescent (NPR-conv) phase of disease in NPR patients with pair-wise comparison with non-pregnant healthy control.**

**Table S5- List of significantly up-regulated genes:**

| **Gene short name** | **NPR-acute** | | **NPR-conv** | |
| --- | --- | --- | --- | --- |
|  | **Log2 fold change** | **Q value** | **Log2 fold change** | **Q value** |
| AQP3 | 2.85 | 0.0346 | 3.41 | 0.0000 |
| BCL2A1 | 2.66 | 0.0001 | 2.59 | 0.0000 |
| C1QA | 3.92 | 0.0004 | 3.14 | 0.0002 |
| C1QB | 2.39 | 0.0069 | 1.46 | 0.0484 |
| CAMP | 3.77 | 0.0136 | 2.22 | 0.0082 |
| CCL2 | 3.46 | 0.0190 | 4.48 | 0.0000 |
| CCL3 | 2.70 | 0.0140 | 3.23 | 0.0000 |
| CCL4 | 2.07 | 0.0885 | 2.25 | 0.0000 |
| CCR1 | 1.87 | 0.0433 | 1.85 | 0.0000 |
| CD1D | 1.87 | 0.0082 | 1.93 | 0.0000 |
| CD48 | 2.21 | 0.0059 | 2.49 | 0.0000 |
| CEBPB | 1.86 | 0.0067 | 1.65 | 0.0000 |
| CST7 | 1.79 | 0.0183 | 2.64 | 0.0000 |
| DEFA1 | 3.50 | 0.0244 | 1.92 | 0.0067 |
| DNASE2 | 3.13 | 0.0004 | 3.11 | 0.0000 |
| FCER1G | 1.94 | 0.0332 | 1.82 | 0.0000 |
| FFAR2 | 3.78 | 0.0000 | 2.34 | 0.0000 |
| FIS1 | 3.60 | 0.0093 | 3.02 | 0.0000 |
| GADD45B | 2.16 | 0.0019 | 2.30 | 0.0000 |
| GZMA | 1.60 | 0.0458 | 1.50 | 0.0000 |
| GZMM | 2.14 | 0.0218 | 2.62 | 0.0000 |
| HBXIP | 1.94 | 0.0973 | 2.00 | 0.0001 |
| HSPA1A | 2.31 | 0.0272 | 3.77 | 0.0000 |
| HSPA1B | 2.96 | 0.0063 | 4.40 | 0.0000 |
| ICAM3 | 1.43 | 0.0819 | 1.25 | 0.0008 |
| ID1 | 4.79 | 0.0900 | 4.74 | 0.0155 |
| IER3 | 3.06 | 0.0000 | 3.12 | 0.0000 |
| IER5L | 3.16 | 0.0397 | 2.70 | 0.0317 |
| IGHG1 | 2.04 | 0.0515 | 3.01 | 0.0000 |
| IGHG3 | 1.72 | 0.0311 | 2.56 | 0.0000 |
| IGHG4 | 1.72 | 0.0399 | 2.71 | 0.0000 |
| IGHGP | 1.92 | 0.0126 | 2.57 | 0.0000 |
| IGHM | 1.61 | 0.0695 | 1.99 | 0.0000 |
| IGHV6-1 | 2.42 | 0.0159 | 2.19 | 0.0003 |
| IGKV1-27 | 2.62 | 0.0064 | 3.12 | 0.0000 |
| IGKV1-33 | 1.89 | 0.0788 | 2.59 | 0.0000 |
| IGKV1-5 | 1.74 | 0.0353 | 2.22 | 0.0000 |
| IGKV1-9 | 2.09 | 0.0440 | 2.78 | 0.0000 |
| IGKV1D-33 | 1.88 | 0.0805 | 2.59 | 0.0000 |
| IGKV3-11 | 2.84 | 0.0001 | 3.11 | 0.0000 |
| IGKV3-20 | 2.19 | 0.0019 | 3.23 | 0.0000 |
| IGKV3D-11 | 2.72 | 0.0003 | 3.15 | 0.0000 |
| IGKV3D-20 | 2.00 | 0.0117 | 3.08 | 0.0000 |
| IGKV4-1 | 1.75 | 0.0206 | 2.42 | 0.0000 |
| IGLC1 | 2.57 | 0.0003 | 3.55 | 0.0000 |
| IGLC2 | 2.07 | 0.0029 | 2.78 | 0.0000 |
| IGLC3 | 2.11 | 0.0020 | 2.83 | 0.0000 |
| IGLV10-54 | 3.51 | 0.0033 | 3.18 | 0.0001 |
| IGLV1-40 | 2.54 | 0.0347 | 3.24 | 0.0000 |
| IGLV1-44 | 2.12 | 0.0263 | 2.45 | 0.0000 |
| IGLV1-47 | 2.34 | 0.0112 | 2.70 | 0.0000 |
| IGLV2-11 | 2.04 | 0.0381 | 3.80 | 0.0000 |
| IGLV2-14 | 2.73 | 0.0001 | 3.06 | 0.0000 |
| IGLV2-23 | 2.69 | 0.0006 | 3.32 | 0.0000 |
| IGLV2-8 | 2.51 | 0.0018 | 3.25 | 0.0000 |
| IGLV3-10 | 2.61 | 0.0047 | 3.72 | 0.0000 |
| IGLV4-69 | 2.96 | 0.0208 | 3.83 | 0.0000 |
| JUN | 4.21 | 0.0000 | 5.06 | 0.0000 |
| JUND | 2.05 | 0.0036 | 2.23 | 0.0000 |
| MTRNR2L9 | 9.37 | 0.0005 | 9.16 | 0.0005 |
| NDUFA13 | 4.52 | 0.0003 | 4.35 | 0.0000 |
| NDUFA7 | 3.90 | 0.0000 | 3.09 | 0.0000 |
| NDUFB11 | 2.62 | 0.0080 | 2.96 | 0.0000 |
| NDUFB7 | 2.33 | 0.0072 | 2.12 | 0.0001 |
| NDUFC2 | 2.15 | 0.0823 | 1.94 | 0.0100 |
| PF4 | 3.65 | 0.0000 | 2.77 | 0.0000 |
| PRDX5 | 3.17 | 0.0265 | 2.88 | 0.0000 |
| PTGES | 4.72 | 0.0000 | 3.93 | 0.0000 |
| RPS19 | 5.22 | 0.0000 | 5.01 | 0.0000 |
| S100A8 | 2.28 | 0.0231 | 1.88 | 0.0000 |
| S100A9 | 2.22 | 0.0782 | 1.37 | 0.0003 |
| S100A11 | 2.47 | 0.0004 | 2.16 | 0.0000 |
| TGM2 | 4.24 | 0.0010 | 2.89 | 0.0016 |
| TMSB4Y | 7.71 | 0.0627 | 8.28 | 0.0243 |
| USMG5 | 3.45 | 0.0007 | 3.35 | 0.0000 |
| CEACAM6 | 3.50 | 0.024637 | - | - |
| CEBPE | 3.06 | 0.098163 | - | - |
| G0S2 | 2.34 | 0.001842 | - | - |
| IFI27 | 5.76 | 0.019859 | - | - |
| P2RY1 | 2.01 | 0.055753 | - | - |
| PI3 | 6.35 | 2.41E-05 | - | - |
| SLPI | 3.81 | 0.020686 | - | - |
| BAX | - | - | 2.73 | 0.000693 |
| CCL3L3 | - | - | 2.78 | 6.54E-07 |
| CCL7 | - | - | 1.8E+308 | 0.090674 |
| CCR4 | - | - | 2.08 | 4.79E-10 |
| CCR6 | - | - | 2.40 | 7.96E-06 |
| CCR7 | - | - | 1.57 | 1.58E-06 |
| CCRL2 | - | - | 1.47 | 0.054144 |
| CD109 | - | - | 1.77 | 0.022155 |
| CD2 | - | - | 1.28 | 0.042959 |
| CD247 | - | - | 1.30 | 0.01337 |
| CD28 | - | - | 2.40 | 3.22E-05 |
| CD55 | - | - | 1.35 | 0.001188 |
| CD69 | - | - | 2.26 | 2.34E-09 |
| CD79A | - | - | 1.10 | 0.018299 |
| CD79B | - | - | 2.26 | 0.002209 |
| CD86 | - | - | 1.37 | 0.052834 |
| CD8A | - | - | 1.38 | 0.063765 |
| CD8B | - | - | 1.29 | 0.003559 |
| CD96 | - | - | 1.81 | 0.031212 |
| CFD | - | - | 1.48 | 0.030981 |
| COX5B | - | - | 1.83 | 0.00044 |
| CXCR4 | - | - | 1.91 | 2.37E-05 |
| CXCR6 | - | - | 1.65 | 0.041748 |
| DAPK3 | - | - | 2.18 | 0.018531 |
| DDIT3 | - | - | 3.36 | 0.013177 |
| DDIT4 | - | - | 1.92 | 0.002293 |
| DEFA3 | - | - | 1.69 | 0.093063 |
| DUSP10 | - | - | 2.33 | 8.87E-05 |
| FCGRT | - | - | 1.13 | 0.017849 |
| FCN2 | - | - | 3.80 | 0.058727 |
| HERPUD1 | - | - | 1.85 | 0.005176 |
| HLA-A | - | - | 1.61 | 0.000234 |
| HLA-DQA2 | - | - | 1.18 | 0.005559 |
| HLA-DQB1 | - | - | 1.42 | 0.058914 |
| HLA-DQB2 | - | - | 1.76 | 0.000734 |
| HLA-DRA | - | - | 1.82 | 0.07985 |
| HLA-DRB1 | - | - | 1.43 | 6.55E-05 |
| HLA-F | - | - | 1.17 | 0.074319 |
| HLA-G | - | - | 1.48 | 0.001143 |
| HMOX1 | - | - | 1.49 | 0.013563 |
| ICOS | - | - | 1.90 | 6.7E-07 |
| IFI30 | - | - | 1.34 | 0.002067 |
| IFI6 | - | - | 1.54 | 2.75E-05 |
| IFITM1 | - | - | 1.66 | 4.44E-06 |
| IFITM3 | - | - | 1.38 | 0.020566 |
| IFNG | - | - | 2.99 | 3.35E-05 |
| IFNGR1 | - | - | 1.23 | 0.040184 |
| IGHG2 | - | - | 2.26 | 6.73E-11 |
| IGHV1-2 | - | - | 1.55 | 0.028528 |
| IGHV1-69 | - | - | 1.58 | 0.026112 |
| IGHV2-5 | - | - | 2.85 | 4.66E-06 |
| IGHV3-15 | - | - | 1.38 | 0.083159 |
| IGHV3-30 | - | - | 1.72 | 0.000954 |
| IGHV3-33 | - | - | 1.55 | 0.005987 |
| IGHV3-43 | - | - | 1.86 | 0.01684 |
| IGHV3-72 | - | - | 2.16 | 0.000593 |
| IGHV4-31 | - | - | 1.87 | 0.015515 |
| IGHV4-34 | - | - | 1.45 | 0.043397 |
| IGHV5-51 | - | - | 2.18 | 0.000199 |
| IGKC | - | - | 1.83 | 0.000742 |
| IGKV1-17 | - | - | 2.94 | 4.58E-07 |
| IGKV1-32 | - | - | 3.13 | 0.000291 |
| IGKV1-37 | - | - | 2.89 | 0.001176 |
| IGKV1-6 | - | - | 1.29 | 0.067982 |
| IGKV1-8 | - | - | 2.40 | 0.00105 |
| IGKV1D-12 | - | - | 1.70 | 0.015498 |
| IGKV1D-17 | - | - | 2.38 | 0.000436 |
| IGKV1D-37 | - | - | 2.73 | 0.013876 |
| IGKV1D-42 | - | - | 2.78 | 0.024692 |
| IGKV1D-8 | - | - | 2.97 | 4.33E-05 |
| IGKV2-24 | - | - | 2.05 | 0.000637 |
| IGKV2-30 | - | - | 1.66 | 0.00347 |
| IGKV2D-29 | - | - | 1.76 | 0.012839 |
| IGKV2D-30 | - | - | 1.65 | 0.023145 |
| IGKV3-15 | - | - | 2.37 | 2.6E-08 |
| IGKV3-7 | - | - | 1.77 | 0.030933 |
| IGKV3D-15 | - | - | 2.38 | 6.08E-08 |
| IGKV3D-7 | - | - | 2.38 | 0.000718 |
| IGKV5-2 | - | - | 4.07 | 2.04E-05 |
| IGKV6-21 | - | - | 4.97 | 0 |
| IGLV1-36 | - | - | 2.41 | 0.006499 |
| IGLV1-41 | - | - | 2.47 | 0.005817 |
| IGLV1-51 | - | - | 3.16 | 2.74E-05 |
| IGLV2-18 | - | - | 2.82 | 0.007186 |
| IGLV2-34 | - | - | 4.46 | 0.005464 |
| IGLV2-5 | - | - | 3.35 | 0.015375 |
| IGLV3-16 | - | - | 3.87 | 4.25E-08 |
| IGLV3-19 | - | - | 2.54 | 1.48E-08 |
| IGLV3-21 | - | - | 1.42 | 0.034245 |
| IGLV3-25 | - | - | 2.35 | 8.68E-06 |
| IGLV3-27 | - | - | 4.15 | 1.22E-06 |
| IGLV3-9 | - | - | 2.65 | 0.000239 |
| IGLV6-57 | - | - | 1.51 | 0.034614 |
| IGLV7-43 | - | - | 2.13 | 0.003702 |
| IGLV8-61 | - | - | 1.61 | 0.003422 |
| IGLV9-49 | - | - | 1.94 | 0.027067 |
| IL1B | - | - | 2.06 | 0.000665 |
| IL23A | - | - | 2.17 | 0.000646 |
| IL3RA | - | - | 1.42 | 0.026244 |
| IL8 | - | - | 2.15 | 4.76E-06 |
| ISG15 | - | - | 1.28 | 0.013522 |
| LGALS1 | - | - | 4.28 | 9.33E-07 |
| LGALS3 | - | - | 2.41 | 3.34E-07 |
| LILRA5 | - | - | 1.81 | 1.74E-05 |
| NDUFA1 | - | - | 1.39 | 0.003953 |
| NDUFA4 | - | - | 1.81 | 0.014844 |
| NDUFA6 | - | - | 1.94 | 0.063676 |
| NDUFAB1 | - | - | 1.84 | 0.011816 |
| NDUFB1 | - | - | 1.03 | 0.093285 |
| NDUFB2 | - | - | 2.20 | 0.021146 |
| NDUFS4 | - | - | 2.52 | 2.79E-05 |
| NDUFS5 | - | - | 1.86 | 3.14E-05 |
| NDUFV2 | - | - | 1.69 | 0.032799 |
| NEDD1 | - | - | 2.59 | 0.00227 |
| NFKBIB | - | - | 1.77 | 0.018494 |
| OASL | - | - | 1.81 | 0.000263 |
| P2RY10 | - | - | 1.45 | 0.006663 |
| P2RY2 | - | - | 1.48 | 0.050923 |
| PHLDA1 | - | - | 2.25 | 0.002292 |
| POMP | - | - | 1.72 | 6.54E-05 |
| PRDX1 | - | - | 1.35 | 0.003723 |
| PRKCQ | - | - | 1.37 | 0.001288 |
| PSMA7 | - | - | 1.44 | 0.003641 |
| RHOB | - | - | 1.26 | 0.00122 |
| RIPK2 | - | - | 1.74 | 0.001779 |
| S100A10 | - | - | 1.55 | 0.001926 |
| S100A6 | - | - | 1.94 | 1.46E-07 |
| SERPINB1 | - | - | 1.33 | 0.065491 |
| SERPINB2 | - | - | 1.85 | 0.016446 |
| SOCS1 | - | - | 2.15 | 0.021895 |
| TNFRSF1B | - | - | 1.87 | 0.029963 |
| TNFRSF4 | - | - | 3.60 | 0.001374 |
| TNFRSF8 | - | - | 1.60 | 0.06466 |
| TNFSF8 | - | - | 1.35 | 0.070482 |
| TRAT1 | - | - | 1.33 | 0.049158 |
| TUBB4B | - | - | 1.37 | 0.002719 |
| UBB | - | - | 1.68 | 4.65E-05 |
| UQCRC1 | - | - | 1.50 | 0.004816 |
| UQCRC2 | - | - | 1.86 | 0.01095 |
| USP9Y | - | - | 7.96 | 0.046237 |
| VCAM1 | - | - | 4.82 | 0.048252 |
| UBA52 | - | - | 1.77 | 1.68E-05 |
