# Supplementary material for: The expression patterns of immune response genes in the Peripheral Blood Mononuclear cells of pregnant women presenting with subclinical or clinical HEV infection are different and trimester-dependent: A whole transcriptome analysis
Source: PLoS One. 2020 Feb 3;15(2):e0228068. doi: 10.1371/journal.pone.0228068 (PMC6996850; doi:10.1371/journal.pone.0228068)
Supplement: S4 Table — (DOCX) [file pone.0228068.s006.docx]

**Table S6- List of significant down-regulated genes:**

| **Gene short name** | **NPR-acute** | | **NPR-conv** | |
| --- | --- | --- | --- | --- |
|  | **Log2 fold change** | **Q value** | **Log2 fold change** | **Q value** |
| ACTG1 | -2.55 | 0.000728 | -2.59 | 1.39E-12 |
| ARHGDIA | -2.74 | 0.003036 | -2.16 | 8.05E-08 |
| CD37 | -2.03 | 0.023413 | -1.87 | 9.25E-06 |
| CD97 | -2.11 | 0.026138 | -2.29 | 3.6E-09 |
| DUSP22 | -2.48 | 0.023155 | -2.64 | 2.1E-08 |
| EP300 | -1.72 | 0.014214 | -1.97 | 4.79E-10 |
| HTATSF1 | -4.34 | 0.038914 | -5.07 | 0.00161 |
| NUAK2 | -1.78 | 0.05479 | -2.36 | 6.46E-10 |
| PDLIM1 | -2.01 | 0.083597 | -2.34 | 6.36E-08 |
| PLAGL2 | -2.53 | 0.000522 | -2.55 | 1.31E-12 |
| PRKCSH | -2.38 | 0.012255 | -2.42 | 9.78E-08 |
| ZFP36 | -2.81 | 2.28E-06 | -2.73 | 0 |
| SP2 | -3.46 | 0.034661 | -3.77 | 8.15E-05 |
| TBX21 | -1.72 | 0.040669 | -1.11 | 0.007109 |
| TICAM1 | -3.55 | 0.000154 | -4.66 | 1.23E-12 |
| TP53 | -3.72 | 0.059638 | -3.41 | 0.039744 |
| TRIM65 | -2.13 | 0.070663 | -2.06 | 3.05E-05 |
| VAV1 | -1.98 | 0.04036 | -2.10 | 1.36E-09 |
| ZNF70 | -3.33 | 0.005969 | -3.44 | 1.25E-06 |
| FOXRED2 | -2.91 | 0.072518 | - | - |
| IGHA1 | -1.55 | 0.080831 | - | - |
| NR4A2 | -2.69 | 0.089897 | - | - |
| PRPF8 | -1.49 | 0.095264 | - | - |
| ARPC3 | - | - | -2.96 | 4.75E-05 |
| BPI | - | - | -2.02 | 0.001437 |
| CD22 | - | - | -1.63 | 0.005851 |
| CD274 | - | - | -2.67 | 0.051382 |
| CD300LB | - | - | -1.18 | 0.008985 |
| CD74 | - | - | -1.68 | 0.003055 |
| CREBBP | - | - | -1.13 | 0.014572 |
| CXCL10 | - | - | -2.13 | 0.001158 |
| DSTN | - | - | -1.58 | 0.019776 |
| DUSP23 | - | - | -1.32 | 0.037082 |
| EPB49 | - | - | -5.36 | 3.1E-11 |
| GBF1 | - | - | -1.83 | 0.020566 |
| ITGB2 | - | - | -2.43 | 0.036643 |
| JAK3 | - | - | -1.32 | 0.039927 |
| LGALS9 | - | - | -2.17 | 3.16E-05 |
| LILRA1 | - | - | -1.94 | 0.08777 |
| MAP2K1 | - | - | -1.85 | 0.002199 |
| MEFV | - | - | -2.59 | 8.59E-10 |
| MMP25 | - | - | -2.58 | 0.01786 |
| MPO | - | - | -1.53 | 0.050934 |
| MYD88 | - | - | -2.30 | 0.007776 |
| PTK2B | - | - | -3.18 | 0.007995 |
| RAB3D | - | - | -1.06 | 0.004909 |
| RARA | - | - | -2.46 | 0.004416 |
| RPTOR | - | - | -1.85 | 0.00403 |
| SIGLEC1 | - | - | -1.60 | 0.015149 |
| SMAD4 | - | - | -1.43 | 0.003276 |
| TNFRSF1A | - | - | -1.75 | 0.010559 |
| TNFRSF21 | - | - | -2.13 | 0.001804 |
| UNC13D | - | - | -1.47 | 0.000179 |
