# Supplementary material for: The expression patterns of immune response genes in the Peripheral Blood Mononuclear cells of pregnant women presenting with subclinical or clinical HEV infection are different and trimester-dependent: A whole transcriptome analysis
Source: PLoS One. 2020 Feb 3;15(2):e0228068. doi: 10.1371/journal.pone.0228068 (PMC6996850; doi:10.1371/journal.pone.0228068)
Supplement: S5 Table — (DOCX) [file pone.0228068.s007.docx]

**Significantly altered genes in NPR-acute and PR-2-acute/PR-3-acute patients in acute phase with pair-wise comparison with non-pregnant healthy controls**

**Table S7- List of significantly up-regulated gene:**

| **Gene short name** | **NPR-acute** | | **PR-2-acute** | | **PR-3-acute** | |
| --- | --- | --- | --- | --- | --- | --- |
|  | **Fold change** | **Q value** | **Fold change** | **Q value** | **Fold change** | **Q value** |
| BCL2A1 | 2.66 | 0.0001 | 3.20 | 0.0000 | 3.69 | 0.0000 |
| CAMP | 3.77 | 0.0136 | 4.64 | 0.0000 | 5.67 | 0.0000 |
| CCL3 | 2.70 | 0.0140 | 2.80 | 0.0000 | 3.17 | 0.0000 |
| CCR1 | 1.87 | 0.0433 | 1.90 | 0.0000 | 2.47 | 0.0000 |
| CD1D | 1.87 | 0.0082 | 1.41 | 0.0059 | 1.79 | 0.0013 |
| CD48 | 2.21 | 0.0059 | 1.81 | 0.0000 | 1.54 | 0.0292 |
| CEBPB | 1.86 | 0.0067 | 1.58 | 0.0003 | 2.03 | 0.0004 |
| CRISP3 | 4.53 | 0.0563 | 5.99 | 0.0000 | 7.05 | 0.0853 |
| DEFA1 | 3.50 | 0.0244 | 5.27 | 0.0000 | 6.23 | 0.0000 |
| DUSP4 | 2.15 | 0.0764 | 3.21 | 0.0000 | 2.61 | 0.0019 |
| FFAR2 | 3.78 | 0.0000 | 3.75 | 0.0000 | 4.03 | 0.0000 |
| G0S2 | 2.34 | 0.0018 | 2.99 | 0.0000 | 3.14 | 0.0000 |
| GADD45B | 2.16 | 0.0019 | 1.83 | 0.0001 | 2.28 | 0.0002 |
| HSPA1A | 2.31 | 0.0272 | 3.66 | 0.0000 | 3.81 | 0.0000 |
| HSPA1B | 2.96 | 0.0063 | 4.47 | 0.0000 | 4.63 | 0.0000 |
| IER3 | 3.06 | 0.0000 | 2.22 | 0.0001 | 3.08 | 0.0000 |
| IFI27 | 5.76 | 0.0199 | 4.06 | 0.0834 | 5.30 | 0.0247 |
| JUN | 4.21 | 0.0000 | 3.97 | 0.0000 | 4.19 | 0.0000 |
| JUND | 2.05 | 0.0036 | 1.50 | 0.0007 | 1.56 | 0.0141 |
| KRT23 | 3.92 | 0.0007 | 3.73 | 0.0000 | 2.87 | 0.0020 |
| PI3 | 6.35 | 0.0000 | 8.25 | 0.0000 | 6.94 | 0.0000 |
| PLAUR | 2.58 | 0.0006 | 2.04 | 0.0000 | 2.33 | 0.0005 |
| PTGES | 4.72 | 0.0000 | 4.20 | 0.0000 | 5.51 | 0.0000 |
| RPS19 | 5.22 | 0.0000 | 1.71 | 0.0045 | 2.57 | 0.0005 |
| S100A8 | 2.28 | 0.0231 | 1.35 | 0.0056 | 2.24 | 0.0003 |
| SLPI | 3.81 | 0.0207 | 5.77 | 0.0000 | 5.68 | 0.0000 |
| TGM2 | 4.24 | 0.0010 | 3.78 | 0.0000 | 4.82 | 0.0000 |
| AZU1 | - | - | 2.68 | 0.044306 | 3.97 | 0.000293 |
| BPI | - | - | 3.50 | 5.99E-10 | 4.41 | 1.24E-10 |
| CCL3L1 | - | - | 3.10 | 0.009538 | 3.41 | 0.010754 |
| CCL3L3 | - | - | 2.70 | 2.16E-05 | 2.72 | 0.000519 |
| CCRL2 | - | - | 2.98 | 9.27E-08 | 3.82 | 0.000524 |
| CD109 | - | - | 1.92 | 0.035403 | 2.44 | 0.006203 |
| CD177 | - | - | 5.20 | 2.06E-05 | 6.28 | 0.005671 |
| CD300LB | - | - | 1.10 | 0.087767 | 1.82 | 0.000503 |
| CD83 | - | - | 1.84 | 1.47E-06 | 1.54 | 0.004037 |
| CEACAM3 | - | - | 3.13 | 0.026228 | 3.43 | 0.024488 |
| CEACAM5 | - | - | 4.10 | 0.001535 | 4.92 | 0.000182 |
| CEACAM6 | - | - | 5.98 | 0 | 6.95 | 0 |
| CEACAM8 | - | - | 4.98 | 0 | 6.17 | 0 |
| CEBPB | - | - | 1.58 | 0.00034 | 2.03 | 0.000397 |
| CXCL2 | - | - | 2.33 | 0.021632 | 3.17 | 0.001094 |
| CXCR1 | - | - | 2.26 | 0.000159 | 2.78 | 5.38E-05 |
| CXCR4 | - | - | 1.90 | 0.001033 | 1.67 | 0.075246 |
| DDIT3 | - | - | 3.41 | 0.024712 | 3.44 | 0.062548 |
| DDIT4 | - | - | 2.46 | 8.12E-05 | 3.36 | 0.00523 |
| DEFA1B | - | - | 5.19 | 9.38E-14 | 6.16 | 1.64E-10 |
| DEFA3 | - | - | 4.92 | 4.35E-13 | 6.24 | 2.67E-13 |
| DEFA4 | - | - | 3.99 | 2.04E-11 | 5.06 | 0 |
| DHX40 | - | - | 1.55 | 0.000233 | 1.72 | 0.015245 |
| DUSP10 | - | - | 2.68 | 0.000102 | 2.70 | 0.00432 |
| DUSP4 | - | - | 3.21 | 2.4E-07 | 2.61 | 0.00189 |
| ELANE | - | - | 3.98 | 0.001116 | 4.79 | 0.000111 |
| HSPA13 | - | - | 2.19 | 8.72E-05 | 2.49 | 0.001056 |
| ICAM1 | - | - | 1.97 | 0.000449 | 2.45 | 3.14E-05 |
| IER5 | - | - | 1.33 | 0.022026 | 1.43 | 0.080709 |
| IFNG | - | - | 3.65 | 3.59E-07 | 2.94 | 0.001061 |
| IL1B | - | - | 3.23 | 1.43E-07 | 3.12 | 0.000355 |
| IL8 | - | - | 4.24 | 5.43E-12 | 5.50 | 4.12E-05 |
| IRAK2 | - | - | 1.34 | 0.027933 | 1.52 | 0.032305 |
| LEP | - | - | 8.96 | 0.000435 | 10.55 | 1.08E-05 |
| LGALS3 | - | - | 2.31 | 0.002696 | 2.40 | 0.014241 |
| LILRA5 | - | - | 1.19 | 0.072473 | 2.12 | 0.006223 |
| MMP8 | - | - | 6.03 | 1.57E-07 | 6.94 | 1.71E-08 |
| MMP9 | - | - | 4.43 | 3.73E-07 | 5.50 | 6.46E-11 |
| MPO | - | - | 3.13 | 7.64E-11 | 3.95 | 9.38E-14 |
| NFKBIA | - | - | 1.88 | 0.022551 | 2.07 | 0.04393 |
| PGLYRP1 | - | - | 4.85 | 6.92E-07 | 6.56 | 4.35E-13 |
| PLAU | - | - | 5.52 | 0.063655 | 5.85 | 0.074513 |
| POMP | - | - | 1.60 | 0.006659 | 1.60 | 0.037506 |
| REL | - | - | 1.67 | 3.14E-05 | 1.58 | 0.00384 |
| RIPK2 | - | - | 2.13 | 0.000862 | 2.38 | 0.003632 |
| SMAD7 | - | - | 1.68 | 0.000713 | 1.67 | 0.01261 |
| TLR2 | - | - | 1.26 | 0.012942 | 1.83 | 0.000552 |
| TNFAIP6 | - | - | 4.85 | 9.92E-08 | 5.32 | 5.4E-07 |
| TNFRSF10D | - | - | 1.48 | 0.033428 | 1.55 | 0.039547 |
| TREM1 | - | - | 1.21 | 0.022294 | 1.73 | 0.002126 |
| UQCRB | - | - | 1.26 | 0.013476 | 1.36 | 0.091041 |
| VSIG4 | - | - | 1.87 | 0.02433 | 2.11 | 0.02405 |
| AQP3 | 2.85 | 0.034608 | - | - | - | - |
| CCL2 | 3.46 | 0.018989 | - | - | - | - |
| CMTM2 | 3.35 | 0.081737 | - | - | - | - |
| FCER1G | 1.94 | 0.033222 | - | - | - | - |
| FIS1 | 3.60 | 0.00927 | - | - | - | - |
| GP9 | 2.53 | 0.004525 | - | - | - | - |
| GPX1 | 3.48 | 0.006821 | - | - | - | - |
| GZMA | 1.60 | 0.045831 | - | - | - | - |
| GZMM | 2.14 | 0.021807 | - | - | - | - |
| ICAM3 | 1.43 | 0.081872 | - | - | - | - |
| IER5L | 3.16 | 0.039663 | - | - | - | - |
| IGHG1 | 2.04 | 0.051492 | - | - | - | - |
| IGHG3 | 1.72 | 0.031085 | - | - | - | - |
| IGHG4 | 1.72 | 0.039911 | - | - | - | - |
| IGHGP | 1.92 | 0.012563 | - | - | - | - |
| IGHM | 1.61 | 0.069491 | - | - | - | - |
| IGKV1-27 | 2.62 | 0.006431 | - | - | - | - |
| IGKV1-33 | 1.89 | 0.078814 | - | - | - | - |
| IGKV1-5 | 1.74 | 0.0353 | - | - | - | - |
| IGKV1-9 | 2.09 | 0.044008 | - | - | - | - |
| IGKV1D-33 | 1.88 | 0.080532 | - | - | - | - |
| IGKV3-11 | 2.84 | 8.04E-05 | - | - | - | - |
| IGKV3-20 | 2.19 | 0.001857 | - | - | - | - |
| IGKV3D-11 | 2.72 | 0.000307 | - | - | - | - |
| IGKV3D-20 | 2.00 | 0.011692 | - | - | - | - |
| IGKV4-1 | 1.75 | 0.020594 | - | - | - | - |
| IGLC1 | 2.57 | 0.000269 | - | - | - | - |
| IGLC2 | 2.07 | 0.002911 | - | - | - | - |
| IGLC3 | 2.11 | 0.001998 | - | - | - | - |
| IGLV10-54 | 3.51 | 0.003325 | - | - | - | - |
| IGLV1-40 | 2.54 | 0.034661 | - | - | - | - |
| IGLV1-44 | 2.12 | 0.026259 | - | - | - | - |
| IGLV1-47 | 2.34 | 0.011201 | - | - | - | - |
| IGLV2-11 | 2.04 | 0.038051 | - | - | - | - |
| IGLV2-14 | 2.73 | 9.05E-05 | - | - | - | - |
| IGLV2-23 | 2.69 | 0.000586 | - | - | - | - |
| IGLV2-8 | 2.51 | 0.001849 | - | - | - | - |
| IGLV3-10 | 2.61 | 0.004748 | - | - | - | - |
| IGLV4-69 | 2.96 | 0.020833 | - | - | - | - |
| MIF | 4.02 | 0.003322 | - | - | - | - |
| MRPL41 | 2.89 | 0.0031 | - | - | - | - |
| MTRNR2L9 | 9.37 | 0.000471 | - | - | - | - |
| NDUFA13 | 4.52 | 0.000258 | - | - | - | - |
| NDUFA7 | 3.90 | 7.69E-06 | - | - | - | - |
| NDUFB11 | 2.62 | 0.008032 | - | - | - | - |
| NDUFB7 | 2.33 | 0.007188 | - | - | - | - |
| NDUFC2 | 2.15 | 0.082279 | - | - | - | - |
| PRDX5 | 3.17 | 0.026487 | - | - | - | - |
| S100A11 | 2.47 | 0.000436 | - | - | - | - |
| S100A9 | 2.22 | 0.07823 | - | - | - | - |
| SOD1 | 3.36 | 0.000162 | - | - | - | - |
| TMSB4Y | 7.71 | 0.06267 | - | - | - | - |
| TREML1 | 2.17 | 0.033541 | - | - | - | - |
| UQCR10 | 1.88 | 0.032266 | - | - | - | - |
| UQCR11 | 2.55 | 0.0001 | - | - | - | - |
| USMG5 | 3.45 | 0.000663 | - | - | - | - |
| CCR4 | - | - | 1.37 | 0.003709 | - | - |
| CCR7 | - | - | 1.13 | 0.01804 | - | - |
| CD2 | - | - | 1.35 | 0.046277 | - | - |
| CD69 | - | - | 1.64 | 0.00643 | - | - |
| CD86 | - | - | 1.61 | 0.089965 | - | - |
| CD8A | - | - | 1.83 | 0.07247 | - | - |
| CXCL3 | - | - | 4.21 | 0.029293 | - | - |
| CYCS | - | - | 1.81 | 0.01096 | - | - |
| HLA-DQA2 | - | - | 2.12 | 7E-07 | - | - |
| HLA-DRA | - | - | 1.32 | 0.068317 | - | - |
| ICOS | - | - | 1.69 | 0.000649 | - | - |
| IFNGR1 | - | - | 1.52 | 0.031605 | - | - |
| IL3RA | - | - | 1.88 | 0.031753 | - | - |
| NDUFB1 | - | - | 1.14 | 0.098018 | - | - |
| P2RY10 | - | - | 1.50 | 0.04148 | - | - |
| PHLDA1 | - | - | 2.16 | 0.000747 | - | - |
| PRKCQ | - | - | 1.16 | 0.052172 | - | - |
| PSMD7 | - | - | 1.30 | 0.090011 | - | - |
| PSMG2 | - | - | 1.09 | 0.095778 | - | - |
| RPS6 | - | - | 1.53 | 0.017865 | - | - |
| SERPINB2 | - | - | 4.19 | 7.36E-06 | - | - |
| SUMO2 | - | - | 1.04 | 0.061377 | - | - |
| TNF | - | - | 1.68 | 0.008992 | - | - |
| TRIM38 | - | - | 1.17 | 0.023984 | - | - |
| UBA2 | - | - | 1.11 | 0.044524 | - | - |
| UBA52 | - | - | 1.13 | 0.051503 | - | - |
| UBB | - | - | 1.69 | 0.002375 | - | - |
| USP12 | - | - | 1.08 | 0.04077 | - | - |
| XCL1 | - | - | 1.91 | 0.009897 | - | - |
| ZNF165 | - | - | 2.97 | 0.001596 | - | - |
| C1QC | - | - | - | - | 2.43 | 0.072234 |
| C2 | - | - | - | - | 2.44 | 0.025012 |
| CTSG | - | - | - | - | 6.77 | 0.038889 |
| CXCL16 | - | - | - | - | 1.86 | 0.00065 |
| CYP4F2 | - | - | - | - | 4.57 | 0.011431 |
| EDN1 | - | - | - | - | 2.83 | 0.031754 |
| FFAR3 | - | - | - | - | 3.79 | 0.035412 |
| IFI30 | - | - | - | - | 1.86 | 0.011182 |
| IGKV2-24 | - | - | - | - | 2.40 | 0.004182 |
| IRAK3 | - | - | - | - | 1.72 | 0.065715 |
| LILRA3 | - | - | - | - | 1.43 | 0.035777 |
| LILRB4 | - | - | - | - | 1.71 | 0.069579 |
| NDUFS5 | - | - | - | - | 1.47 | 0.058366 |
| RNASE3 | - | - | - | - | 2.20 | 0.065386 |
| S100A12 | - | - | - | - | 1.88 | 0.014137 |
| SERPINE1 | - | - | - | - | 3.27 | 0.004158 |
| SIGLEC12 | - | - | - | - | 3.09 | 0.006751 |
| TNFSF9 | - | - | - | - | 1.89 | 0.093974 |
