# Supplementary material for: The expression patterns of immune response genes in the Peripheral Blood Mononuclear cells of pregnant women presenting with subclinical or clinical HEV infection are different and trimester-dependent: A whole transcriptome analysis
Source: PLoS One. 2020 Feb 3;15(2):e0228068. doi: 10.1371/journal.pone.0228068 (PMC6996850; doi:10.1371/journal.pone.0228068)
Supplement: S6 Table — (DOCX) [file pone.0228068.s008.docx]

**Significantly altered genes in acute and subclinical HEV infections in pregnant women in the 2^nd^ trimester with pair-wise comparisons done with non-pregnant healthy controls**

**Table S8- List of up-regulated genes:**

| **Gene short name** | **PR-2-acute** | | **PR-2-SC** | |
| --- | --- | --- | --- | --- |
|  | **Fold change** | **Q value** | **Fold change** | **Q value** |
| AZU1 | 2.68 | 0.044306 | 2.60 | 0.012252 |
| BCL2A1 | 3.20 | 0 | 2.08 | 0 |
| BPI | 3.50 | 5.99E-10 | 2.13 | 1.42E-07 |
| CAMP | 4.64 | 2.52E-12 | 3.15 | 6.58E-08 |
| CCL3 | 2.80 | 1.36E-07 | 4.33 | 0 |
| CCL3L1 | 3.10 | 0.009538 | 5.61 | 4.06E-12 |
| CCL3L3 | 2.70 | 2.16E-05 | 4.84 | 0 |
| CCL4 | 1.56 | 0.00355 | 2.67 | 0 |
| CCR1 | 1.90 | 1.9E-06 | 1.16 | 6.86E-08 |
| CCR7 | 1.13 | 0.01804 | 1.31 | 1.05E-10 |
| CCRL2 | 2.98 | 9.27E-08 | 2.79 | 0 |
| CD1D | 1.41 | 0.005909 | 1.22 | 6.97E-07 |
| CD69 | 1.64 | 0.00643 | 1.26 | 2.71E-08 |
| CD83 | 1.84 | 1.47E-06 | 1.67 | 0 |
| CD8A | 1.83 | 0.07247 | 1.02 | 0.048273 |
| CEACAM6 | 5.98 | 0 | 4.51 | 2.67E-13 |
| CEACAM8 | 4.98 | 0 | 3.60 | 0 |
| CEBPB | 1.58 | 0.00034 | 1.68 | 0 |
| CRISP3 | 5.99 | 0 | 4.10 | 9.38E-14 |
| CXCL2 | 2.33 | 0.021632 | 2.81 | 2.33E-09 |
| CXCL3 | 4.21 | 0.029293 | 4.33 | 0.004301 |
| CXCR4 | 1.90 | 0.001033 | 1.17 | 1.23E-05 |
| DDIT3 | 3.41 | 0.024712 | 2.57 | 0.093281 |
| DDIT4 | 2.46 | 8.12E-05 | 1.95 | 3.51E-10 |
| DEFA1 | 5.27 | 0 | 3.79 | 0 |
| DEFA1B | 5.19 | 9.38E-14 | 3.89 | 7.53E-10 |
| DEFA3 | 4.92 | 4.35E-13 | 3.86 | 0 |
| DEFA4 | 3.99 | 2.04E-11 | 3.02 | 1.2E-10 |
| DUSP10 | 2.68 | 0.000102 | 2.52 | 7.84E-10 |
| ELANE | 3.98 | 0.001116 | 3.71 | 0.000429 |
| FFAR2 | 3.75 | 1.82E-13 | 2.98 | 0 |
| G0S2 | 2.99 | 2.67E-11 | 2.14 | 2.97E-12 |
| GADD45B | 1.83 | 7.92E-05 | 1.74 | 2.66E-11 |
| HSPA1A | 3.66 | 0 | 3.95 | 0 |
| HSPA1B | 4.47 | 0 | 4.80 | 0 |
| ICAM1 | 1.97 | 0.000449 | 1.46 | 2.79E-10 |
| IER3 | 2.22 | 7.41E-05 | 2.50 | 6.71E-11 |
| IER5 | 1.33 | 0.022026 | 1.51 | 2.52E-11 |
| IFI27 | 4.06 | 0.083373 | 3.46 | 0.076869 |
| IFNG | 3.65 | 3.59E-07 | 2.17 | 0.001662 |
| IL1B | 3.23 | 1.43E-07 | 5.38 | 0 |
| IL3RA | 1.88 | 0.031753 | 1.15 | 0.002146 |
| IL8 | 4.24 | 5.43E-12 | 2.94 | 0 |
| JUN | 3.97 | 0 | 4.46 | 0 |
| LGALS3 | 2.31 | 0.002696 | 1.67 | 8.26E-07 |
| LILRA5 | 1.19 | 0.072473 | 1.00 | 0.00029 |
| MMP8 | 6.03 | 1.57E-07 | 4.53 | 2.01E-09 |
| MMP9 | 4.43 | 3.73E-07 | 4.37 | 9.26E-10 |
| NFKBIA | 1.88 | 0.022551 | 3.21 | 0 |
| NR4A3 | 1.81 | 0.031605 | 2.04 | 4.27E-05 |
| PGLYRP1 | 4.85 | 6.92E-07 | 3.90 | 8.68E-06 |
| PHLDA1 | 2.16 | 0.000747 | 1.90 | 3.29E-06 |
| PI3 | 8.25 | 9.38E-14 | 3.70 | 0.004901 |
| PLAU | 5.52 | 0.063655 | 6.62 | 4.97E-06 |
| PLAUR | 2.04 | 1.21E-06 | 1.28 | 2.12E-07 |
| PTGES | 4.20 | 1.63E-09 | 4.75 | 0 |
| REL | 1.67 | 3.14E-05 | 1.54 | 9.38E-14 |
| RIPK2 | 2.13 | 0.000862 | 1.43 | 0.000261 |
| SLPI | 5.77 | 1.69E-08 | 3.81 | 0.000207 |
| TGM2 | 3.78 | 2.34E-05 | 3.07 | 2.65E-05 |
| THBD | 2.44 | 4.29E-07 | 1.27 | 0.000722 |
| TNF | 1.68 | 0.008992 | 4.82 | 0 |
| CCR4 | 1.37 | 0.003709 | - | - |
| CD109 | 1.92 | 0.035403 | - | - |
| CD2 | 1.35 | 0.046277 | - | - |
| CD300A | 1.24 | 0.099686 | - | - |
| CD300LB | 1.10 | 0.087767 | - | - |
| CD48 | 1.81 | 1.96E-05 | - | - |
| CD86 | 1.61 | 0.089965 | - | - |
| CEACAM3 | 3.13 | 0.026228 | - | - |
| CEACAM5 | 4.10 | 0.001535 | - | - |
| CHAC1 | 3.40 | 0.0506 | - | - |
| CXCR1 | 2.26 | 0.000159 | - | - |
| DHX40 | 1.55 | 0.000233 | - | - |
| GPI | 1.21 | 0.011944 | - | - |
| HBXIP | 1.61 | 0.040348 | - | - |
| HLA-DQA2 | 2.12 | 7E-07 | - | - |
| HLA-DRA | 1.32 | 0.068317 | - | - |
| ICOS | 1.69 | 0.000649 | - | - |
| IFNGR1 | 1.52 | 0.031605 | - | - |
| IRAK2 | 1.34 | 0.027933 | - | - |
| JUND | 1.50 | 0.000652 | - | - |
| KRT23 | 3.73 | 9.5E-08 | - | - |
| LEP | 8.96 | 0.000435 | - | - |
| MARCKS | 1.33 | 0.003836 | - | - |
| MYL4 | 2.55 | 0.041224 | - | - |
| OLR1 | 4.52 | 0.031751 | - | - |
| P2RY10 | 1.50 | 0.04148 | - | - |
| PF4 | 1.27 | 0.049382 | - | - |
| POMP | 1.60 | 0.006659 | - | - |
| PRKCQ | 1.16 | 0.052172 | - | - |
| PSMG2 | 1.09 | 0.095778 | - | - |
| RPS19 | 1.71 | 0.00445 | - | - |
| S100A8 | 1.35 | 0.005585 | - | - |
| SERPINB2 | 4.19 | 7.36E-06 | - | - |
| TLR2 | 1.26 | 0.012942 | - | - |
| TNFAIP6 | 4.85 | 9.92E-08 | - | - |
| TNFRSF10D | 1.48 | 0.033428 | - | - |
| TREM1 | 1.21 | 0.022294 | - | - |
| TRIM38 | 1.17 | 0.023984 | - | - |
| UBA2 | 1.11 | 0.044524 | - | - |
| UBA52 | 1.13 | 0.051503 | - | - |
| UBAP1 | 1.43 | 0.003374 | - | - |
| UBB | 1.69 | 0.002375 | - | - |
| UQCRB | 1.26 | 0.013476 | - | - |
| VSIG4 | 1.87 | 0.02433 | - | - |
| CCL2 | - | - | 2.34 | 0.029504 |
| CCL4L1 | - | - | 4.36 | 1.23E-12 |
| CCL4L2 | - | - | 3.88 | 0 |
| CD28 | - | - | 1.01 | 0.034506 |
| CD5 | - | - | 1.35 | 0.006827 |
| CSF2 | - | - | 1.8+308 | 0.039001 |
| DNASE2 | - | - | 1.52 | 0.000173 |
| EDN1 | - | - | 3.20 | 2.43E-05 |
| GPX1 | - | - | 1.04 | 0.001082 |
| GZMM | - | - | 1.23 | 0.019421 |
| HLA-DPA1 | - | - | 1.26 | 0.012483 |
| HLA-DQB1 | - | - | 2.35 | 2.29E-10 |
| HLA-DQB2 | - | - | 2.24 | 7.32E-08 |
| HMOX1 | - | - | 1.49 | 0.000109 |
| ID3 | - | - | 1.30 | 0.021809 |
| IER5L | - | - | 2.17 | 0.095737 |
| IGLV10-54 | - | - | 2.18 | 0.011367 |
| ISG15 | - | - | 1.01 | 0.00687 |
| KRT1 | - | - | 1.12 | 0.09072 |
| NDUFB7 | - | - | 1.14 | 0.048716 |
| NFKBID | - | - | 1.30 | 0.039194 |
| NFKBIZ | - | - | 1.81 | 8.5E-06 |
| NR4A1 | - | - | 2.27 | 0.001242 |
| P2RY2 | - | - | 2.29 | 2.41E-07 |
| PIK3IP1 | - | - | 1.01 | 0.044988 |
| PIK3R1 | - | - | 1.19 | 0.038271 |
| PTGS2 | - | - | 1.34 | 5.83E-06 |
| RHOB | - | - | 1.47 | 1.94E-10 |
| SELENBP1 | - | - | 3.93 | 0.000502 |
| SPI1 | - | - | 1.14 | 3.73E-07 |
| TGFB1 | - | - | 1.14 | 0.008108 |
| TNFAIP3 | - | - | 1.93 | 5.37E-09 |
| TNFRSF13C | - | - | 2.11 | 8.23E-09 |
| TNFSF8 | - | - | 1.04 | 0.019033 |
| TUBA4A | - | - | 1.49 | 0.001942 |
| UCA1 | - | - | 2.70 | 0.008591 |
| USP36 | - | - | 1.73 | 3.56E-08 |
| VEGFA | - | - | 1.87 | 0.017508 |
