# Supplementary material for: The expression patterns of immune response genes in the Peripheral Blood Mononuclear cells of pregnant women presenting with subclinical or clinical HEV infection are different and trimester-dependent: A whole transcriptome analysis
Source: PLoS One. 2020 Feb 3;15(2):e0228068. doi: 10.1371/journal.pone.0228068 (PMC6996850; doi:10.1371/journal.pone.0228068)
Supplement: S7 Table — (DOCX) [file pone.0228068.s009.docx]

**Table S9- List of down-regulated genes:**

| **Gene short name** | **PR-2-acute** | | **PR-2-SC** | |
| --- | --- | --- | --- | --- |
|  | **Fold change** | **Q value** | **Fold change** | **Q value** |
| SIGLEC1 | -1.85 | 0.069299 | - | - |
| SOCS3 | -1.24 | 0.014824 | - | - |
| ACTG1 | - | - | -1.33 | 2.36E-08 |
| CCR2 | - | - | -1.06 | 0.002187 |
| CD160 | - | - | -1.68 | 0.00027 |
| CD180 | - | - | -1.18 | 4.88E-08 |
| CD1A | - | - | -2.00 | 0.031871 |
| CD244 | - | - | -1.42 | 2.07E-07 |
| COX5B | - | - | -1.29 | 0.010339 |
| CPEB3 | - | - | -1.30 | 0.003389 |
| CX3CR1 | - | - | -2.64 | 0 |
| CXCL10 | - | - | -1.01 | 0.051865 |
| CXCL5 | - | - | -2.19 | 0 |
| DDX58 | - | - | -1.17 | 1.28E-05 |
| DEAF1 | - | - | -1.80 | 0.057944 |
| GZMA | - | - | -1.07 | 1.54E-05 |
| IGHV3-21 | - | - | -1.29 | 0.003984 |
| IGHV3-7 | - | - | -1.38 | 0.002292 |
| IGHV4-59 | - | - | -1.16 | 0.01197 |
| IGJ | - | - | -4.53 | 0 |
| IGKC | - | - | -2.07 | 1.94E-10 |
| IGKV1-12 | - | - | -2.31 | 3.19E-12 |
| IGKV1-39 | - | - | -1.71 | 7.44E-08 |
| IGKV1-6 | - | - | -1.91 | 0.002233 |
| IGKV1D-13 | - | - | -1.45 | 0.031922 |
| IGKV2-28 | - | - | -1.03 | 0.034354 |
| IGKV2D-28 | - | - | -2.39 | 0.018387 |
| IGKV2D-29 | - | - | -1.80 | 0.058649 |
| IGKV3D-15 | - | - | -1.01 | 0.060632 |
| IGKV4-1 | - | - | -1.45 | 3.34E-06 |
| IGLV2-8 | - | - | -1.41 | 0.059958 |
| IGLV3-25 | - | - | -1.56 | 0.018898 |
| IGLV7-46 | - | - | -2.61 | 0.003083 |
| IL32 | - | - | -1.03 | 3.37E-05 |
| LY86 | - | - | -1.01 | 0.000283 |
| MAP2K6 | - | - | -1.03 | 0.01308 |
| NDUFA8 | - | - | -1.60 | 0.00023 |
| NDUFAF1 | - | - | -1.96 | 9.85E-06 |
| NLRC4 | - | - | -1.54 | 5.75E-06 |
| NPTXR | - | - | -1.07 | 0.073227 |
| PRDX1 | - | - | -1.23 | 1.42E-06 |
| PRDX3 | - | - | -1.20 | 4.21E-05 |
| RNASE2 | - | - | -1.31 | 2.06E-06 |
| TLR10 | - | - | -2.37 | 0.000428 |
| TNFAIP8L2 | - | - | -1.36 | 9.76E-05 |
| TNFRSF17 | - | - | -2.57 | 0.001391 |
| TNFSF10 | - | - | -1.87 | 0 |
| TNFSF13 | - | - | -1.31 | 0.000654 |
| UBE2D1 | - | - | -1.24 | 1.24E-07 |
| UBE3B | - | - | -1.02 | 0.05293 |
| USP18 | - | - | -1.17 | 0.082952 |
