# Supplementary material for: The expression patterns of immune response genes in the Peripheral Blood Mononuclear cells of pregnant women presenting with subclinical or clinical HEV infection are different and trimester-dependent: A whole transcriptome analysis
Source: PLoS One. 2020 Feb 3;15(2):e0228068. doi: 10.1371/journal.pone.0228068 (PMC6996850; doi:10.1371/journal.pone.0228068)
Supplement: S8 Table — (DOCX) [file pone.0228068.s010.docx]

**Significantly altered genes in acute and subclinical HEV infections in pregnant women in the 3rd trimester with pair-wise comparisons done with non-pregnant healthy controls**

**Table S10- List of up-regulated genes:**

| **Gene short name** | **PR-3-acute** | | **PR-3-SC** | |
| --- | --- | --- | --- | --- |
|  | **Fold change** | **Q value** | **Fold change** | **Q value** |
| AZU1 | 3.97 | 0.000293 | 2.85 | 0.017408 |
| BCL2A1 | 3.69 | 0 | 2.42 | 3.33E-10 |
| BPI | 4.41 | 1.24E-10 | 2.55 | 3.33E-06 |
| C1QA | 2.80 | 0.084955 | 2.59 | 0.015836 |
| C1QB | 1.80 | 0.068305 | 1.84 | 0.005673 |
| C1QC | 2.43 | 0.072234 | 2.43 | 0.020809 |
| CAMP | 5.67 | 0 | 4.13 | 7.71E-10 |
| CCL3 | 3.17 | 1.52E-06 | 4.34 | 0 |
| CCL3L1 | 3.41 | 0.010754 | 5.32 | 0.090545 |
| CCL3L3 | 2.72 | 0.000519 | 4.45 | 9.02E-10 |
| CCR1 | 2.47 | 5.2E-07 | 1.75 | 3.75E-07 |
| CCRL2 | 3.82 | 0.000524 | 2.77 | 2.73E-06 |
| CD109 | 2.44 | 0.006203 | 2.16 | 0.059085 |
| CD1D | 1.79 | 0.001266 | 1.54 | 0.000111 |
| CD300LB | 1.82 | 0.000503 | 1.43 | 0.000605 |
| CD48 | 1.54 | 0.029152 | 1.66 | 1.44E-06 |
| CD83 | 1.54 | 0.004037 | 1.32 | 0.000405 |
| CEACAM5 | 4.92 | 0.000182 | 3.62 | 0.009847 |
| CEACAM6 | 6.95 | 0 | 5.22 | 2.67E-13 |
| CEACAM8 | 6.17 | 0 | 3.97 | 0 |
| CEBPB | 2.03 | 0.000397 | 2.25 | 3.92E-12 |
| CRISP3 | 7.05 | 0.085262 | 4.43 | 1.36E-09 |
| CXCL16 | 1.86 | 0.00065 | 1.07 | 0.040315 |
| CXCL2 | 3.17 | 0.001094 | 2.64 | 0.000661 |
| CXCR4 | 1.67 | 0.075246 | 1.62 | 0.00035 |
| DDIT3 | 3.44 | 0.062548 | 3.00 | 0.058462 |
| DDIT4 | 3.36 | 0.00523 | 3.15 | 1.15E-10 |
| DEFA1 | 6.23 | 0 | 5.13 | 0 |
| DEFA1B | 6.16 | 1.64E-10 | 5.10 | 5.78E-11 |
| DEFA3 | 6.24 | 2.67E-13 | 4.98 | 1.82E-13 |
| DEFA4 | 5.06 | 0 | 3.82 | 9.15E-11 |
| DNASE2 | 2.13 | 0.009798 | 2.37 | 2.14E-05 |
| DUSP10 | 2.70 | 0.00432 | 2.21 | 0.000637 |
| EDN1 | 2.83 | 0.031754 | 2.82 | 0.004709 |
| ELANE | 4.79 | 0.000111 | 4.26 | 0.00019 |
| FFAR2 | 4.03 | 9.38E-14 | 3.01 | 1.6E-10 |
| G0S2 | 3.14 | 1.12E-09 | 2.28 | 3.97E-07 |
| GADD45B | 2.28 | 0.000162 | 2.55 | 8.33E-12 |
| GPI | 2.42 | 2.58E-06 | 1.14 | 0.005085 |
| HSPA1A | 3.81 | 2.94E-10 | 4.03 | 0 |
| HSPA1B | 4.63 | 5.18E-10 | 5.18 | 0 |
| ICAM1 | 2.45 | 3.14E-05 | 1.97 | 5.16E-07 |
| IER3 | 3.08 | 2.34E-07 | 3.12 | 5.75E-11 |
| IER5 | 1.43 | 0.080709 | 1.60 | 0.000162 |
| IFI27 | 5.30 | 0.024672 | 5.96 | 6.66E-05 |
| IFI30 | 1.86 | 0.011182 | 1.24 | 0.005917 |
| IFNG | 2.94 | 0.001061 | 2.33 | 0.007319 |
| IL1B | 3.12 | 0.000355 | 4.23 | 1.82E-13 |
| IL8 | 5.50 | 4.12E-05 | 2.94 | 6.85E-10 |
| JUN | 4.19 | 9.97E-13 | 4.58 | 0 |
| JUND | 1.56 | 0.014111 | 1.51 | 5.82E-05 |
| LGALS3 | 2.40 | 0.014241 | 2.51 | 3.51E-07 |
| LILRA5 | 2.12 | 0.006223 | 1.40 | 0.011864 |
| MMP8 | 6.94 | 1.71E-08 | 4.70 | 6.65E-05 |
| MMP9 | 5.50 | 6.46E-11 | 2.91 | 0.004721 |
| NFKBIA | 2.07 | 0.04393 | 2.80 | 9.31E-08 |
| PGLYRP1 | 6.56 | 4.35E-13 | 3.65 | 0.00058 |
| PI3 | 6.94 | 1.14E-08 | 3.50 | 0.021 |
| PLAU | 5.85 | 0.074513 | 6.06 | 0.001254 |
| PLAUR | 2.33 | 0.000468 | 2.04 | 7.34E-08 |
| PTGES | 5.51 | 0 | 5.08 | 0 |
| REL | 1.58 | 0.00384 | 1.57 | 7.42E-06 |
| RIPK2 | 2.38 | 0.003632 | 1.54 | 0.018757 |
| RPS19 | 2.57 | 0.000545 | 1.78 | 0.00089 |
| SERPINE1 | 3.27 | 0.004158 | 3.69 | 0.000161 |
| SLPI | 5.68 | 1.3E-07 | 4.49 | 4.28E-05 |
| TGM2 | 4.82 | 5.35E-08 | 4.82 | 2.27E-10 |
| THBD | 3.55 | 8.39E-13 | 3.13 | 9.38E-14 |
| TREM1 | 1.73 | 0.002126 | 1.65 | 1.85E-05 |
| UBAP1 | 1.72 | 0.004768 | 1.00 | 0.033364 |
| VSIG4 | 2.11 | 0.02405 | 1.83 | 0.022487 |
| C2 | 2.44 | 0.025012 | - | - |
| CD300A | 1.70 | 0.070235 | - | - |
| CEACAM3 | 3.43 | 0.024488 | - | - |
| CEBPE | 3.70 | 0.000662 | - | - |
| CTSG | 6.77 | 0.038889 | - | - |
| CXCR1 | 2.78 | 5.38E-05 | - | - |
| CYP4F2 | 4.57 | 0.011431 | - | - |
| CYP4F3 | 3.90 | 0.001403 | - | - |
| CYP51P2 | 2.16 | 0.058965 | - | - |
| DHX40 | 1.72 | 0.015245 | - | - |
| FFAR3 | 3.79 | 0.035412 | - | - |
| IGHV6-1 | 1.79 | 0.094895 | - | - |
| IGKV2-24 | 2.40 | 0.004182 | - | - |
| IRAK2 | 1.52 | 0.032305 | - | - |
| IRAK3 | 1.72 | 0.065715 | - | - |
| KRT23 | 2.87 | 0.001987 | - | - |
| LEP | 10.55 | 1.08E-05 | - | - |
| LILRA3 | 1.43 | 0.035777 | - | - |
| LILRB4 | 1.71 | 0.069579 | - | - |
| POMP | 1.60 | 0.037506 | - | - |
| S100A12 | 1.88 | 0.014137 | - | - |
| S100A8 | 2.24 | 0.000297 | - | - |
| SIGLEC16 | 1.65 | 0.068361 | - | - |
| TLR2 | 1.83 | 0.000552 | - | - |
| TNFAIP6 | 5.32 | 5.4E-07 | - | - |
| TNFRSF10D | 1.55 | 0.039547 | - | - |
| TNFSF9 | 1.89 | 0.093974 | - | - |
| UQCRB | 1.36 | 0.091041 | - | - |
| ARID5A | - | - | 2.60 | 5.91E-05 |
| CCL2 | - | - | 3.30 | 0.001052 |
| CCL4 | - | - | 1.96 | 1.57E-05 |
| CCL4L2 | - | - | 2.93 | 0.093641 |
| CCR4 | - | - | 1.11 | 0.017917 |
| CCR7 | - | - | 1.87 | 2.8E-08 |
| CD68 | - | - | 1.14 | 0.011396 |
| CD69 | - | - | 1.01 | 0.099255 |
| CD8A | - | - | 1.26 | 0.045086 |
| CD9 | - | - | 1.92 | 0.011615 |
| CXCL3 | - | - | 4.42 | 0.015681 |
| DKK3 | - | - | 3.55 | 0.034817 |
| DUSP6 | - | - | 1.46 | 0.046663 |
| FCER1G | - | - | 1.15 | 0.006053 |
| GP9 | - | - | 2.32 | 0.000104 |
| GPX1 | - | - | 2.14 | 2.25E-05 |
| GZMM | - | - | 1.68 | 0.015484 |
| HBXIP | - | - | 1.40 | 0.043233 |
| HMOX1 | - | - | 2.05 | 0.001223 |
| HLA-DPA1 | - | - | 1.98 | 0.070669 |
| HLA-DQA2 | - | - | 1.33 | 0.002272 |
| HLA-DQB1 | - | - | 2.09 | 0.001457 |
| HLA-DQB2 | - | - | 1.58 | 0.011284 |
| HLA-DRA | - | - | 1.21 | 0.022779 |
| HLA-DRB1 | - | - | 1.11 | 0.006058 |
| IER2 | - | - | 1.79 | 3.32E-06 |
| IER5L | - | - | 3.00 | 0.011385 |
| IFI6 | - | - | 1.22 | 0.010519 |
| IFNGR1 | - | - | 1.15 | 0.056438 |
| IL23A | - | - | 1.74 | 0.039196 |
| IL3RA | - | - | 1.39 | 0.017936 |
| INSIG1 | - | - | 2.10 | 0.001341 |
| ISG15 | - | - | 1.26 | 0.035113 |
| MYL4 | - | - | 3.15 | 0.001365 |
| OASL | - | - | 1.69 | 0.006632 |
| P2RY1 | - | - | 1.88 | 0.001476 |
| P2RY2 | - | - | 2.44 | 8.59E-05 |
| PF4 | - | - | 2.02 | 1.11E-07 |
| PHLDA1 | - | - | 2.27 | 0.021376 |
| PRDX5 | - | - | 2.30 | 0.00834 |
| RHOB | - | - | 1.42 | 0.000808 |
| S100A6 | - | - | 1.42 | 0.00012 |
| SELENBP1 | - | - | 5.31 | 6.36E-05 |
| SPI1 | - | - | 1.18 | 0.017974 |
| TNF | - | - | 4.23 | 0 |
| TNFAIP3 | - | - | 1.59 | 0.04462 |
| TNFRSF12A | - | - | 2.87 | 0.027734 |
| TNFRSF13C | - | - | 1.89 | 0.001085 |
| TNFSF8 | - | - | 1.52 | 0.045881 |
| TUBA4A | - | - | 2.15 | 0.009014 |
| UBA52 | - | - | 1.16 | 0.005662 |
| UBB | - | - | 1.83 | 9.27E-05 |
| UBE2L6 | - | - | 1.43 | 0.007474 |
| UQCRH | - | - | 1.14 | 0.040324 |
| USF2 | - | - | 1.25 | 0.085756 |
| USP36 | - | - | 1.66 | 0.002522 |
