# Supplementary material for: The expression patterns of immune response genes in the Peripheral Blood Mononuclear cells of pregnant women presenting with subclinical or clinical HEV infection are different and trimester-dependent: A whole transcriptome analysis
Source: PLoS One. 2020 Feb 3;15(2):e0228068. doi: 10.1371/journal.pone.0228068 (PMC6996850; doi:10.1371/journal.pone.0228068)
Supplement: S9 Table — (DOCX) [file pone.0228068.s011.docx]

**Table S11-List of down-regulated genes:**

| **Gene short name** | **PR-3-acute** | | **PR-3-SC** | |
| --- | --- | --- | --- | --- |
|  | **Fold change** | **Q value** | **Fold change** | **Q value** |
| CD160 | - | - | -1.94 | 0.033718 |
| CD1C | - | - | -1.20 | 0.086762 |
| CX3CR1 | - | - | -3.21 | 0.000372 |
| DDX58 | - | - | -1.40 | 0.010321 |
| IGHA1 | - | - | -1.26 | 0.00834 |
| IGHA2 | - | - | -1.68 | 0.001065 |
| IGHV3-21 | - | - | -1.93 | 0.004592 |
| IGJ | - | - | -3.95 | 6.55E-05 |
| IGKC | - | - | -1.16 | 0.070899 |
| IGKV1-12 | - | - | -1.98 | 0.000667 |
| IGKV1-39 | - | - | -1.66 | 0.005313 |
| IGKV1D-16 | - | - | -1.60 | 0.093603 |
| IGKV2-28 | - | - | -1.42 | 0.086284 |
| IGKV4-1 | - | - | -1.29 | 0.052991 |
| IGLV3-1 | - | - | -1.47 | 0.003889 |
| IGLV3-25 | - | - | -2.10 | 0.018379 |
| TNFSF10 | - | - | -1.25 | 0.069976 |
| TRAM2 | - | - | -1.55 | 0.006069 |
