# Supplementary material for: The expression patterns of immune response genes in the Peripheral Blood Mononuclear cells of pregnant women presenting with subclinical or clinical HEV infection are different and trimester-dependent: A whole transcriptome analysis
Source: PLoS One. 2020 Feb 3;15(2):e0228068. doi: 10.1371/journal.pone.0228068 (PMC6996850; doi:10.1371/journal.pone.0228068)
Supplement: S10 Table — (DOCX) [file pone.0228068.s012.docx]

**Significantly up-regulated genes in acute NPR and Pregnant patients with pair-wise comparison done with respective healthy pregnant controls**

**Table S12 List of up-regulated genes:**

| **Gene short name** | **NPR-acute** | | **PR-2-acute** | | **PR-3-acute** | |
| --- | --- | --- | --- | --- | --- | --- |
|  | **Fold change** | **Q value** | **Fold change** | **Q value** | **Fold change** | **Q value** |
| CEACAM6 | 3.50 | 0.024637 | 1.71 | 0.047286 | 3.36 | 2.54E-06 |
| IGHG3 | 1.72 | 0.031085 | 1.63 | 0.039001 | 2.35 | 0.002367 |
| IGHV6-1 | 2.42 | 0.015893 | 3.29 | 0.002869 | 3.89 | 0.001972 |
| PI3 | 6.35 | 2.41E-05 | 3.42 | 2.5E-06 | 2.96 | 0.00439 |
| ARHGDIA | - | - | 1.60 | 0.010745 | 2.22 | 0.000824 |
| CD180 | - | - | 1.75 | 0.008552 | 2.35 | 0.000575 |
| CEACAM8 | - | - | 2.21 | 0.000219 | 3.74 | 1.05E-08 |
| CXCR1 | - | - | 3.23 | 3.08E-05 | 3.37 | 2.82E-05 |
| DEFA4 | - | - | 1.62 | 0.089063 | 2.97 | 6.19E-05 |
| DUSP22 | - | - | 1.67 | 0.077381 | 1.86 | 0.081514 |
| IGHV1-18 | - | - | 2.20 | 0.053091 | 2.29 | 0.087608 |
| IGHV1-69 | - | - | 2.28 | 0.041935 | 3.10 | 0.029514 |
| IGHV3-11 | - | - | 2.20 | 0.067041 | 3.25 | 0.004361 |
| IGHV3-21 | - | - | 2.85 | 0.003832 | 2.27 | 0.064839 |
| IGHV3-23 | - | - | 2.74 | 6.41E-05 | 3.52 | 1.87E-06 |
| IGHV3-30 | - | - | 2.55 | 0.002429 | 2.60 | 0.005572 |
| IGHV3-33 | - | - | 2.68 | 0.002023 | 2.78 | 0.004549 |
| IGHV3-48 | - | - | 2.20 | 0.065367 | 3.25 | 0.002099 |
| IGHV3-49 | - | - | 2.54 | 0.071557 | 3.76 | 0.048892 |
| IGHV3-53 | - | - | 2.80 | 0.024548 | 2.94 | 0.069807 |
| IGHV3-72 | - | - | 3.31 | 0.025208 | 3.66 | 0.013303 |
| IGJ | - | - | 3.48 | 0.040866 | 3.68 | 0.038475 |
| IGKV1-12 | - | - | 2.79 | 0.003736 | 3.43 | 0.000354 |
| IGKV1-16 | - | - | 2.22 | 0.061066 | 3.75 | 0.000986 |
| IGKV1-39 | - | - | 2.41 | 0.003668 | 2.76 | 0.001647 |
| IGKV1D-12 | - | - | 2.60 | 0.050902 | 2.84 | 0.05904 |
| IGKV1D-16 | - | - | 2.21 | 0.097624 | 3.78 | 0.003742 |
| IGKV2-28 | - | - | 2.20 | 0.031119 | 3.02 | 0.001188 |
| IGLV3-1 | - | - | 4.00 | 1.46E-08 | 1.90 | 0.047045 |
| LEP | - | - | 2.92 | 0.000334 | 3.31 | 1.53E-05 |
| MEFV | - | - | 2.24 | 0.01121 | 2.68 | 0.005444 |
| MMP9 | - | - | 3.59 | 0.052384 | 4.41 | 0.000368 |
| MPO | - | - | 2.44 | 0.001328 | 3.67 | 2.93E-08 |
| PRKCSH | - | - | 1.57 | 0.010091 | 2.24 | 0.00067 |
| TICAM1 | - | - | 1.87 | 0.014249 | 2.57 | 0.001001 |
| TLR8 | - | - | 1.59 | 0.008444 | 1.99 | 0.004608 |
| TNFAIP6 | - | - | 2.87 | 0.010293 | 3.58 | 0.0004 |
| TNFSF10 | - | - | 2.08 | 0.095435 | 2.39 | 0.02906 |
| UQCRB | - | - | 2.30 | 0.000213 | 2.35 | 0.003273 |
| VAV1 | - | - | 1.54 | 0.032939 | 1.97 | 0.005779 |
| WDTC1 | - | - | 2.48 | 0.000793 | 3.12 | 0.000102 |
| AQP3 | 2.85 | 0.034608 | - | - | - | - |
| BCL2A1 | 2.66 | 0.000111 | - | - | - | - |
| C1QA | 3.92 | 0.000384 | - | - | - | - |
| C1QB | 2.39 | 0.006934 | - | - | - | - |
| CAMP | 3.77 | 0.013601 | - | - | - | - |
| CCL2 | 3.46 | 0.018989 | - | - | - | - |
| CCL3 | 2.70 | 0.014008 | - | - | - | - |
| CCL4 | 2.07 | 0.088549 | - | - | - | - |
| CCR1 | 1.87 | 0.04325 | - | - | - | - |
| CD1D | 1.87 | 0.008172 | - | - | - | - |
| CD48 | 2.21 | 0.005939 | - | - | - | - |
| CEBPB | 1.86 | 0.006659 | - | - | - | - |
| CEBPE | 3.06 | 0.098163 | - | - | - | - |
| CMTM2 | 3.35 | 0.081737 | - | - | - | - |
| CRISP3 | 4.53 | 0.056273 | - | - | - | - |
| CST7 | 1.79 | 0.018305 | - | - | - | - |
| DEFA1 | 3.50 | 0.024374 | - | - | - | - |
| DNASE2 | 3.13 | 0.000369 | - | - | - | - |
| DUSP4 | 2.15 | 0.076444 | - | - | - | - |
| FCER1G | 1.94 | 0.033222 | - | - | - | - |
| FFAR2 | 3.78 | 7.01E-08 | - | - | - | - |
| FIS1 | 3.60 | 0.00927 | - | - | - | - |
| G0S2 | 2.34 | 0.001842 | - | - | - | - |
| GADD45B | 2.16 | 0.001895 | - | - | - | - |
| GP9 | 2.53 | 0.004525 | - | - | - | - |
| GPX1 | 3.48 | 0.006821 | - | - | - | - |
| GZMA | 1.60 | 0.045831 | - | - | - | - |
| GZMM | 2.14 | 0.021807 | - | - | - | - |
| HSPA1A | 2.31 | 0.027211 | - | - | - | - |
| HSPA1B | 2.96 | 0.006277 | - | - | - | - |
| ICAM3 | 1.43 | 0.081872 | - | - | - | - |
| ID1 | 4.79 | 0.090025 | - | - | - | - |
| IER3 | 3.06 | 3.85E-05 | - | - | - | - |
| IER5L | 3.16 | 0.039663 | - | - | - | - |
| IFI27 | 5.76 | 0.019859 | - | - | - | - |
| IGKV1-27 | 2.62 | 0.006431 | - | - | - | - |
| IGKV1-33 | 1.89 | 0.078814 | - | - | - | - |
| IGKV1D-33 | 1.88 | 0.080532 | - | - | - | - |
| IGKV3-11 | 2.84 | 8.04E-05 | - | - | - | - |
| IGKV3-20 | 2.19 | 0.001857 | - | - | - | - |
| IGKV3D-11 | 2.72 | 0.000307 | - | - | - | - |
| IGKV3D-20 | 2.00 | 0.011692 | - | - | - | - |
| IGLC1 | 2.57 | 0.000269 | - | - | - | - |
| IGLV10-54 | 3.51 | 0.003325 | - | - | - | - |
| IGLV1-40 | 2.54 | 0.034661 | - | - | - | - |
| IGLV1-44 | 2.12 | 0.026259 | - | - | - | - |
| IGLV1-47 | 2.34 | 0.011201 | - | - | - | - |
| IGLV2-11 | 2.04 | 0.038051 | - | - | - | - |
| IGLV2-14 | 2.73 | 9.05E-05 | - | - | - | - |
| IGLV2-23 | 2.69 | 0.000586 | - | - | - | - |
| IGLV2-8 | 2.51 | 0.001849 | - | - | - | - |
| IGLV3-10 | 2.61 | 0.004748 | - | - | - | - |
| IGLV4-69 | 2.96 | 0.020833 | - | - | - | - |
| JUN | 4.21 | 2.35E-09 | - | - | - | - |
| JUND | 2.05 | 0.003574 | - | - | - | - |
| KRT23 | 3.92 | 0.000724 | - | - | - | - |
| MTRNR2L9 | 9.37 | 0.000471 | - | - | - | - |
| MYL4 | 4.11 | 0.035191 | - | - | - | - |
| NDUFA13 | 4.52 | 0.000258 | - | - | - | - |
| NDUFA7 | 3.90 | 7.69E-06 | - | - | - | - |
| NDUFB11 | 2.62 | 0.008032 | - | - | - | - |
| NDUFB7 | 2.33 | 0.007188 | - | - | - | - |
| NDUFC2 | 2.15 | 0.082279 | - | - | - | - |
| P2RY1 | 2.01 | 0.055753 | - | - | - | - |
| PF4 | 3.65 | 1.58E-09 | - | - | - | - |
| PLAUR | 2.58 | 0.000635 | - | - | - | - |
| PRDX5 | 3.17 | 0.026487 | - | - | - | - |
| PTGES | 4.72 | 1.95E-07 | - | - | - | - |
| RPS19 | 5.22 | 0 | - | - | - | - |
| S100A11 | 2.47 | 0.000436 | - | - | - | - |
| S100A8 | 2.28 | 0.023101 | - | - | - | - |
| S100A9 | 2.22 | 0.07823 | - | - | - | - |
| SOD1 | 3.36 | 0.000162 | - | - | - | - |
| TMSB4Y | 7.71 | 0.06267 | - | - | - | - |
| TREML1 | 2.17 | 0.033541 | - | - | - | - |
| UQCR10 | 1.88 | 0.032266 | - | - | - | - |
| UQCR11 | 2.55 | 0.0001 | - | - | - | - |
| USMG5 | 3.45 | 0.000663 | - | - | - | - |
| CLEC4E | - | - | 2.00 | 0.035527 | - | - |
| CXCL1 | - | - | 2.61 | 0.037048 | - | - |
| CXCL13 | - | - | Inf | 0.074389 | - | - |
| CXCR3 | - | - | 2.20 | 0.024832 | - | - |
| DDX58 | - | - | 1.88 | 0.033716 | - | - |
| DHX40 | - | - | 1.22 | 0.081451 | - | - |
| HTATSF1 | - | - | 3.60 | 0.006955 | - | - |
| IGHD | - | - | 2.14 | 0.000337 | - | - |
| IGHV4-31 | - | - | 2.39 | 0.082918 | - | - |
| IGHV4-34 | - | - | 1.87 | 0.087496 | - | - |
| IGLV6-57 | - | - | 2.92 | 0.020789 | - | - |
| IGLV8-61 | - | - | 2.59 | 0.011509 | - | - |
| IKBIP | - | - | 2.07 | 0.060061 | - | - |
| PRDX4 | - | - | 2.58 | 0.094596 | - | - |
| PSME3 | - | - | 1.87 | 0.024903 | - | - |
| SMAD2 | - | - | 1.23 | 0.089558 | - | - |
| SMAD4 | - | - | 1.93 | 0.054525 | - | - |
| TBX21 | - | - | 1.80 | 0.002712 | - | - |
| TNFRSF10D | - | - | 1.77 | 0.0299 | - | - |
| UBA2 | - | - | 1.64 | 0.014096 | - | - |
| USP1 | - | - | 1.57 | 0.009907 | - | - |
| VNN2 | - | - | 2.04 | 0.074411 | - | - |
| VSIG4 | - | - | 2.34 | 0.018627 | - | - |
| BPI | - | - | - | - | 3.70 | 0.00021 |
| CD300LB | - | - | - | - | 1.48 | 0.054427 |
| CYP27A1 | - | - | - | - | 2.03 | 0.075184 |
| CYP4F3 | - | - | - | - | 2.49 | 0.062394 |
| ELANE | - | - | - | - | 3.06 | 0.043815 |
| GPX4 | - | - | - | - | 2.09 | 0.000986 |
| IGHA1 | - | - | - | - | 2.70 | 0.000138 |
| IGHA2 | - | - | - | - | 2.44 | 0.001661 |
| IGHG2 | - | - | - | - | 2.06 | 0.010353 |
| IGHV1-2 | - | - | - | - | 2.30 | 0.083341 |
| IGHV3-7 | - | - | - | - | 2.52 | 0.035003 |
| IGHV3-74 | - | - | - | - | 2.96 | 0.066317 |
| IGHV3-9 | - | - | - | - | 2.57 | 0.072926 |
| IGHV4-39 | - | - | - | - | 2.97 | 0.061641 |
| IGHV4-59 | - | - | - | - | 4.06 | 9.33E-05 |
| IGKC | - | - | - | - | 2.24 | 0.002257 |
| IGKV1-13 | - | - | - | - | 2.93 | 0.054055 |
| IGKV1-17 | - | - | - | - | 2.83 | 0.042173 |
| IGKV1-6 | - | - | - | - | 2.97 | 0.013541 |
| IGKV1D-13 | - | - | - | - | 2.84 | 0.043716 |
| IGKV1D-17 | - | - | - | - | 3.00 | 0.076375 |
| IGKV2-24 | - | - | - | - | 3.60 | 0.00056 |
| IGKV6-21 | - | - | - | - | 3.68 | 0.07079 |
| IGLC5 | - | - | - | - | 3.04 | 0.036586 |
| LGALS9 | - | - | - | - | 2.53 | 0.026652 |
| LY96 | - | - | - | - | 2.17 | 0.011457 |
| PGLYRP1 | - | - | - | - | 2.61 | 0.003374 |
| RNASE2 | - | - | - | - | 2.20 | 0.020939 |
| S100A12 | - | - | - | - | 2.35 | 0.007647 |
| SPI1 | - | - | - | - | 2.06 | 0.021221 |
