# Supplementary material for: The expression patterns of immune response genes in the Peripheral Blood Mononuclear cells of pregnant women presenting with subclinical or clinical HEV infection are different and trimester-dependent: A whole transcriptome analysis
Source: PLoS One. 2020 Feb 3;15(2):e0228068. doi: 10.1371/journal.pone.0228068 (PMC6996850; doi:10.1371/journal.pone.0228068)
Supplement: S11 Table — (DOCX) [file pone.0228068.s013.docx]

**Significantly altered genes in acute and subclinical HEV infections in the pregnant women (2^nd^ trimester) with pair-wise comparison done with respective healthy pregnant controls**

**Table S13- List of up-regulated genes:**

| **Gene short name** | **PR-2-acute** | | **PR-2-SC** | |
| --- | --- | --- | --- | --- |
|  | **Fold change** | **Q value** | **Fold change** | **Q value** |
| ARHGDIA | 1.60 | 0.010745 | 1.91 | 1.7E-06 |
| CXCR3 | 2.20 | 0.024832 | 1.58 | 0.074489 |
| DUSP22 | 1.67 | 0.077381 | 1.53 | 0.01727 |
| IGHD | 2.14 | 0.000337 | 2.13 | 1E-06 |
| IGHV3-11 | 2.20 | 0.067041 | 1.95 | 0.02796 |
| IGHV3-23 | 2.74 | 6.41E-05 | 1.55 | 0.015241 |
| IGHV3-72 | 3.31 | 0.025208 | 3.03 | 0.01501 |
| IGLV3-1 | 4.00 | 1.46E-08 | 2.57 | 1.21E-05 |
| MEFV | 2.24 | 0.01121 | 2.07 | 0.002334 |
| MMP9 | 3.59 | 0.052384 | 3.53 | 0.035078 |
| PRKCSH | 1.57 | 0.010091 | 1.67 | 2.35E-05 |
| TICAM1 | 1.87 | 0.014249 | 1.83 | 0.000454 |
| VAV1 | 1.54 | 0.032939 | 1.48 | 0.002252 |
| ZFP36 | 1.75 | 0.001889 | 2.18 | 1.75E-09 |
| C4BPA | 4.18 | 0.040978 | - | - |
| CD180 | 1.75 | 0.008552 | - | - |
| CEACAM6 | 1.71 | 0.047286 | - | - |
| CXCL1 | 2.61 | 0.037048 | - | - |
| CXCL13 | 1.8E+308 | 0.074389 | - | - |
| CXCR1 | 3.23 | 3.08E-05 | - | - |
| DDX58 | 1.88 | 0.033716 | - | - |
| DEFA4 | 1.62 | 0.089063 | - | - |
| DHX40 | 1.22 | 0.081451 | - | - |
| IGHG3 | 1.63 | 0.039001 | - | - |
| IGHV1-18 | 2.20 | 0.053091 | - | - |
| IGHV1-69 | 2.28 | 0.041935 | - | - |
| IGHV3-21 | 2.85 | 0.003832 | - | - |
| IGHV3-33 | 2.68 | 0.002023 | - | - |
| IGHV3-48 | 2.20 | 0.065367 | - | - |
| IGHV3-49 | 2.54 | 0.071557 | - | - |
| IGHV3-53 | 2.80 | 0.024548 | - | - |
| IGHV4-31 | 2.39 | 0.082918 | - | - |
| IGJ | 3.48 | 0.040866 | - | - |
| IGKV1-12 | 2.79 | 0.003736 | - | - |
| IGKV1-39 | 2.41 | 0.003668 | - | - |
| IGKV1D-12 | 2.60 | 0.050902 | - | - |
| IGKV1D-16 | 2.21 | 0.097624 | - | - |
| IGKV2-28 | 2.20 | 0.031119 | - | - |
| IGLV6-57 | 2.92 | 0.020789 | - | - |
| IGLV8-61 | 2.59 | 0.011509 | - | - |
| LEP | 2.92 | 0.000334 | - | - |
| MPO | 2.44 | 0.001328 | - | - |
| NLRC4 | 1.71 | 0.087948 | - | - |
| PI3 | 3.42 | 2.5E-06 | - | - |
| TLR8 | 1.59 | 0.008444 | - | - |
| TNFAIP6 | 2.87 | 0.010293 | - | - |
| TNFRSF10D | 1.77 | 0.0299 | - | - |
| TNFSF10 | 2.08 | 0.095435 | - | - |
| UBA2 | 1.64 | 0.014096 | - | - |
| UQCRB | 2.30 | 0.000213 | - | - |
| VSIG4 | 2.34 | 0.018627 | - | - |
| ARHGEF18 | - | - | 1.55 | 3.75E-05 |
| CD1C | - | - | 1.28 | 0.063111 |
| CD74 | - | - | 2.18 | 0.063854 |
| CD79A | - | - | 1.15 | 0.029797 |
| CD97 | - | - | 1.47 | 0.01124 |
| DUSP23 | - | - | 1.91 | 0.017725 |
| HLA-DQB2 | - | - | 1.15 | 0.086978 |
| IFI35 | - | - | 2.34 | 0.019625 |
| IGHV3-9 | - | - | 1.82 | 0.099613 |
| IKBKAP | - | - | 1.25 | 0.097619 |
| IL1B | - | - | 2.27 | 0.000929 |
| NUAK2 | - | - | 1.40 | 0.004132 |
| PLAGL2 | - | - | 1.01 | 0.073106 |
| PTGS2 | - | - | 1.72 | 0.018406 |
| RPTOR | - | - | 1.73 | 0.015215 |
| SP2 | - | - | 2.23 | 0.000116 |
| SPI1 | - | - | 1.92 | 0.000177 |
| TNF | - | - | 3.13 | 0 |
