# Supplementary material for: The expression patterns of immune response genes in the Peripheral Blood Mononuclear cells of pregnant women presenting with subclinical or clinical HEV infection are different and trimester-dependent: A whole transcriptome analysis
Source: PLoS One. 2020 Feb 3;15(2):e0228068. doi: 10.1371/journal.pone.0228068 (PMC6996850; doi:10.1371/journal.pone.0228068)
Supplement: S12 Table — (DOCX) [file pone.0228068.s014.docx]

**Table S14- List of down-regulated genes:**

| **Gene short name** | **PR-2-acute** | | **PR-2-SC** | |
| --- | --- | --- | --- | --- |
|  | **Fold change** | **Q value** | **Fold change** | **Q value** |
| DEFA5 | -3.72 | 0.040604 | -3.67 | 0.013606 |
| GADD45B | -1.41 | 0.032149 | -1.50 | 0.000162 |
| IER5L | -2.68 | 0.031873 | -1.52 | 0.065305 |
| MTRNR2L9 | -7.43 | 5.95E-06 | -10.14 | 0.025646 |
| NDUFA7 | -2.19 | 0.045217 | -2.04 | 0.00543 |
| PF4 | -2.35 | 4.89E-05 | -3.84 | 0 |
| RHOB | -1.61 | 0.016357 | -1.16 | 0.008148 |
| RPS19 | -2.85 | 1.81E-07 | -3.74 | 0 |
| S100A11 | -1.52 | 0.017202 | -1.90 | 5.25E-07 |
| SOCS1 | -2.54 | 0.060776 | - | - |
| CAMP | - | - | -1.86 | 1.42E-05 |
| CCL2 | - | - | -1.89 | 0.018532 |
| CD63 | - | - | -1.42 | 0.087993 |
| COX5B | - | - | -2.46 | 2.03E-05 |
| CXCL5 | - | - | -1.34 | 0.003455 |
| DEFA1 | - | - | -1.47 | 0.079094 |
| DYNLRB1 | - | - | -1.23 | 0.041731 |
| FCER1G | - | - | -1.13 | 0.04435 |
| FIS1 | - | - | -2.56 | 0.005747 |
| G0S2 | - | - | -1.35 | 0.001905 |
| GZMA | - | - | -1.15 | 0.021031 |
| HLA-G | - | - | -1.60 | 0.001437 |
| IFNG | - | - | -1.45 | 0.002541 |
| JUND | - | - | -1.21 | 0.005755 |
| LGALS1 | - | - | -2.83 | 0.01344 |
| NDUFA13 | - | - | -3.06 | 2.78E-05 |
| NDUFB11 | - | - | -1.39 | 0.065577 |
| NDUFS4 | - | - | -1.75 | 0.016493 |
| NDUFS7 | - | - | -2.24 | 0.058796 |
| PLAUR | - | - | -1.32 | 0.001833 |
| POMP | - | - | -1.53 | 0.001587 |
| PSMA7 | - | - | -1.20 | 0.076425 |
| RPS6 | - | - | -2.11 | 0.001354 |
| S100A10 | - | - | -1.99 | 0.000115 |
| S100A8 | - | - | -2.60 | 1.11E-09 |
| S100A9 | - | - | -2.12 | 7.55E-05 |
| SERPINB2 | - | - | -1.65 | 0.033454 |
| SOD1 | - | - | -1.83 | 0.001994 |
| SUMO2 | - | - | -1.44 | 0.000313 |
| TIMP1 | - | - | -2.26 | 0.00067 |
| TMSB4X | - | - | -1.90 | 0.035956 |
| UBB | - | - | -1.68 | 0.033588 |
| UBE2B | - | - | -1.95 | 0.047576 |
| UQCR11 | - | - | -1.76 | 1.8E-05 |
| USMG5 | - | - | -3.04 | 4.63E-08 |
