# Supplementary material for: The expression patterns of immune response genes in the Peripheral Blood Mononuclear cells of pregnant women presenting with subclinical or clinical HEV infection are different and trimester-dependent: A whole transcriptome analysis
Source: PLoS One. 2020 Feb 3;15(2):e0228068. doi: 10.1371/journal.pone.0228068 (PMC6996850; doi:10.1371/journal.pone.0228068)
Supplement: S13 Table — (DOCX) [file pone.0228068.s015.docx]

**Significantly altered genes in acute and subclinical HEV infections in the pregnant women (3^rd^ trimester) with pair-wise comparison done with respective healthy pregnant controls**

**Table S15- List of up-regulated genes:**

| **Gene short name** | **PR-3-acute** | | **PR-3-SC** | |
| --- | --- | --- | --- | --- |
|  | **Fold change** | **Q value** | **Fold change** | **Q value** |
| ARHGDIA | 2.22 | 0.000824 | 2.04 | 6.31E-05 |
| CEACAM8 | 3.74 | 1.05E-08 | 1.54 | 0.071703 |
| DEFA4 | 2.97 | 6.19E-05 | 1.73 | 0.037042 |
| DUSP22 | 1.86 | 0.081514 | 1.89 | 0.012014 |
| ELANE | 3.06 | 0.043815 | 2.53 | 0.090559 |
| IGHA1 | 2.70 | 0.000138 | 1.51 | 0.036406 |
| IGHV3-23 | 3.52 | 1.87E-06 | 1.79 | 0.046819 |
| IGHV4-59 | 4.06 | 9.33E-05 | 2.82 | 0.010461 |
| LGALS9 | 2.53 | 0.026652 | 1.94 | 0.056364 |
| MEFV | 2.68 | 0.005444 | 1.89 | 0.043815 |
| MPO | 3.67 | 2.93E-08 | 2.37 | 0.00031 |
| P2RX1 | 2.18 | 0.000354 | 2.05 | 2.05E-05 |
| PRKCSH | 2.24 | 0.00067 | 1.77 | 0.001788 |
| RNASE2 | 2.20 | 0.020939 | 1.86 | 0.015511 |
| SPI1 | 2.06 | 0.021221 | 2.19 | 0.000654 |
| TGM2 | 2.02 | 0.061829 | 2.02 | 0.011328 |
| TICAM1 | 2.57 | 0.001001 | 1.99 | 0.007423 |
| VAV1 | 1.97 | 0.005779 | 1.61 | 0.007721 |
| ZFP36 | 2.19 | 0.001085 | 2.47 | 2.85E-08 |
| BPI | 3.70 | 0.00021 | - | - |
| CD180 | 2.35 | 0.000575 | - | - |
| CD300LB | 1.48 | 0.054427 | - | - |
| CEACAM6 | 3.36 | 2.54E-06 | - | - |
| CXCR1 | 3.37 | 2.82E-05 | - | - |
| CYP27A1 | 2.03 | 0.075184 | - | - |
| CYP4F3 | 2.49 | 0.062394 | - | - |
| IGHA2 | 2.44 | 0.001661 | - | - |
| IGHG1 | 2.20 | 0.013336 | - | - |
| IGHG2 | 2.06 | 0.010353 | - | - |
| IGHG3 | 2.35 | 0.002367 | - | - |
| IGHG4 | 2.44 | 0.003302 | - | - |
| IGHGP | 2.03 | 0.030329 | - | - |
| IGHM | 1.54 | 0.091274 | - | - |
| IGHV1-18 | 2.29 | 0.087608 | - | - |
| IGHV1-2 | 2.30 | 0.083341 | - | - |
| IGHV1-69 | 3.10 | 0.029514 | - | - |
| IGHV3-11 | 3.25 | 0.004361 | - | - |
| IGHV3-21 | 2.27 | 0.064839 | - | - |
| IGHV3-30 | 2.60 | 0.005572 | - | - |
| IGHV3-33 | 2.78 | 0.004549 | - | - |
| IGHV3-48 | 3.25 | 0.002099 | - | - |
| IGHV3-49 | 3.76 | 0.048892 | - | - |
| IGHV3-53 | 2.94 | 0.069807 | - | - |
| IGHV3-7 | 2.52 | 0.035003 | - | - |
| IGHV3-72 | 3.66 | 0.013303 | - | - |
| IGHV3-74 | 2.96 | 0.066317 | - | - |
| IGHV3-9 | 2.57 | 0.072926 | - | - |
| IGHV4-39 | 2.97 | 0.061641 | - | - |
| IGHV6-1 | 3.89 | 0.001972 | - | - |
| IGJ | 3.68 | 0.038475 | - | - |
| IGKC | 2.24 | 0.002257 | - | - |
| IGKV1-12 | 3.43 | 0.000354 | - | - |
| IGKV1-13 | 2.93 | 0.054055 | - | - |
| IGKV1-16 | 3.75 | 0.000986 | - | - |
| IGKV1-17 | 2.83 | 0.042173 | - | - |
| IGKV1-39 | 2.76 | 0.001647 | - | - |
| IGKV1-5 | 2.11 | 0.01933 | - | - |
| IGKV1-6 | 2.97 | 0.013541 | - | - |
| IGKV1-9 | 2.34 | 0.086445 | - | - |
| IGKV1D-12 | 2.84 | 0.05904 | - | - |
| IGKV1D-13 | 2.84 | 0.043716 | - | - |
| IGKV1D-16 | 3.78 | 0.003742 | - | - |
| IGKV1D-17 | 3.00 | 0.076375 | - | - |
| IGKV2-24 | 3.60 | 0.00056 | - | - |
| IGKV2-28 | 3.02 | 0.001188 | - | - |
| IGKV4-1 | 2.18 | 0.009305 | - | - |
| IGKV6-21 | 3.68 | 0.07079 | - | - |
| IGLC2 | 1.54 | 0.090726 | - | - |
| IGLC3 | 1.53 | 0.094163 | - | - |
| IGLC5 | 3.04 | 0.036586 | - | - |
| IGLV3-1 | 1.90 | 0.047045 | - | - |
| LEP | 3.31 | 1.53E-05 | - | - |
| LY96 | 2.17 | 0.011457 | - | - |
| MMP9 | 4.41 | 0.000368 | - | - |
| NLRC4 | 2.45 | 0.012498 | - | - |
| PGLYRP1 | 2.61 | 0.003374 | - | - |
| PI3 | 2.96 | 0.00439 | - | - |
| S100A12 | 2.35 | 0.007647 | - | - |
| SLPI | 2.03 | 0.089179 | - | - |
| TLR8 | 1.99 | 0.004608 | - | - |
| TNFAIP6 | 3.58 | 0.0004 | - | - |
| TNFSF10 | 2.39 | 0.02906 | - | - |
| UQCRB | 2.35 | 0.003273 | - | - |
| ACTG1 | - | - | 1.36 | 0.03667 |
| ARHGEF18 | - | - | 1.47 | 0.00985 |
| CCL3L3 | - | - | 2.10 | 0.0262 |
| CD68 | - | - | 1.51 | 0.030672 |
| CD74 | - | - | 2.64 | 0.087293 |
| CD97 | - | - | 1.94 | 0.000808 |
| CXCR3 | - | - | 2.21 | 0.029675 |
| EP300 | - | - | 1.22 | 0.045583 |
| IER2 | - | - | 2.05 | 0.00012 |
| IGHD | - | - | 2.05 | 0.00078 |
| IGLV8-61 | - | - | 2.12 | 0.084504 |
| IRF2BP2 | - | - | 2.27 | 0.022663 |
| LGALS2 | - | - | 1.41 | 0.026985 |
| MAP2K1 | - | - | 1.65 | 0.08528 |
| NUAK2 | - | - | 1.51 | 0.029655 |
| PDLIM1 | - | - | 2.25 | 0.004085 |
| SIGLEC12 | - | - | 2.81 | 0.023297 |
| SMAD4 | - | - | 1.80 | 0.09353 |
| TBX21 | - | - | 1.54 | 0.018014 |
| TNF | - | - | 3.20 | 4.25E-10 |
| TNFRSF21 | - | - | 1.89 | 0.060444 |
| TRIM58 | - | - | 2.43 | 0.000321 |
