# Supplementary material for: The expression patterns of immune response genes in the Peripheral Blood Mononuclear cells of pregnant women presenting with subclinical or clinical HEV infection are different and trimester-dependent: A whole transcriptome analysis
Source: PLoS One. 2020 Feb 3;15(2):e0228068. doi: 10.1371/journal.pone.0228068 (PMC6996850; doi:10.1371/journal.pone.0228068)
Supplement: S14 Table — (DOCX) [file pone.0228068.s016.docx]

**Table S16 List of Primer sequences used for SYBR green-based Real Time PCR assays**

| **Gene short name** | **Primer sequence (5’to3’)** |
| --- | --- |
| DEFA1 FORWARD | TCCCTTGCATGGGACGAAAG |
| DEFA1 REVERSE | GGTTCCATAGCGACGTTCTCC |
| DEFA4 FORWARD | CCTTTGCATGGGATAAAAGCTCT |
| DEFA4 REVERSE | ACACCACCAATGAGGCAGTTC |
| S100A6 FORWARD | GGGAGGGTGACAAGCACAC |
| S100A6REVERSE | AGCTTCGAGCCAATGGTGAG |
| S100A8 FORWARD | ATGCCGTCTACAGGGATGAC |
| S100A8 REVERSE | ACTGAGGACACTCGGTCTCTA |
| S100A9 FORWARD | GGTCATAGAACACATCATGGAGG |
| S100A9 REVERSE | GGCCTGGCTTATGGTGGTG |
| S100A12 FORWARD | AGCATCTGGAGGGAATTGTCA |
| S100A12 REVERSE | GCAATGGCTACCAGGGATATGAA |
| IGJ FORWARD | TCCTGGCGGTTTTTATTAAGGC |
| IGJ REVERSE | AGTAATCCGGGCACACTTACAT |
